# eCarbonyls: an electrochemical thioether mediated oxidation of alcohols to aldehydes and ketones

**DOI:** 10.1039/d5sc06546a

**Published:** 2025-10-06

**Authors:** Conall Molloy, Simon Kaltenberger, Lee Edwards, Katherine M. P. Wheelhouse, Kevin Lam

**Affiliations:** a Faculty of Engineering and Science, University of Greenwich Grenville Building, Central Avenue Chatham ME4 4TB UK k.lam@greenwich.ac.uk; b Otto Diels-Institut für Organische Chemie, Christian-Albrechts-Universität zu Kiel Otto-Hanhn-Platz 4 24098 Kiel Germany; c GSK Medicines Research Centre Gunnels Wood Road Stevenage Hertfordshire SG1 2NY UK

## Abstract

We report eCarbonyls, a scalable, metal-free electrochemical oxidation of alcohols that mimics key features of the classical Swern reaction while avoiding its reliance on cryogenic conditions and hazardous reagents. Operating at room temperature in an undivided cell, this process employs a stable thioether mediator to generate reactive radical cation intermediates that enable selective oxidation of primary and secondary alcohols to aldehydes and ketones. The method displays broad substrate scope, with up to 98% isolated yields across more than 25 examples, and excellent tolerance toward sensitive functional groups, including azides, boronates, and silyl ethers. Mechanistic studies confirm the role of anodically generated thioether radical cations and highlight the importance of the external base. Notably, eCarbonyls is readily scalable and adaptable to flow electrolysis, enabling multigram synthesis and offering a safe, sustainable platform for academic and industrial applications.

## Abstract 2

We report eCarbonyls, a scalable, metal-free electrochemical oxidation of alcohols that mimics key features of the classical Swern reaction while avoiding its reliance on cryogenic conditions and hazardous reagents.

## Graphical abstract

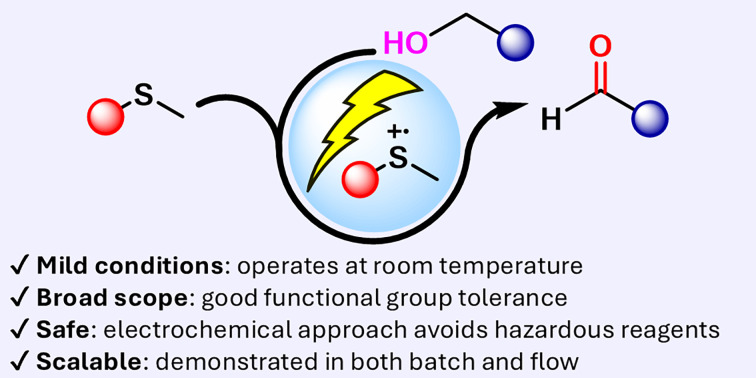

Aldehydes and ketones are key functional groups found in a broad range of molecules, including pharmaceuticals, agrochemicals, polymers, fragrances, and natural products.^1–3^ Their widespread presence and synthetic versatility make them indispensable building blocks in modern organic chemistry. As such, the development of efficient, selective, and sustainable methods for accessing these carbonyl compounds remains a central challenge for both academic and industrial chemists.

The most direct route to these carbonyl compounds remains the oxidation of alcohols, a process that is performed on a multi-kilogram scale across the chemical industry.^4^ Despite its apparent simplicity, alcohol oxidation is often operationally complex and environmentally burdensome. Traditional methods, including those based on stoichiometric chromium(vi) reagents, Dess–Martin periodinane, manganese dioxide, or Swern conditions, are well established, but they typically generate large quantities of hazardous waste or rely on cryogenic conditions (Scheme 1). They also often lack compatibility with sensitive functional groups, severely limiting their scalability, sustainability and compliance with green chemistry principles.^5–8^

**Scheme 1:**
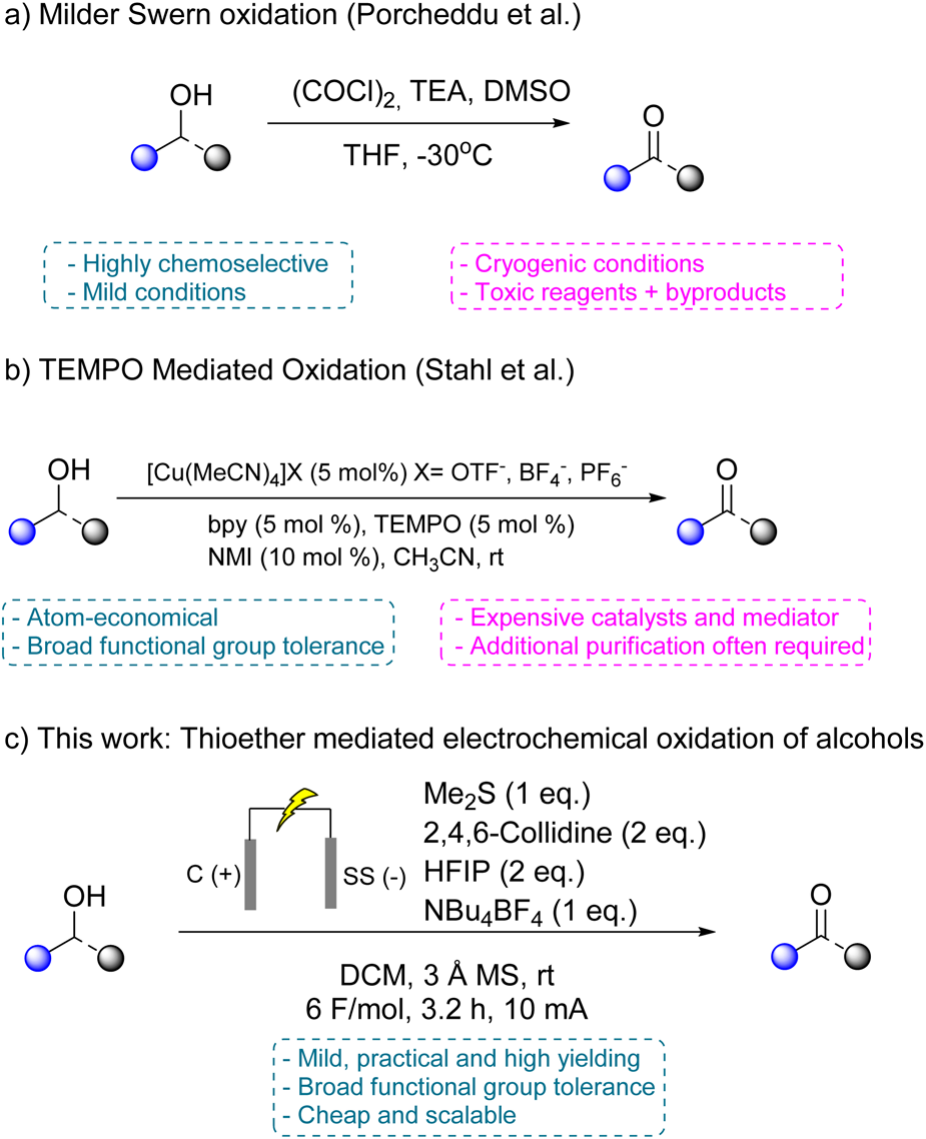
Advantages and drawbacks of traditional oxidation methods compared to this work.

The Swern oxidation is widely valued for its chemoselectivity and mild reaction conditions. However, its operational requirements—including the use of oxalyl chloride, dimethyl sulfoxide, and cryogenic temperatures render it impractical for large-scale applications or use in settings with limited infrastructure.^9^ As a result, considerable effort has been devoted to developing greener alternatives, particularly catalytic aerobic oxidations that use molecular oxygen or air as terminal oxidants. These methods typically employ transition metals such as copper, ruthenium, or palladium, in combination with redox mediators like TEMPO (Scheme 1).^10^ While these systems have enabled significant advances, they often require elevated pressures, specialised ligands, and a thorough post-reaction purification to remove residual catalysts. Other TEMPO-mediated oxidations, such as the Anelli oxidation, face challenges in scale-up because they employ bleach as the terminal oxidant, although they have nevertheless found applications in the pharmaceutical industry.^11^ Aerobic photooxidation of alcohols using molecular oxygen has gained increasing attention in recent years; however, most reported protocols remain limited to aromatic or otherwise activated substrates, and from an industrial perspective the use of oxygen (even air) is generally avoided due to inherent safety concerns.^12–14^ Reactions involving flammable organic solvents may easily exceed the solvent's flash point, introducing a substantial risk of ignition or explosion under scale-up conditions.^15^ Biocatalytic oxidations of alcohols remain relatively niche in industry, with most applications confined to the perfume sector. This limited uptake reflects practical challenges such as extracting aldehydes from aqueous media, which often requires multiple back-extractions, making large-scale processes cumbersome and costly.^16^

In this context, electrochemical oxidation has emerged as a promising strategy to meet the dual goals of reactivity and sustainability.^17,18^ Electrochemical methods replace traditional oxidants with electricity, offering reagent-free redox control under tuneable, mild conditions. This avoids many of the drawbacks associated with chemical oxidants, reduces waste generation and enables straightforward scaling up using flow systems. However, many reported electrochemical alcohol oxidations have limited scope. They often require expensive electrode materials or halide mediators, display substrate scopes limited to aromatic alcohols and exhibit poor selectivity for primary alcohols, particularly in the formation of aldehydes.^19–22^

Here, we present a practical, scalable and sustainable sulfur mediated electrochemical Swern-type oxidation process (eCarbonyls), which efficiently converts primary and secondary alcohols into their respective aldehydes and ketones. Our approach uses a stable thioether mediator in an undivided electrochemical cell, avoids cryogenics and toxic reagents, and operates under mild, metal-free conditions. The reaction proceeds cleanly at ambient temperature and offers a broad functional group compatibility. It is also readily adaptable to flow systems, highlighting its potential for laboratory and industrial applications.

As a starting point for our investigations, we examined the electrochemical oxidation of 2-adamantylethanol using a thioether mediator to produce the corresponding aldehyde. After extensive optimisation of the reaction parameters, we developed a robust protocol for oxidising 2-adamantylethanol, yielding aldehyde 1a with a 92% yield.

The reaction was performed using inexpensive graphite and stainless steel (SS) electrodes with a constant current of 10 mA. We employed a combination of 2,4,6-collidine as the base, hexafluoroisopropanol (HFIP) as the additive and dichloromethane (DCM) as the solvent, along with NBu_4_BF_4_ as the supporting electrolyte. Each component of the reaction proved essential for achieving high conversion and selectivity. While a full optimisation table is provided in the SI, salient results are summarised in Table 1.

**Table 1:** Selected results from the optimization experiments. Reactions carried out on a 0.2 mmol scale. Full optimization table available in the SI

Entry	Deviation from standard conditions	Yield of 1b^a^
1	None	92
2	No thioether	7
3	No 2,4,6-collidine	15
4	No 3 Å molecular sieves (MS)	70
5	No HFIP	73
6	No electricity	0
7	PhMeS as mediator	91

a Yields were calculated *via* GCMS using a calibration curve.

Control experiments revealed the importance of the presence of the thioether mediator and collidine base: omitting either resulted in a significant decrease in product yield (7% and 15%, respectively; see entries 2 and 3). Excluding 3 Å molecular sieves also significantly reduced the yield (entry 4, 70%), likely due to residual water promoting the undesired oxidation of the thioether to its sulfoxide and thereby deactivating the mediator. Interestingly, the inclusion of HFIP led to a significant increase in yield (entry 5), which we attribute to its ability to suppress undesired cathodic side reactions. This effect is likely due to HFIP acting as a proton donor, facilitating hydrogen evolution at the stainless steel cathode, which possesses a relatively low hydrogen overpotential.^23^ Finally, a control reaction carried out in the absence of applied current produced no conversion (entry 6), confirming the electrochemical nature of the transformation. Thioanisole, which is less volatile than dimethyl sulfide, was also tested as a mediator (entry 7) and afforded comparable yields. However, its use required chromatographic purification after workup to isolate the aldehyde and remove sulfoxide by-products, so dimethyl sulfide was selected for the scope studies due to its simpler purification profile.

With the best conditions in hand, we proceeded to explore the scope and limitations of this novel electrochemically mediated alcohol oxidation (Fig. 1). We evaluated a broad range of primary and secondary alcohols bearing diverse functional groups under the standard conditions (Scheme 2). Both primary and secondary aliphatic alcohols were smoothly converted to the corresponding carbonyl compounds with excellent yields, reaching up to 98% (1a–1c). Substituted phenylethanol derivatives (1e–1h) also underwent clean oxidation, providing products with yields of 78–96%. These results suggest that electronic and steric modifications to the aromatic ring, including electron-donating (*e.g.* methoxy 1g, 1h) and electron-withdrawing (*e.g.* cyano 1l) substituents, have a minimal impact on the reaction.

**Fig. 1:**
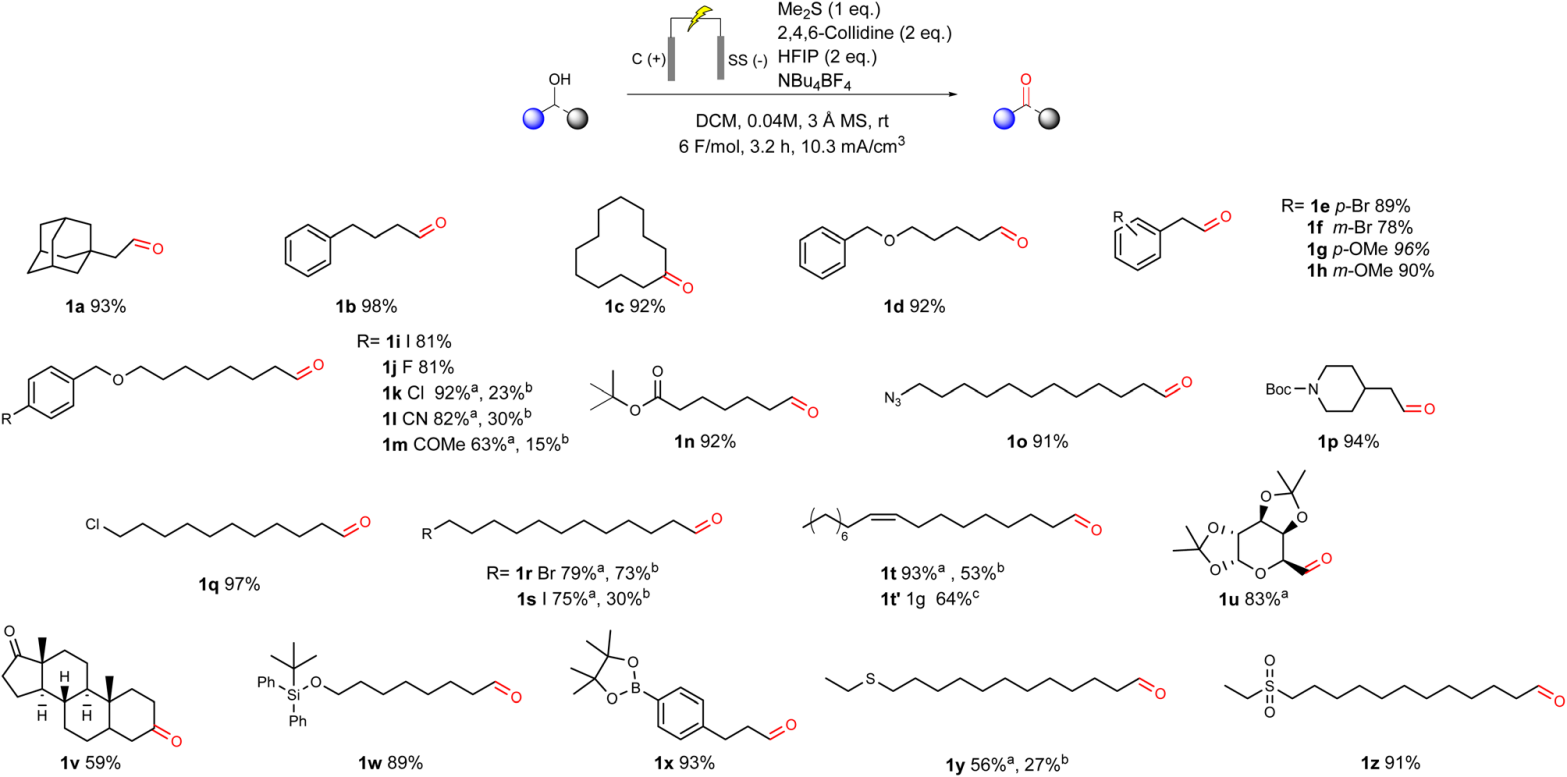
Substrate scope for the sulfur mediated electrochemical oxidation of alcohols to aldehydes and ketones. Reactions were carried out on a 0.2 mmol scale. Unless otherwise indicated all yields are from isolated compounds after an acidic workup. ^a^NMR yield of the crude product after acidic workup. ^b^ Isolated yield after column chromatography. ^c^ 1 g scale reaction was carried out in a 20 cm^3^ eSyn vial at 0.24 M scale. The reaction was electrolyzed at a constant current of 10 mA for 3 F before full conversion of the alcohol was seen *via* GCMS. The isolated yield from flash column chromatography is reported.

**Scheme 2:**
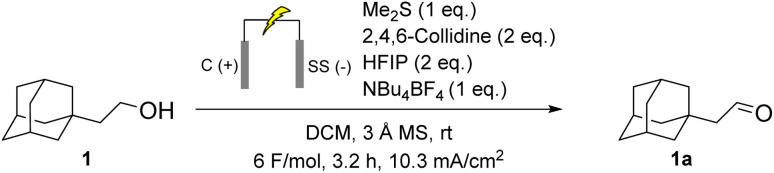
Standard reaction conditions for the eCarbonyls reaction.

The ether-containing substrate 1d was obtained in a 92% yield, prompting the exploration of further para-substituted analogues (1i–1m). These substrates exhibited full conversion, as confirmed by GC-MS after 6 F mol^−1^. While aldehydes were obtained in high yield with acceptable purity after workup (see SI), substantial losses were observed during purification in the cases that required it (1k–1m). These losses are attributed to the inherent instability of aldehydes on silica gel during small-scale flash chromatography, as has been extensively reported in the literature.^24–26^

More sensitive functional groups revealed limitations. Substrates bearing free ketones (1m) showed low yields of the desired aldehyde and underwent further degradation during chromatographic purification over silica gel, resulting in poor isolated yields. In contrast, azides and protected amines (1o and 1p) were well tolerated, with isolated yields exceeding 90%. Aliphatic chlorides (1q) also displayed excellent compatibility, delivering the product with a near-quantitative yield of 97%. However, the corresponding bromide (1r) and iodide (1s) analogues lead to lower yields, presumably due to the weaker C–Br and C–I bonds, and their increased propensity for side reactions under electrochemical conditions. These substrates also required further purification.

To evaluate scalability and address the limitations of small-scale purification, octadec-9-enal (1t) was selected for gram-scale synthesis (1t). Despite the detection of minor overoxidation side product *via* GC-MS, full conversion of the starting material was achieved and the isolated yield increased to 63%, which is a significant improvement on that obtained at the 0.2 mmol scale.

The method's tolerance to functional groups was further evaluated using complex substrates bearing groups that could potentially interact with the electrogenerated sulfonium intermediate. A protected sugar alcohol (1u) was oxidised to the corresponding aldehyde with a high crude yield. However, significant losses occurred during silica gel chromatography purification, which is consistent with earlier observations. The steroid derivative (1v) was converted with moderate success, providing the desired aldehyde with an isolated yield of 59%. Notably, more sensitive functional groups, such as silyl ethers (1w) and boronate esters (1x), were well tolerated under the reaction conditions. These delivered the corresponding aldehydes in excellent yields, thereby demonstrating the mildness and broad compatibility of the protocol.

Unsurprisingly, an alcohol bearing a thioether functionality (1y) produced a highly complex crude mixture. This is presumably due to the structural similarity between the thioether moiety and the sulfide-based electrochemical mediator, which makes selective oxidation of the intended mediator challenging. To overcome this issue, the thioether was pre-oxidised to the corresponding sulfone (1z). This modification successfully suppressed competing side reactions, affording the desired aldehyde in 91% yield without the need for further purification following workup, thereby supporting the proposed rationale.

Several functional groups were found to be incompatible with the eCarbonyls oxidation (Scheme 3). Benzylic-alcohols (2a) were completely consumed, as confirmed by GC-MS; however, no formation of the corresponding aldehyde was detected. Similarly, free carboxylic acids and terminal alkynes (2b, 2c) failed to yield the desired products under the optimized conditions, with terminal alkynes leading to significant corrosion of the anode. A comprehensive list of alcohol substrates that afforded no or only trace amounts of the corresponding aldehydes is provided in the SI.

**Scheme 3:**
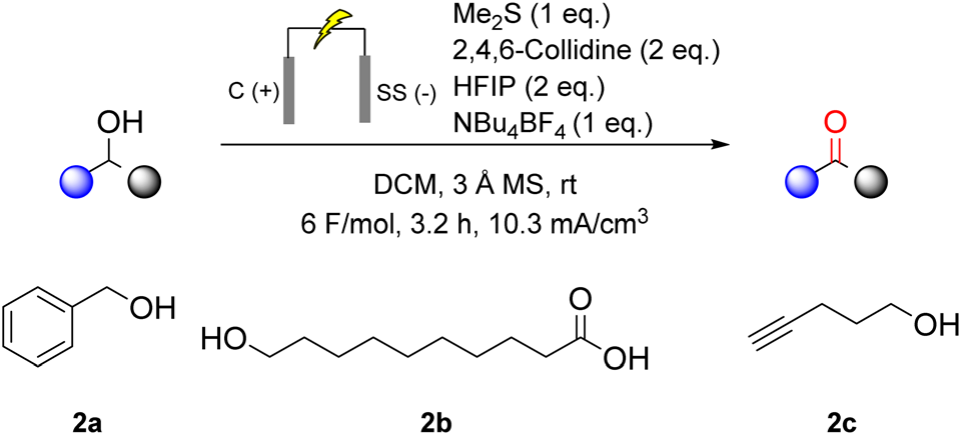
Select incompatible substrates with the eCarbonyls reaction.

As a proof of concept for scalability, the reaction was transposed to a continuous flow setup (Scheme 4). The optimised conditions delivered an 84% yield—comparable to batch mode with a space time yield of 5.99 × 10^−3^ kg h^−1^ L^−1^ (0.72 mmol h^−1^), underscoring the system's suitability for scale-up. Increasing the substrate concentration in an effort to boost productivity led to lower yields due to enhanced formation of overoxidation by-products. Full details of the optimisation studies are provided in the SI.

**Scheme 4:**
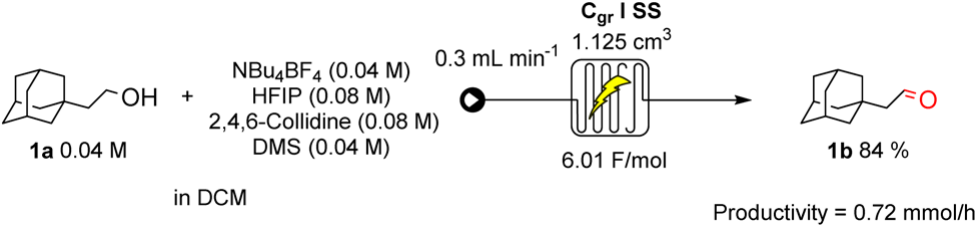
Optimised continuous flow conditions for the eCarbonyls reaction.

Next, we sought to gain a mechanistic understanding of the sulfide-mediated electrochemical oxidation of alcohols. We hypothesised that the reaction could proceed *via* one of two plausible pathways (path 1 and path 2). In both cases, anodic oxidation generates a sulfur radical cation that then interacts with the alcohol substrate to form an intermediate which collapses to the corresponding carbonyl compound upon deprotonation (see Scheme 5).

**Scheme 5:**
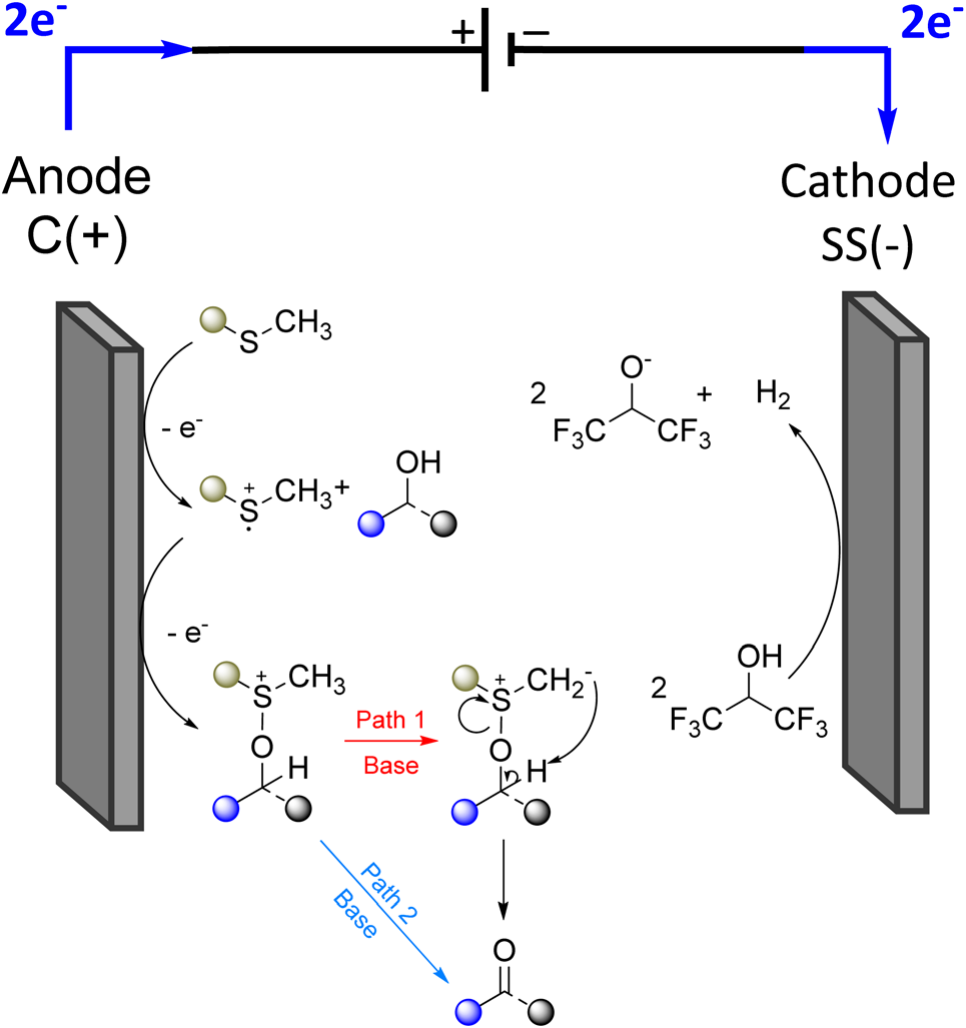
Proposed mechanism for the sulfur mediated electrochemical oxidation of alcohols.

Through investigation of the reaction by cyclic voltammetry a single oxidation peak was noted at *E*_pa_ = 1 V *vs.* Fc^+^/Fc, which is consistent with the literature values for the oxidation of dimethyl sulfide.^27^

To investigate the impact of the thioether's structure, diphenyl sulfide was used instead of dimethyl sulfide as the mediator (Scheme 6). Under these conditions no conversion of the alcohol was observed, suggesting that either an easily deprotonatable group is needed in alpha position of the thio centre, or that steric hindrance can be critical.

**Scheme 6:**
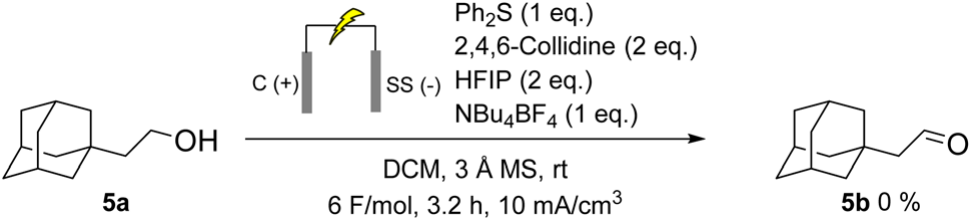
Sulfide mediated electrochemical oxidation of adamantyl ethanol with diphenyl sulfide as mediator.

To further clarify the mechanism, a labelling experiment was conducted using a deuterated alcohol (6a) and thioanisole as the mediator (Scheme 7). This experiment was designed to distinguish between an intermolecular deprotonation and one involving the formation of a sulfonium ylide intermediate. Upon completion of the electrolysis, ^1^H NMR analysis revealed no deuterium incorporation at the α-position of the mediator. This rules out the possibility of intramolecular proton (or deuteron) transfer from the alcohol to the sulfonium ylide (path 1), as in the classical chemical Swern oxidation, supporting a mechanism in which deprotonation is mediated by the external base 2,4,6-collidine rather than the sulfide itself (path 2).

**Scheme 7:**
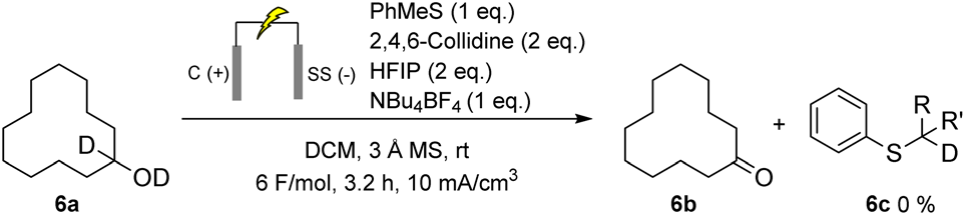
Electrochemical oxidation of deuterated cyclododecanol with methylphenylsulfane as a mediator. R, R′ = H, D.

An investigation was conducted into an alternative mechanism in which the thioether attaches to the alcohol through a methyl group to form 7a.^28^ This was tested by electrolysing 7a under the standard condition (Scheme 8). No conversion to aldehyde 7b was observed, invalidating this proposed mechanism.

**Scheme 8:**
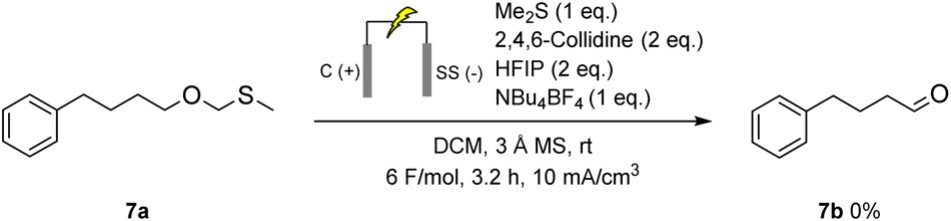
Electrochemical oxidation of deuterated cyclododecanol with methylphenylsulfane as a mediator. R, R′ = H, D.

## Conclusions

In summary, we have developed a practical electrochemical method for oxidising primary and secondary alcohols to the corresponding aldehydes and ketones using inexpensive dimethyl sulfide as a redox mediator. The protocol proceeds under mild conditions, delivers moderate to excellent yields across a broad substrate scope with good functional group tolerance, and is readily scalable. In flow, the method achieved a productivity of 0.72 mmol h^−1^. This operationally simple approach provides a valuable alternative to conventional oxidation methods and represents a versatile tool for modern synthetic chemistry.

## Author contributions

Conceptualization: K. L.; funding acquisition: K. L., K. W., L. E.; methodology: K. L., S. K., C. M.; investigation: S. K., C. M.; formal analysis: K. L., S. K., C. M., K. W., L. E.; resources: K. W., L. E.; supervision: K. L.; visualization: C. M.; writing – original draft: C. M.; writing – review & editing: all authors; industrial insight: K. W., L. E.

## Conflicts of interest

There are no conflicts to declare.

## Supplementary Material

SC-016-D5SC06546A-s001

## Data Availability

All data supporting the findings of this study, including experimental procedures, optimisation data, mechanistic studies, and NMR spectra, are provided in the SI. Supplementary information is available. See DOI: https://doi.org/10.1039/d5sc06546a.
