# Supplementary material for: eCarbonyls: an electrochemical thioether mediated oxidation of alcohols to aldehydes and ketones
Source: Chem Sci. 2025 Oct 6;16(43):20286–91. doi: 10.1039/d5sc06546a (PMC12499888; doi:10.1039/d5sc06546a)

Supplementary Information (SI) for Chemical Science.
This journal is © The Royal Society of Chemistry 2025

eCarbonyls: An Electrochemical Thioether Mediated Oxidation of Alcohols to Aldehydes and Ketones

Supporting Information

Conall Molloy[a]+, Simon Kaltenberger[b]+, Katherine Wheelhouse [c], Lee Edwards[c], Kevin Lam[a]*.

a. Faculty of Engineering and Science, University of Greenwich, Grenville Building, Central Avenue, Chatham, ME4 4TB

* Corresponding author: k.lam@greenwich.ac.uk

b. Otto Diels-Institut für Organische Chemie, Christian-Albrechts-Universität zu Kiel, Otto-Hanhn-Platz 4, 24098 Kiel, Germany.

c. GSK Medicines Research Centre, Gunnels Wood Road, Stevenage, Hertfordshire, SG1 2NY, UK

+ Co-first authors

Table of Contents

Contents

1. Material and methods 3
2. Optimisation studies 4
   2.1: Batch Optimisation Studies 4
   2.2: Flow Optimisation Studies 9
3. Experimental Procedures 11
   3.2: General Procedure B for the electrochemical oxidation of alcohols to aldehydes/ketones 14
   3.3: Synthesis of Materials for Mechanistic Studies 19
   3.4: Incompatible substrates 21
4. Mechanistic Studies 22
   4.1: Cyclic Voltammetry 22
   4.2: Deuterium labelled alcohol oxidation 23
5. References 24
6. NMR Spectra 25

1. Material and methods

All reactions were carried out under aerobic conditions unless otherwise stated. All solvents and commercially available reagents were purchased from standard vendors and used without further purification unless otherwise stated. Electrolyses were performed using an IKA Electrasyn 2.0 using carbon graphite working electrode and stainless steel counter electrode (distance between the electrodes = 6 mm) using a stirring rate of 600 rpm. Analytical thin-layer chromatography (TLC) was performed using silica gel plates (0.25 mm thickness) on aluminum support. Visualization was accomplished by irradiation with a UV lamp and/or staining with either KMnO4 or vanillin. Column chromatography was performed over Silica gel 60 Å (40-63µ mesh) using a CombiFlaswh Rf Lumen automatic flash chromatography system. Residual solvent was removed using a static oil pump (< 10 mbar).

NMR spectra were obtained using a JEOL ECZR 400 (1H 399.78 MHz; 19F 376.17 MHz; 13C 100.53 MHz) or ECA 500 (1H 500.16 MHz; 13C 125.77 MHz) spectrometer and are reported relative to the residual solvent resonances. All heteronuclear NMR spectra were 1Hdecoupled and recorded at room temperature unless otherwise stated. Data for 1H NMR spectra are reported as follows: chemical shift (δ, ppm), coupling constant (Hz), multiplicity (s, singlet; d, doublet; t, triplet; m, multiplet; br, broad) and integration. Data for 13C NMR are reported in terms of chemical shift (δ, ppm). IR spectra were recorded on a Perkin Elmer Spectrum Two instrument as neat samples.

High Resolution Mass Spectrometry (HRMS) data were obtained by Dr. Iain Goodall of the University of Greenwich Mass Spectrometry Service using a Waters Synapt G2 hybrid Quadrupole-orthogonal acceleration time-of-flight configuration (Waters, Manchester, UK) operating in Resolution Mode (M/ΔM ≥ 18,000), fitted with a Waters Acquity UPLC binary solvent chromatographic pump system. The column used was a reversed-phase Acquity BEH C18 2.1 x 50 mm, 1.7-micron bead, running a 3- minute separation with an A:B eluent mixture comprising of either deionised water with 0.1% (v:v) formic acid and acetonitrile with 0.1% (v:v) formic acid (negative mode) respectively or deionised water with 0.1% (v:v) ammonium hydroxide and acetonitrile with 0.1% (v:v) ammonium hydroxide (positive mode) respectively. Mass calibration of the instrument was performed using sodium formate cluster ions, and an orthogonal Lock-SprayTM ESI probe was used with a lock mass calibrant, leucine-enkephalin. The pseudomolecular leucine-enkephalin ion at m/z = 554.2615 (Negative Ion Mode), and m/z = 556.2771 (Positive Ion Mode), was used as the internal mass correction calibrant. Additional samples were analyzed on a Thermo LTQ Orbitrap XL coupled with a heated electrospray source (HESI). The capillary temperature was set to 275 °C and a voltage of 21 V. The sheath gas and auxiliary gas flow were set to 10 and 5 L h−1 respectively and the source current and voltage set to 100 µA and 5 kV. A solution of analyte (0.1 mg/ml) and sodium formate (1% v/v) in acetonitrile was added by direct infusion (10 µL/min) into the mass spectrometer using a Hamilton syringe (250 µL).

Gas-Chromatography Mass Spectrometry (GC-MS) data were obtained using a Shimadzu Nexis GC-2030 gas chromatograph connected to a GCMS-QP2020 NX gas chromatograph mass spectrometer, equipped with an AOC-20i Plus auto injector. The column was a CD-5MS capillary column (30 m x 0.25 mm x 0.25 µm), with helium as the carrier gas. The sample injection volume was 1 µL, and separations run over a 5-minute period with an increasing oven temperature (gradient) between 40 – 280 °C. Results were visualised and processed using LabSolutions GCMS solution version 4.50. High-Performance Liquid Chromatography-Mass Spectrometry (HPLC-MS) data were obtained using a Shimadzu LC-2050C 3D coupled with a Shimadzu LCMS-2020 FCV-20AH2. The column was an Ascentis Express 90Å AQ-C18, 2.7 µm. Results were visualised and processed using LabSolutions GCMS solution version 5.114. Cyclic voltammetry studies were carried out using an Autolab 302N potentiostat interfaced through Nova 2.1 software to a personal computer. Electrochemical measurements were performed in a glovebox under an atmosphere of dinitrogen with oxygen and water levels of less than 5 ppm at 298 K, with solvents that had been thoroughly degassed and purified by passing through an alumina-based purification system. Sample concentrations of 1.0 mM were used, alongside 0.1 M [nBu4N][PF6] supporting electrolyte concentrations. Experiments were conducted using a standard three-electrode setup comprising of a glassy carbon disc working electrode, platinum wire counter electrode, and AgCl coated silver wire as a pseudo-reference electrode. Potentials are reported relative to the [FeCp2] +/0 redox couple, obtained through the addition of ferrocene to the analyte solution.

Continuous flow reactions were carried out in an Ammonite 8 electrochemical flow cell (Cambridge Reactor Design) with a diameter of 149 mm and a spiral electrolyte flow channel 2000 mm long, 5 mm wide, and 0.5 mm interelectrode gap. Carbon graphite (C_{gr}) anode and stainless steel (SS) cathode were used for all reactions. The Ammonite 8 cell is commercially available from Cambridge Reactor Design (https://www.cambridgereactordesign.com/ammonite/ammonite.html).

2. Optimisation studies

2.1: Batch Optimisation Studies

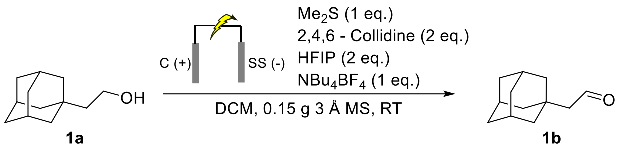

Scheme S1. Optimised reaction conditions for the eSwern reaction (Entry 38)

Table S1. Optimisation of the sulfide mediated electrochemical oxidation of alcohols.

Entry	Current [mA]	Eq. Electrons [F/mol]	Supporting Electrolyte (equiv.)	Base (equiv)	Thioether (equiv)	Anode	Cathode	Additive (equiv)/ Note	Solvent (mL)	**Yield [%]**
1	10	3	NBu_4ClO_4 (1.0)	2,6-Lutidine (2.0)	Ph-S-Me (1.0)	Pt	$C_{gr.}$	-	CH_2Cl_2 (5)	**41**
2	10	5	NBu_4ClO_4 (1.0)	2,6-Lutidine (2.0)	Ph-S-Me (1.0)	Pt	$C_{gr.}$	-	CH_2Cl_2 (5)	**56**
3	10	6	NBu_4ClO_4 (1.0)	2,6-Lutidine (2.0)	Ph-S-Me (1.0)	Pt	$C_{gr.}$	-	CH_2Cl_2 (5)	**57**
4	10	5	NBu_4BF_4 (1.0)	2,6-Lutidine (2.0)	Ph-S-Me (1.0)	Pt	$C_{gr.}$	-	CH_2Cl_2 (5)	**58**
5	10	5	NBu_4BF_4 (1.0)	2,6-Lutidine (2.0)	4-Methoxythioanisole (1.0)	Pt	$C_{gr.}$	-	CH_2Cl_2 (5)	**45**
6	10	5	NBu_4BF_4 (1.0)	2,6-Lutidine (2.0)	4-Nitrothioanisole (1.0)	Pt	$C_{gr.}$	-	CH_2Cl_2 (5)	**n.d.**
7	10	5	NBu_4BF_4 (1.0)	2,6-Lutidine (2.0)	Ph-S-Me (1.0)	Pt	$C_{gr.}$	-	CH_2Cl_2 (3)	**51**

8	10	6	NBu_4BF_4 (1.0)	2,6-Lutidine (2.0)	Ph-S-Me (1.0)	Pt	$C_{gr.}$	-	MeCN (5)	**9**
9	10	6	NBu_4BF_4 (1.0)	2,4,6-Collidine (2.0)	Ph-S-Me (1.0)	Pt	$C_{gr.}$	-	CH_2Cl_2 (5)	**64**
10	10	6	NBu_4BF_4 (1.0)	2,4,6-Collidine (2.0)	Ph-S-Me (1.0)	$C_{gr.}$	Pt	-	CH_2Cl_2 (5)	**69**
11	10	6	NBu_4BF_4 (1.0)	2,4,6-Collidine (2.0)	Ph-S-Me (1.0)	Pt	SS	-	CH_2Cl_2 (5)	**44**
12	10	6	NBu_4BF_4 (1.0)	2,4,6-Collidine (2.0)	Ph-S-Me (1.0)	Pt	Pt	-	CH_2Cl_2 (5)	**71**
13	10	6	NBu_4BF_4 (1.0)	2,4,6-Collidine (2.0)	Ph-S-Me (1.0)	$C_{gr.}$	SS	-	CH_2Cl_2 (5)	**66**
14	10	6	NBu_4BF_4 (1.0)	2,4,6-Collidine (2.0)	Ph-S-Me (1.0)	$C_{gr.}$	$C_{gr.}$	-	CH_2Cl_2 (5)	**59**
15	10	6	NBu_4BF_4 (1.0)	2,4,6-Collidine (2.0)	Ph-S-Me (1.0)	$C_{gr.}$	Ni	-	CH_2Cl_2 (5)	**67**
16	5	6	NBu_4BF_4 (1.0)	2,4,6-Collidine (2.0)	Ph-S-Me (1.0)	$C_{gr.}$	Pt	-	CH_2Cl_2 (5)	**66**
17	20	6	NBu_4BF_4 (1.0)	2,4,6-Collidine (2.0)	Ph-S-Me (1.0)	$C_{gr.}$	Pt	-	CH_2Cl_2 (5)	**62**
18	10	6	NBu_4BF_4 (1.0)	2,4,6-Collidine (2.0)	Ph-S-Me (1.0)	$C_{gr.}$	Pt	0.8M	CH_2Cl_2 (5)	**62**
19	10	6	NBu_4BF_4 (1.0)	2,4,6-Collidine (2.0)	Ph-S-Et (1.0)	$C_{gr.}$	Pt	-	CH_2Cl_2 (5)	**31**

20	10	6	NBu_4BF_4 (1.0)	2,4,6-Collidine (2.0)	Me-S-Me (1.0)	$C_{gr.}$	Pt	-	CH_2Cl_2 (5)	**63**
21	10	8	NBu_4BF_4 (1.0)	2,4,6-Collidine (2.0)	Me-S-Me (1.0)	$C_{gr.}$	Pt	-	CH_2Cl_2 (5)	**61**
22	10	8	NBu_4BF_4 (0.5)	2,4,6-Collidine (2.0)	Ph-S-Me (1.0)	$C_{gr.}$	Pt	-	CH_2Cl_2 (5)	**62**
23	10	8	NBu_4BF_4 (0.3)	2,4,6-Collidine (2.0)	Ph-S-Me (1.0)	$C_{gr.}$	Pt	-	CH_2Cl_2 (5)	**65**
24	10	8	NBu_4BF_4 (0.1)	2,4,6-Collidine (2.0)	Ph-S-Me (1.0)	$C_{gr.}$	Pt	-	CH_2Cl_2 (5)	**64**
25	10	6	NBu_4BF_4 (1.0)	2,4,6-Collidine (1.0)	Ph-S-Me (1.0)	$C_{gr.}$	Pt	-	CH_2Cl_2 (5)	**59**
26	10	6	NBu_4BF_4 (1.0)	2,4,6-Collidine (3.0)	Ph-S-Me (1.0)	$C_{gr.}$	Pt	-	CH_2Cl_2 (5)	**67**
27	10	6	NBu_4BF_4 (1.0)	2,4,6-Collidine (2.0)	Ph-S-Me (1.5)	$C_{gr.}$	Pt	-	CH_2Cl_2 (5)	**69**
28	10	9	NBu_4BF_4 (1.0)	2,4,6-Collidine (2.0)	Ph-S-Me (1.5)	$C_{gr.}$	Pt	-	CH_2Cl_2 (5)	**51**
29	10	3	NBu_4BF_4 (1.0)	2,4,6-Collidine (2.0)	Ph-S-Me (0.5)	$C_{gr.}$	Pt	-	CH_2Cl_2 (5)	**55**
30	10	6	NBu_4BF_4 (1.0)	2,4,6-Collidine (2.0)	Ph-S-Me (0.5)	$C_{gr.}$	Pt	-	CH_2Cl_2 (5)	**49**
31	10	6	NBu_4BF_4 (1.0)	2,4,6-Collidine (2.0)	-	$C_{gr.}$	Ni	-	CH_2Cl_2 (5)	**14**

32	10	6	NBu_4BF_4 (1.0)	-	Ph-S-Me (1.0)	$C_{gr.}$	Ni	-	CH_2Cl_2 (5)	**n.d.**
33	10	0	NBu_4BF_4 (1.0)	2,4,6-Collidine (2.0)	Ph-S-Me (1.0)	$C_{gr.}$	Ni	-	CH_2Cl_2 (5)	**n.d.**
34	10	6	NBu_4BF_4 (1.0)	2,4,6-Collidine (2.0)	Ph-S-Me (1.0)	$C_{gr.}$	Ni	No 3Å-MS	CH_2Cl_2 (5)	**70**
35	10	6	NBu_4BF_4 (1.0)	2,4,6-Collidine (2.0)	Ph-S-Me (1.0)	$C_{gr.}$	Pt	-	Acetone (5)	**7**
36	10	6	NBu_4BF_4 (1.0)	2,4,6-Collidine (2.0)	Ph-S-Me (1.0)	$C_{gr.}$	Pt	HFIP (2.0)	CH_2Cl_2 (5)	**91**
37	10	6	NBu_4BF_4 (1.0)	2,4,6-Collidine (2.0)	Ph-S-Me (1.0)	$C_{gr.}$	SS	HFIP (2.0)	CH_2Cl_2 (5)	**87**
38	10	6	NBu_4BF_4 (1.0)	2,4,6-Collidine (2.0)	Me-S-Me (1.0)	$C_{gr.}$	SS	HFIP (2.0)	CH_2Cl_2 (5)	92
39	10	6	NBu_4BF_4 (1.0)	2,4,6-Collidine (2.0)	Me-S-Me (0.5)	$C_{gr.}$	SS	HFIP (2.0)	CH_2Cl_2 (5)	**59**
40	10	6	NBu_4BF_4 (1.0)	2,4,6-Collidine (2.0)	Me-S-Me (1.0)	$C_{gr.}$	SS	HFIP (1.0)	CH_2Cl_2 (5)	**89**
41	10	6	NBu_4BF_4 (1.0)	2,4,6-Collidine (2.0)	Me-S-Me (1.0)	$C_{gr.}$	SS	HFIP (4.0)	CH_2Cl_2 (5)	**59**
42	10	6	NBu_4BF_4 (1.0)	2,4,6-Collidine (2.0)	Me-S-Me (1.0)	$C_{gr.}$	SS	HFIP (6.0)	CH_2Cl_2 (5)	**40**
43	10	6	NBu_4BF_4 (1.0)	2,4,6-Collidine (2.0)	-	$C_{gr.}$	SS	HFIP (2.0)	CH_2Cl_2 (5)	**7**

44	10	6	NBu_4BF_4 (1.0)	-	Me-S-Me (1.0)	$C_{gr.}$	SS	HFIP (2.0)	CH_2Cl_2 (5)	**15**
45	10	6	NBu_4BF_4 (1.0)	2,4,6-Collidine (2.0)	Me-S-Me (1.0)	$C_{gr.}$	SS	-	CH_2Cl_2 (5)	**73**
46	10	0	NBu_4BF_4 (1.0)	2,4,6-Collidine (2.0)	Me-S-Me (1.0)	$C_{gr.}$	SS	HFIP (2.0)	CH_2Cl_2 (5)	**0**

2.2: Flow Optimisation Studies

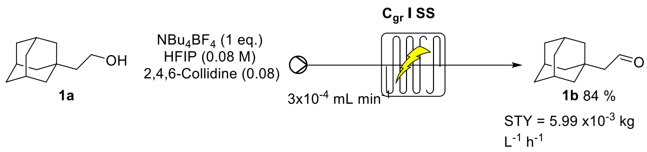

Scheme S2. Optimizezd reaction conditions for the continuous flow eSwern reaction (Entry 5)

Table S2: Full optimisation table for the continuous flow eSwern reaction. Yields were calculated via GC-MS using a calibration curve with n-decane and ethyl phenyl acetate as internal standards. *Reaction was run for an hour to investigate the steady state performance of the system. No decrease in productivity or change in voltage was observed and inspection of the cell post reaction showed no deposition of products on either electrode.

Entry	Current [mA]	Eq. Electrons [F/mol]	Flow Rate (uL/min)	Concentration (M)	Anode	Cathode	Yield of 1b (%)
1	300	6.06	770	0.04	$C_{gr.}$	$SS_{r.}$	59
2	350	5.5	1000	0.04	$C_{gr.}$	SS	34
3	900	7	2000	0.04	$C_{gr.}$	SS	24
4	193	6	500	0.04	$C_{gr.}$	SS	64
5*	116	6.01	300	0.04	$C_{gr.}$	SS	84
6	600	0.93	1000	0.4	$C_{gr.}$	SS	26
7	1000	1.55	1000	0.4	$C_{gr.}$	SS	44
8	1200	1.87	1000	0.4	$C_{gr.}$	SS	41
9	1400	2.18	1000	0.4	$C_{gr.}$	SS	62
10	1600	2.48	1000	0.4	$C_{gr.}$	SS	56
11	2000	3.11	1000	0.4	$C_{gr.}$	SS	34
12	1400	2.72	800	0.4	$C_{gr.}$	SS	54

13	708	2.2	500	0.4	$C_{gr.}$	SS	69
14	2830	2.2	2000	0.4	$C_{gr.}$	SS	59
15	1500	2.33	1000	0.4	$C_{gr.}$	SS	64

3. Experimental Procedures

3.1: Synthesis of Starting Materials

General procedure A for synthesis of alcohols from R-benzyl bromide

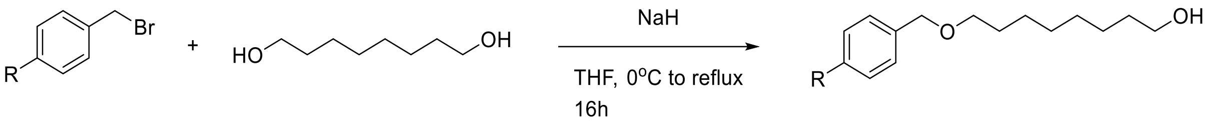

Scheme S3. General reaction conditions for the alkylation of substituted benzyl bromides with octane-1,8-diol

To a 3-necked 100 mL round bottomed flask was added NaH (0.4000 g, 10 mmol, 60% in paraffin oil) was added before the flask was purged with nitrogen. A solution of octane-1,8-diol (2.0548 g, 10 mmol) in THF (50 mL) was added and stirred for 30 minutes. Then benzyl bromide (g and mmol. as specified for each substrate) in THF (5 mL) was added dropwise, and the reaction mixture was brought to reflux and stirred for 16 hours. The reaction was quenched with deionised water (30 mL) before being extracted with ethyl acetate (3x40 mL). The combined organic layers were washed with deionised water (3x50 mL) and dried over anhydrous Na_2SO_4 and solvent was removed under reduced pressure. Purification by flash column chromatography (100 % petroleum ether to 20 % EtOAc in petroleum ether) afforded the desired product.

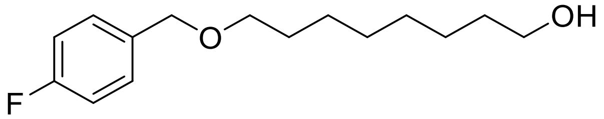

Synthesised according to procedure A with 1-(bromomethyl)-4-fluorobenzene (1.8902 g, 10 mmol). Extraction with ethyl acetate and purification by flash column chromatography (100 % petroleum ether to 20 % EtOAc in petroleum ether) yielded the product as a colourless oil (1.1734 g, 52 %). **SM01:** 1H NMR (500 MHz, $CDCl_3$): δH 7.28 (dd, *J* = 8.5, 5.6 Hz, 2H), 7.05 – 6.96 (m, 2H), 4.44 (s, 2H), 3.60 (t, *J* = 6.7 Hz, 2H), 3.43 (t, *J* = 6.6 Hz, 2H), 1.74 (s, 1H), 1.62 – 1.49 (m, 4H), 1.36 – 1.25 (m, 8H). 13C{1H} NMR (126 MHz, $CDCl_3$): δ 163.32-161.37 (d, J = 246 Hz), 134.48, 129.45 (d, J = 8.1 Hz), 115.35-115.18 (d, J = 21.3 Hz), 72.25, 70.58, 63.05, 32.83, 29.79, 29.50, 29,45, 26.20, 25.77. HRMS (ESI): m/z calcd for $C_{15}H_{24}FO_2^+$ 255.1760 $[M+H]^+$ found 255.1751 IR (cm^{-1}): 3350, 2929, 2856.

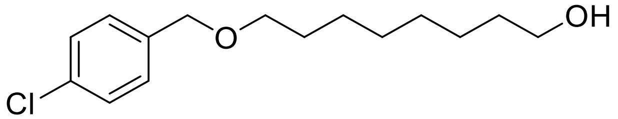

Synthesised according to procedure A with 1-(bromomethyl)-4-chlorobenzene (2.0548 g, 10 mmol.). Extraction with ethyl acetate and purification by flash column chromatography (100% petroleum Ether to 20% EtOAc in petroleum ether) gave a colourless oil (1.0381 g, 41 %). **SM02:** 1H NMR (500 MHz, $CDCl_3$): δH 7.34 – 7.23 (m, 4H), 4.45 (s, 2H), 3.62 (t, *J* = 6.6 Hz, 2H), 3.44 (t, *J* = 6.6 Hz, 2H), 1.65 – 1.50 (m, 4H), 1.36 – 1.28 (m, 8H). 13C{1H} NMR (126 MHz, $CDCl_3$): δ 137.31, 133.30, 129.04, 128.61, 72.20, 70.71, 63.16, 32.88, 29.83, 29.53, 29.48, 26.24, 25.80. HRMS (ESI): m/z calcd for $C_{15}H_{22}ClO_2^+$ 269.1308 $[M+H]^+$ found 269.1305 IR (cm^{-1}): 3368, 2929, 2855.

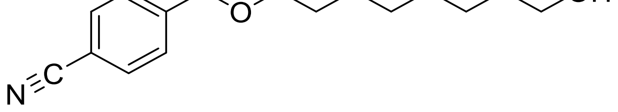

Synthesised according to procedure A with 4-(bromomethyl)benzonitrile (1.9605g, 10 mmol.). Extraction with ethyl acetate and purification by flash column chromatography (100 % petroleum ether to 20 % EtOAc in petroleum ether) gave a pale yellow oil (0.9848 g, 38 %). **SM03:** 1H NMR (400 MHz, $CDCl_3$): δH 7.62 (d, *J* = 8.3 Hz, 2H), 7.43 (d, *J* = 8.0 Hz, 2H), 4.53 (s, 2H), 3.63 (t, *J* = 6.6 Hz, 2H), 3.48 (t, *J* = 6.6 Hz, 2H), 1.64 – 1.52 (m, 4H), 1.34 (m, 8H). 13C{1H} NMR (100 MHz, $CDCl_3$): δ 144.45, 132.27, 127.72, 118.98, 111.26, 71.95, 71.17, 63.11, 32.83, 29.75, 29.47, 29.42, 26.17, 25.76. HRMS (ESI): m/z calcd for $C_{16}H_{24}NO_2^+$ 262.1807 $[M+H]^+$ found 262.1805 IR (cm^{-1}): 3342, 2931, 2855, 2226.

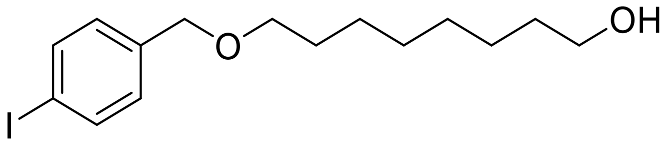

Synthesised according to procedure A with 4-iodobenzyl bromide (2.9693 g, 10 mmol). Extraction with ethyl acetate and purification by flash column chromatography (100 % petroleum ether to 20 % EtOAc in petroleum ether) to give an off while powder (2.2532 g, 62 %). **SM04:** 1H NMR (500 MHz, $CDCl_3$): δH 7.70 – 7.62 (m, 2H), 7.11 – 7.05 (m, 2H), 4.42 (s, 2H), 3.62 (t, *J* = 6.8 Hz, 2H), 3.43 (t, *J* = 6.6 Hz, 2H), 1.61 – 1.52 (m, 4H), 1.35 – 1.29 (m, 8H). 13C{1H} NMR (126 MHz, $CDCl_3$): δ 138.53, 137.54, 129.61, 92.99, 72.29, 70.73, 63.18, 32.89, 29.83, 29.53, 29.48, 26.23, 25.80. HRMS (ESI): m/z calcd for $C_{15}H_{24}O_2I^+$ 363.0821 $[M+H]^+$ found 363.0818 IR (cm^{-1}): 3330, 2930, 2848. Melting point: 58-59 °C. HR

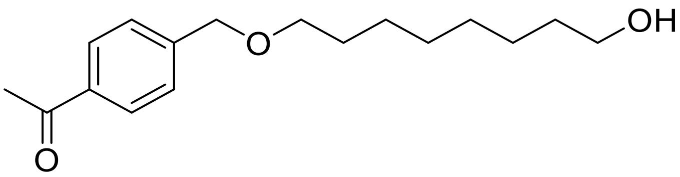

Synthesised according to procedure A with 1-(4-(bromomethyl)phenyl)ethan-1-one (1.000 g, 4.69 mmol.), octane-1,8-diol (0.6858 g, 4.69 mmol.) and NaH (0.1126 g, 4.69 mmol., 60 % dispersion in paraffin oil). Extraction with ethyl acetate and purification with flash column chromatography(100 % petroleum ether to 20 % EtOAc in petroleum ether) to give a white powder (0.23 g, 18 %). **SM05:** 1H NMR (500 MHz, $CDCl_3$): δH 7.93 (d, J = 8.3 Hz, 2H), 7.47 – 7.38 (m, 2H), 4.54 (s, 2H), 3.62 (t, *J* = 6.6 Hz, 2H), 3.47 (t, *J* = 6.6 Hz, 2H), 2.59 (s, 3H), 1.64 – 1.52 (m, 4H), 1.44 (s, 1H), 1.38 – 1.29 (m, 8H). 13C{1H} NMR (126 MHz, $CDCl_3$): δ 198.05,144.45, 136.43 128.61, 127.43, 72.31, 70.99, 63.16, 32.89, 29.84, 29.53, 29.48, 26.79, 26.24, 25.81. HRMS (ESI): m/z calcd for $C_{17}H_{27}O_3^+$ 279.1960 $[M+H]^+$ found 279.1960 IR (cm^{-1}): 4326, 2931, 2850, 1669. Melting point: 53 – 56 °C.

Synthesis of 8-((tert-butyldiphenylsilyl)oxy)octan-1-ol

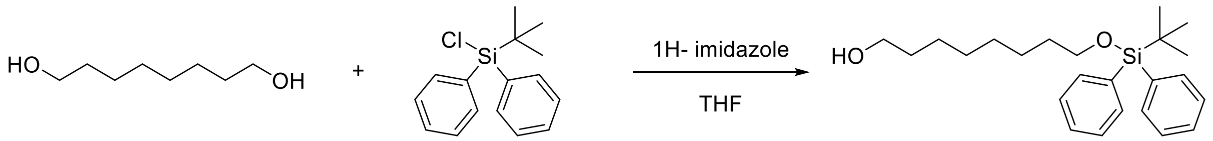

Scheme S4. Reaction conditions for the synthesis of 8-((tert-butyldiphenylsilyl)oxy)octan-1-ol

Under argon, octane-1,8-diol (2.9246 g, 20 mmol.) and 1H-imidazole (0.6808 g, 10 mmol.) were stirred in THF (20mL) for 30 minutes before tertbutylchlorodiphenylsilane (2.7486 g, 10 mmol.) was added dropwise. The reaction mixture was stirred for 72 hours before being quenched with aqueous NH_4Cl solution (20 mL). The organic phase was extracted with EtOAc (3x 20 mL) and washed with saturated aqueous NH_4Cl solution (3x 50 mL) and dried over anhydrous Na_2SO_4 before solvent was removed under reduced pressure. Purification by flash column chromatography (100 % petroleum ether to 40 % EtOAc in petroleum ether) gave a pale yellow oil (1.7496 g, 46 %). **SM06:** 1H NMR (400 MHz, $CDCl_3$): δH 7.68 (dd, *J* = 7.8, 1.7 Hz, 4H), 7.47 – 7.31 (m, 6H), 3.65 (dt, *J* = 13.5, 6.6 Hz, 4H), 1.60 – 1.53 (m, 4H), 1.44 (s, 1H), 1.37 – 1.26 (m, 8H), 1.06 (s, 9H). 13C{1H} NMR (100 MHz, $CDCl_3$): δ 135.68, 134.29, 129.58, 127.66, 64.08, 63.14, 32.88, 32.65, 29.48, 29.42, 26.99, 25.81, 25.78, 19.33. HRMS (ESI): m/z calcd for $C24H37O_2Si^+$ 385.2557 $[M+H]^+$ found 385.2555 IR (cm^{-1}) 3334, 2929, 2856.

Synthesis of 12-iodo-dodecan-1-ol

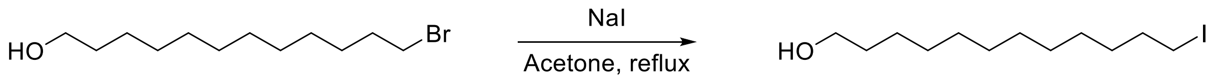

Scheme S5. Reaction conditions for the synthesis of 12-iodo-doddecan-1-ol

Sodium iodide (0.7453 g, 5 mmol.) was added to a solution of bromododecanol (1.3256 g, 5 mmol.) in acetone (20 mL) and refluxed in darkness for 72 hours. The mixture was filtered and remaining salts were washed with acetone. The combined organic layers were concentrated to give a yellow solid (1.5602 g, 99%). 1H NMR (400 MHz, $CDCl_3$): δH 3.63 (td, *J* = 6.6, 5.3 Hz, 2H), 3.18 (t, *J* = 7.0 Hz, 2H), 1.85 – 1.76 (m, 2H), 1.61 – 1.52 (m, 2H), 1.30-1.18 (m, 16H), 1.20 (t, *J* = 5.4 Hz, 1H).13C{1H}

NMR (100 MHz, $CDCl_3$): δ 63.18, 33.65, 32.89, 30.58, 29.65, 29.62, 29.59, 29.49, 29.47, 28.61, 25.81, 7.40. 1H and 13C NMR in accordance with the literature.[1]

Synthesis of 12-azido-dodecan-1-ol

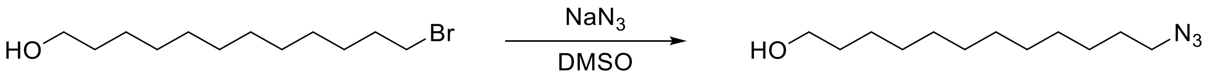

Scheme S6. Reaction Conditions for the synthesis of 12-azido-dodecan-1-ol

Sodium azide (0.3200 g, 4.95 mmol.) was stirred in DMSO (10.3 mL) for 12 hours to form a homomgenous suspension before 12-bromododecan-1-ol (1.071 g, 4.5 mmol.) was added. The mixture was stirred at room temperature for 24 hours before being quenched with cooled distilled water (20mL) and stirred to room temperature. Product was extracted with DCM (3x 20 mL) and washed with Brine (3 x 10 mL). Purified by flash column chromatography (100 % petroleum ether to 20 % EtOAc in petroleum ether) to yield a colourless oil (0.6889 g, 67 %). **SM08:** 1H NMR (400 MHz, $CDCl_3$): δH 3.64-3.61 (m, 2H), 3.24 (t, *J* = 7.0 Hz, 2H), 2.60 (s, 1H), 1.63 – 1.50 (m, 4H), 1.38 – 1.25 (m, 16H). 13C{1H} NMR (100 MHz, $CDCl_3$): δ 63.15, 51.57, 32.88, 29.65, 29.60, 29.58, 29.53, 29.49, 29.21, 28.91, 26.79, 25.81. HRMS (ESI): m/z calcd for $C_{12}H_{29}N_4O^+$ 245.3341 $[M+NH_4]^+$ found 245.2335. IR (cm^{-1}): 3339, 2924, 2853, 2092.

Synthesis of 12-(ethylthio)dodecan-1-ol

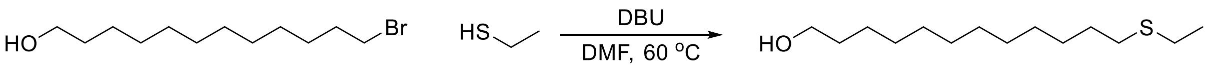

Scheme S7. Reaction conditions for the synthesis of 12-(ethylthio)dodecan-1-ol

Ethanethiol (1.5 mL, 17 mmol.) was added to anhydrous DMF and degassed with argon. 1,8-Diazobicyclo[5.4.0]undec-7-ene) (2.54 mL, 17mmol) was added and the mixture was stirred for 20 minutes. 12-bromododecan-1-ol (2.2500 g, 8.5 mmol.) was dissolved in DMF (15 mL) and the solution was added to the reaction mixture dropwise. Upon addition of 12-bromododecan-1-ol the solution turned from colourless to a pale pink colour. The mixture was swas heated to 60°C and a stirred for 18 hours. A colour change to crimson red was noted. Upon observation of full conversion of the starting material via GC-MS the mixture was diluted with EtOAc (400 mL) before being washed with distilled water (2x 400 mL) and saturated aqueous NH_4Cl solution (2x 400 mL) before being dried over anhydrous sodium sulfate. Purification by flash column chromatography (100 % petroleum ether to 20 % in petroleum ether) gave a waxy white solid (1.6600 g, 84%). 1H NMR (400 MHz, $CDCl_3$): δH 3.63 (t, *J* = 6.6 Hz, 2H), 2.57 – 2.45 (m, 4H), 1.60 – 1.53 (m, 4H), 1.36 – 1.23 (m, 20H). 13C{1H} NMR (100 MHz, $CDCl_3$): δ 63.18, 32.90, 31.78, 29.74, 29.66, 29.64, 29.63, 29.59, 29.49, 29.33, 29.04, 26.01, 25.81, 14.90. IR (cm^{-1}): 3334, 2918, 2848. HRMS (ESI): m/z calcd for $C_{14}H_{29}OS^+$ 245.1939 $[M+H]^+$ found 245.1936. Melting point: 48 - 50 °C.

Synthesis of 12-(ethylsulfonyl)dodecan-1-ol

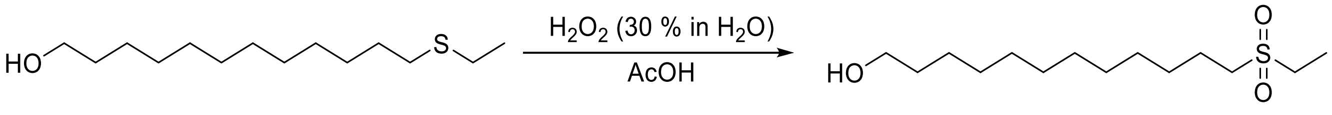

Scheme S8. Reaction conditions for the synthesis of 12-(ethylsulfonyl)dodecan-1-ol

12-(ethylthio)dodecan-1-ol (1g, 4mmol.) was stirred in acetic acid (30 mL) and H_2O_2 (30 mL, 30 % in H_2O) for 72 hours. The product was extracted with EtOAc (2x 50mL) before being washed with deionised water (3x 50mL) and dried over anhydrous sodium sulfate. Solvent was removed under reduced pressure to yield a white powder (1.1126 g, quantitative). 1H NMR (400 MHz, $CDCl_3$): δH 3.63 (t, *J* = 6.6 Hz, 2H), 3.00 – 2.92 (m, 4H), 1.86 – 1.79 (m, 2H), 1.57 – 1.53 (m, 4H), 1.44 – 1.37 (m, 4H), 1.35 – 1.23 (m, 14H). 13C{1H} NMR (100 MHz, $CDCl_3$): δ 63.16, 52.02, 47.07, 32.88, 29.60, 29.53, 29.51, 29.46, 29.28, 29.11, 28.59, 25.79, 21.98, 6.73. IR (cm^{-1}) 3302, 2914, 2848, 1737. HRMS (ESI): m/z calcd for $C_{14}H_{31}OS^+$ 279.1994 $[M+H]^+$ found 2479.2000. Melting Point: 55 – 58 °C.

3.2: General Procedure B for the electrochemical oxidation of alcohols to aldehydes/ketones.

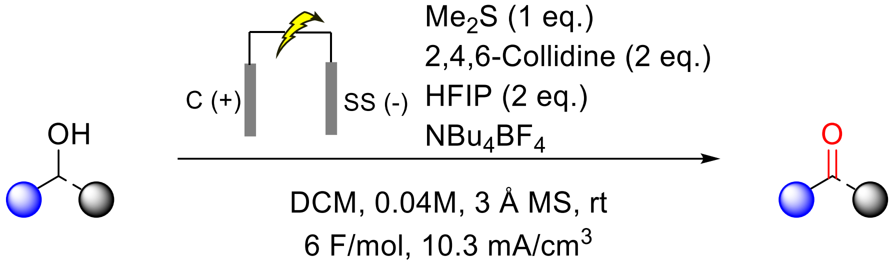

Scheme S9. General Reaction conditions for the eSwern Reaction

To a 5 mL IKA Electrasyn vial was added alcohol (0.2 mmol), 3-Å molecular sieves (0.1500 g), NBu_4BF_4 (0.0659 g, 0.2 mmol.), DCM (5 mL), 1,1,1,3,3,3-hexafluoro-2-propanol (0.042 mL, 0.4 mmol), 2,4,6-trimethyl pyridine (0.053 mL, 0.4 mmol) and (methylsulfanyl)methane (0.015 mL, 0.2 mmol). The resulting mixture was electrolysed at a constant current of 10 mA while stirring at 600 rpm for 6 F/mol with a carbon anode and stainless steel cathode. The solution was transferred to a separating funnel and the vial and electrodes were washed with DCM (5 mL) which was transferred to the separating funnel. The reaction mixture was diluted with diethyl ether (20 mL) and hexanes (20 mL) before being washed with 1 M HCl (3x40 mL) and deionised water (3x 40 mL). The organic layer was dried over anhydrous Na_2SO_4, filtered and solvent was removed under reduced pressure to afford the desired aldehyde/ketone.

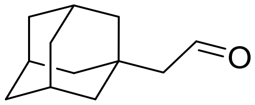

1a Synthesised according to procedure B with 1-adamantaneethanol (0.0361 g, 0.2 mmol) to yield **1a** as a colourless oil (0.0332 g, 93%). 1H NMR (400 MHz, $CDCl_3$): δH 9.86 (t, *J* = 3.3 Hz, 1H), 2.11 (d, *J* = 3.3 Hz, 2H), 1.99 – 1.95 (m, 3H), 1.74 – 1.63 (m, 12H). 13C{1H} NMR (100 MHz, $CDCl_3$): δ 203.87, 57.36, 42.84, 36.74, 33.45, 28.57. HRMS (ESI): m/z calcd for $C_{12}H_{19}O^+$ 179.1436 $[M+H]^+$ found 179.1436. IR (cm^{-1}): 2899, 2847, 1719 (C=O stretch).

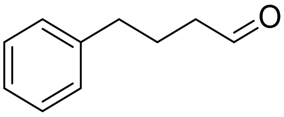

1b Synthesised according to procedure B with 4-phenylbutan-1-ol (0.0300 g, 0.2 mmol) to yield **1b** as a colourless oil (0.029 g, 98 %). 1H NMR (400 MHz, DMSO-d_6): δH 9.75 (t, *J* = 1.6 Hz, 1H), 7.31 – 7.24 (m, 2H), 7.22 – 7.13 (m, 3H), 2.66 (t, *J* = 7.6 Hz, 2H), 2.45 (td, *J* = 7.3, 1.7 Hz, 2H), 2.03 – 1.91 (m, 2H). 13C{1H} NMR (100 MHz, DMSO-d_6): δ 203.84, 142.05, 128.85, 128.77, 126.39, 42.97, 34.91, 23.98. Spectral data in accordance with the literature.[2]

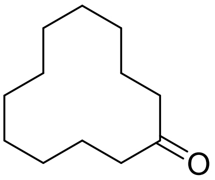

1c Synthesised according to procedure B with cyclododecanol (0.0369 g, 0.2 mmol) to yield **1c** as a colourless oil (0.0335 g, 92 %). 1H NMR (400 MHz, $CDCl_3$): δH 2.51 – 2.42 (m, 4H), 1.76 – 1.66 (m, 4H), 1.56 (H_2O), 1.33 – 1.26 (m, 14H). ^{1}H NMR spectrum in accordance with the literature.[3]

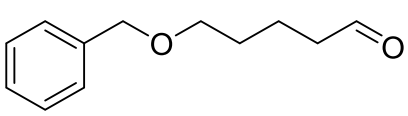

1d Synthesised according to procedure B with 5-(benzyloxy)pentan-1-ol (0.0389 g mg, 0.2 mmol) to give **1d** as a colourless oil (0.0354 g, 92 %). 1H NMR (400 MHz, DMSO-d_6): δH 9.65 (t, *J* = 1.6 Hz, 1H), 7.34 – 7.20 (m, 5H), 4.40 (s, 2H), 3.38 (t, *J* = 6.1 Hz, 2H), 2.40 (td, *J* = 7.0, 1.5 Hz, 2H), 1.56 –1.51 (m, 4H). 13C{1H} NMR (100 MHz, DMSO-d_6): 203.91, 139.20, 128.75, 127.92, 72.33, 69.78, 43.24, 29.11, 18.99. ^{1}H and ^{13}C spectra in accordance with the literature.[4]

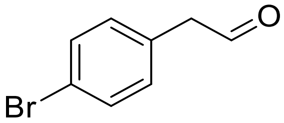

1e Synthesised according to procedure B with 2-(4-bromophenyl)ethan-1-ol (0.0402 g, 0.2 mmol) to yield **1e** a brown oil (0.0354 g, 89 %). 1H NMR (500 MHz, $CDCl_3$): δH 9.73 (t, *J* = 2.1 Hz, 1H), 7.49 (d, *J* = 8.3 Hz, 2H), 7.09 (d, *J* = 8.3 Hz, 2H), 3.66 (d, *J* = 2.2 Hz, 2H). 13C{1H} NMR (126 MHz, $CDCl_3$): δ 198.74, 132.17, 131.42, 130.88, 121.62, 49.94. ^{1}H and ^{13}C spectra in accordance with the literature.[5]

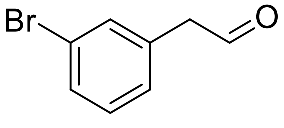

1f Synthesised according to procedure B with 2-(3-bromophenyl)ethan-1-ol (0.0402 g, 0.2 mmol) to yield **1f** as a pale yellow oil. (0.0311 g, 78%). 1H NMR (400 MHz, $CDCl_3$): δH 9.75 (t, *J* = 2.1 Hz, 1H), 7.48 – 7.42 (m, 1H), 7.39 (t, *J* = 1.9 Hz, 1H), 7.24 (t, *J* = 7.8 Hz, 1H), 7.17 – 7.13 (m, 1H), 3.68 (d, *J* = 2.2 Hz, 2H). 13C{1H} NMR (100 MHz, $CDCl_3$): δ 198.46, 134.12, 132.72, 130.69, 130.55, 128.34, 123.06, 50.03. 1H and 13C NMR spectra in accordance with the literature.[6]

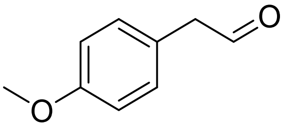

1g Synthesised according to procedure B with 2-(4-methoxyphenyl)ethan-1-ol (0.0304 g, 0.2 mmol) to yield a brown oil (0.0288 g, 96%). 1H NMR (500 MHz, $CDCl_3$): δH 9.71 (t, *J* = 2.4 Hz, 1H), 7.13 (d, *J* = 8.6 Hz, 2H), 6.91 (d, *J* = 8.6 Hz, 2H), 3.80 (s, 3H), 3.63 (d, *J* = 2.4 Hz, 2H). 13C{1H} NMR (126 MHz, $CDCl_3$): δ 200.00, 159.05, 130.79, 123.76, 114.54, 55.38, 49.79. ^{1}H and ^{13}C NMR spectra in accordance with the literature.[7]

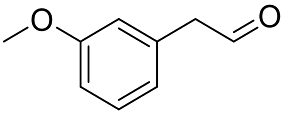

1h Synthesised according to procedure B with 2-(3-methoxyphenyl)ethan-1-ol (0.0304 g, 0.2 mmol) to yield **1h** as a yellow oil (0.027 g, 90 %). 1H NMR (500 MHz, $CDCl_3$): δH 9.73 (t, *J* = 2.4 Hz, 1H), 7.12 – 7.05 (m, 1H), 6.66 – 6.55 (m, 3H), 3.61 (s, 3H), 3.46 (d, *J* = 2.4 Hz, 2H). 13C{1H} NMR (126 MHz, $CDCl_3$): δ 199.59, 160.15, 133.34, 130.14, 122.00, 115.36, 112.95, 55.32, 50.67. ^{1}H and ^{13}C NMR spectra in accordance with the literature.[8]

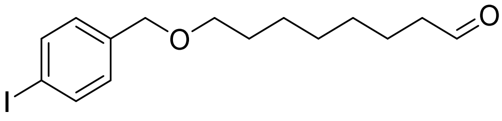

1i Synthesised according to procedure B with 8-((4-iodobenzyl)oxy)octan-1-ol (0.0724 g, 0.2 mmol) to yield **1i** as a brown oil (0.0585 g, 81 %). 1H NMR (500 MHz, $CDCl_3$): δH 9.74 (t, *J* = 1.8 Hz, 1H), 7.65 (d, *J* = 8.2 Hz, 2H), 7.06 (d, *J* = 8.2 Hz, 2H), 4.41 (s, 2H), 3.42 (t, *J* = 6.6 Hz, 2H), 2.40 (td, *J* = 7.3, 1.8 Hz, 2H), 1.63 – 1.54 (m, 4H), 1.34 – 1.28 (m, 8H). 13C{1H} NMR (126 MHz, $CDCl_3$): δ 203.03, 137.50, 129.58, 92.98, 72.25, 70.58, 43.97, 29.73, 29.26, 29.17, 29.17, 26.06, 22.08. HRMS (ESI): m/z calcd for $C_{15}H_{22}O_2^{+}$ 361.0664 [M+H^{+}] found 361.0663.

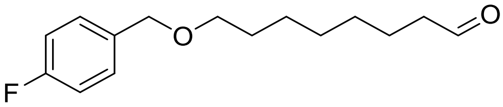

1j Synthesised according to procedure B with 8-((4-fluorobenzyl)oxy)octan-1-ol (0.0508 g, 0.2 mmol) to yield **1j** as a colourless oil (0.0409 g, 81 %). 1H NMR (500 MHz, $CDCl_3$): δH 9.74 (t, *J* = 1.8 Hz, 1H), 7.28 (dd, *J* = 8.4, 5.6 Hz, 2H), 7.00 (t, *J* = 8.7 Hz, 2H), 4.43 (s, 2H), 3.43 (t, *J* = 6.6 Hz, 2H), 2.40 (td, *J* = 7.3, 1.9 Hz, 2H), 1.63 – 1.55 (m, 4H), 1.38 – 1.26 (m, 8H). 13C{1H} NMR (100 MHz, $CDCl_3$): δ 202.94, 163.56-161.12 (d, J= 975 Hz), 134.47 (d, *J* = 3.1 Hz), 129.44 (d, J = 8.0 Hz), 115.35 (d, J = 21 Hz) 72.24, 70.46, 43.93, 29.71, 29.23, 29.15, 26.05, 22.06. HRMS (ESI): m/z calcd for $C_{15}H_{22}FO_2^+$ 253.1598 [M+H$^+$] found 253.1524. IR (cm^{-1}) 2717, 1721.

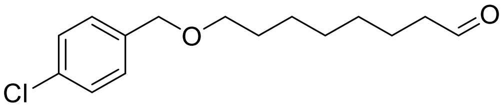

1k Synthesised according to procedure B with 8-4((4-chlorobenzyl)oxy)octan-1-ol (0.0541 g, 0.2 mmol.) to yield a brown oil. NMR yield (92 %) calculated using dibromomethane (0.0353 g, 0.2 mmol) as an internal standard. Purification by flash column chomotography (100% petroleum ether to 20% EtOAc in petroleum ether) yielded **1k** as a colourless oil (0.0122 g, 23 %). 1H NMR (500 MHz, $CDCl_3$): δH 9.75 (t, *J* = 1.8 Hz, 1H), 7.30 (d, *J* = 8.4 Hz, 2H), 7.25 (d, *J* = 2.9 Hz, 2H), 4.44 (s, 2H), 3.43 (t, *J* = 6.5 Hz, 2H), 2.41 (td, *J* = 7.4, 1.8 Hz, 2H), 1.63 – 1.57 (m, 4H), 1.39 – 1.24 (m, 8H). 13C{1H} NMR (100 MHz, $CDCl_3$): δ 203.03, 137.28, 129.04, 128.63, 72.22, 70.61, 44.02, 29.78, 29.31, 29.21, 26.12, 22.12. HRMS (ESI): m/z calcd for $C_{15}H_{22}ClO_2^+$ 253.1303 [M+H$^+$] found 269.1296. IR (cm^{-1}) 2715, 1725.

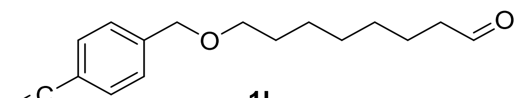

1l Synthesised according to procedure B with 8-((4-4-(((8-hydroxyoctyl)oxy)methyl)benzonitrile (0.0522 g, 0.2 mmol) to yield a brown oil. NMR yield (82 %) was calculated using dibromomethane (0.033 g, 0.19 mmol) as an internal standard. Purification by flash column chomotography (100% petroleum ether to 20% EtOAc in petroleum ether) yielded **1l** a colourless oil (0.0156, 30 %). 1H NMR (500 MHz, $CDCl_3$): δH 9.76 (t, *J* = 1.8 Hz, 1H), 7.63 (d, *J* = 8.3 Hz, 2H), 7.44 (d, *J* = 8.4 Hz, 2H), 4.54 (s, 2H), 3.48 (t, *J* = 6.5 Hz, 2H), 2.43 (td, *J* = 7.3, 1.8 Hz, 2H), 1.65 – 1.60 (m, 4H), 1.38 – 1.24 (m, 8H). 13C{1H} NMR (126 MHz, $CDCl_3$): δ 202.97, 144.44, 132.33, 127.77, 119.05, 111.27, 72.00, 71.12, 44.00, 29.74, 29.31, 29.19, 26.10, 22.09. IR (cm^{-1}) 2720, 1722. No mass peak corresponding to the product found via HRMS.

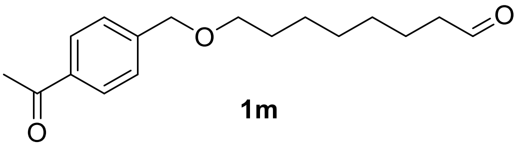

1m Synthesised according to procedure B with1-(4-(((8-hydroxyoctyl)oxy)methyl)phenyl)ethan-1-one (0.0557 g, 0.2 mmol.) to yield a brown oil. NMR yield (63 %) was calculated using dibromomethane as an internal standard (0.0303 g, 0.17 mmol.). Purification by flash column chomotography (100% petroleum ether to 20% EtOAc in petroleum ether) yielded a white powder (0.0083, 15 %). 1H NMR (500 MHz, $CDCl_3$): δH 9.76 (t, *J* = 1.8 Hz, 1H), 7.96 – 7.92 (m, 2H), 7.43 (dt, *J* = 8.0, 0.7 Hz, 2H), 4.55 (s, 2H), 3.48 (t, *J* = 6.5 Hz, 2H), 2.60 (s, 3H), 2.42 (td, *J* = 7.3, 1.8 Hz, 2H), 1.65 – 1.60 (m, 4H), 1.42 – 1.25 (m, 8H). 13C{1H} NMR (100 MHz, $CDCl_3$): δ 202.89, 197.94, 144.35, 136.43, 128.56, 127.37, 72.28, 70.86, 43.95, 29.73, 29.25, 29.16, 26.72, 26.07, 22.07. IR (cm^{-1}) 2720, 1722, 1683. Melting point: 51-53 °C. . No mass peak corresponding to the product found via HRMS.

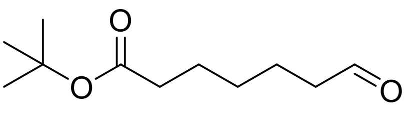

1n Synthesised according to procedure B with tert-butyl 7-hydroxyheptanoate (0.0405 g, 0.2 mmol.) to yield **1n** as a colourless oil (0.0368 g, 92 %). 1H NMR (500 MHz, $CDCl_3$): δH 9.65 (t, *J* = 1.7 Hz, 1H), 2.33 (td, *J* = 7.4, 1.8 Hz, 2H), 2.11 (t, *J* = 7.4 Hz, 2H), 1.56 – 1.46 (m, 4H), 1.33 (s, 9H), 1.24 (m, 2H). 13C{1H} NMR (126 MHz, $CDCl_3$): δ 202.78, 173.19, 80.30, 43.80, 35.40, 28.65, 28.22, 24.88, 21.84. 1H and 13C NMR are in accordance with the literature.[9]

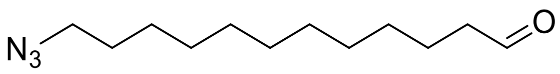

1o

Synthesised according to procedure B with 12-azido-dodecan-1-ol (0.0455, 0.2 mmol.) to yield **1o** as a colourless oil (0.0410 g, 91%). %). 1H NMR (500 MHz, $CDCl_3$): δH 9.68 (t, J = 1.8 Hz, 1H), 3.17 (t, *J* = 7.0 Hz, 2H), 2.34 (td, *J* = 7.4, 2.0 Hz, 2H), 1.56 – 1.48 (m, 4H), 1.25 – 1.18 (m, 14H). 13C{1H} NMR (126 MHz, $CDCl_3$): δ 203.13, 51.60, 44.04, 29.90, 29.55, 29.49, 29.45, 29.26 (x2), 28.95, 26.82, 22.18. ^{1}H and ^{13}C NMR in accordance with the literature.[10]

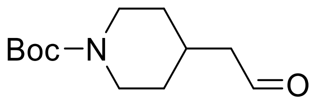

1p

Synthesised according to procedure B with tert-butyl 4-(2-hydroxyethyl)piperidine-1-carboxylate (0.0431 g, 0.2 mmol.) to yield **1p** as a pale yellow oil (0.0427 g, 94 %). 1H NMR (500 MHz, $CDCl_3$): δH 9.61 (t, *J* = 1.8 Hz, 1H), 3.91 (s, 2H), 2.57 (s, 2H), 2.22 (dd, *J* = 6.7, 1.8 Hz, 2H), 1.88 (ddd, *J* = 11.4, 7.8, 4.6 Hz, 1H), 1.55 – 1.50 (m, 2H), 1.28 (s, 9H), 1.02 – 0.98 (m, 2H). 13C{1H} NMR (126 MHz, $CDCl_3$): δ 201.73, 154.91, 79.61, 50.48, 43.59, 32.00, 30.75, 28.54. HRMS (ESI): m/z calcd for $C_{12}H_{22}O_3^+$ 228.1600 $[M+H]^+$ found 228.1608. IR (cm^{-1}) 2926, 1723, 1686.

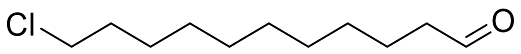

1q

Synthesised according to procedure B with 1-chloro-dodecan-1-ol (0.0414 g, 0.2 mmol.) to yield **1q** as a brown oil (0.0397 g, 97 %). 1H NMR (500 MHz, $CDCl_3$): δH 9.75 (t, *J* = 1.9 Hz, 1H), 3.52 (t, *J* = 6.8 Hz, 2H), 2.41 (td, *J* = 7.4, 1.9 Hz, 2H), 1.79 – 1.71 (m, 2H), 1.65 – 1.57 (m, 2H), 1.45 –1.37 (m, 2H), 1.34 – 1.23 (m, 10H). 13C{1H} NMR (126 MHz, $CDCl_3$): δ 203.08, 45.28, 44.01, 32.71, 29.82, 29.35, 29.21, 28.91, 26.94, 22.14. 1H and 13C NMR spectra are in acoordance with the literature.[11]

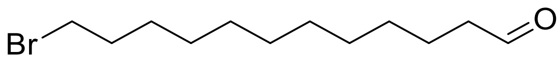

1r

Synthesized according to procedure B with 12-bromo-dodecan-1-ol (0.0530 g, 0.2 mmol.). Purification by flash column chromotography (100 % petroleum ether to 10 % EtOAc in petroleum ether) yielded a pale yellow oil (0.0384 g, 73 %). 1H NMR (500 MHz, $CDCl_3$): δH 9.74 (t, *J* = 1.9 Hz, 1H), 3.39 (t, *J* = 6.9 Hz, 2H), 2.40 (td, *J* = 7.3, 1.9 Hz, 2H), 1.83 (dt, *J* = 14.5, 6.9 Hz, 2H), 1.64 – 1.59 (m, 2H), 1.43 –1.40 (m, 2H), 1.32 – 1.18 (m, 14H). 13C{1H} NMR (126 MHz, $CDCl_3$): δ 202.98, 43.99, 34.11, 32.90, 29.50, 29.46, 29.43, 29.40, 29.22, 28.81, 28.23, 22.15. 1H and 13C NMR spectra are in accordance with the literature.[12]

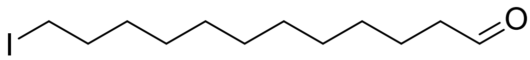

1s

Synthesized according to procedure B with 12-iodo-dodecan-1-ol (0.0624 g, 0.2 mmol.). NMR yield (75 %) was calculated using dibromomethane (0.0341 g, 0.196 mmol). Purification by flash column chromotography (100 % petroleum ether to 10 % EtOAc in petroluem ether) yielded a yellow oil (0.0186 g, 30 %). 1H NMR (500 MHz, $CDCl_3$): δH 9.58 (t, *J* = 1.9 Hz, 1H), 3.00 (t, *J* = 7.1 Hz, 2H), 2.24 (td, *J* = 7.4, 1.9 Hz, 2H), 1.63 (t, *J* = 7.1 Hz, 2H), 1.46 – 1.42 (m, 2H), 1.17 – 1.03 (m, 14H). 13C{1H} NMR (126 MHz, $CDCl_3$): δ 203.13, 44.04, 33.69, 33.66, 30.61, 29.56, 29.49, 29.45, 29.27, 28.64, 22.19, 7.54. ^{1}H and ^{13}C NMR are in accordance with the literature.[13]

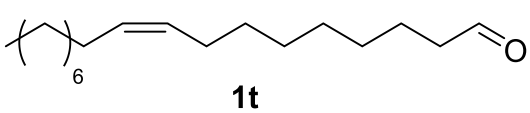

1t

Synthesized according to procedure B with (Z)-octadec-9-en-1-ol (0.0537 g, 0.2 mmol). NMR yield (93 %) was calculated using dibromomethane (0.0390 g, 0.22 mmol.). Purification by flash column chromotography (100 % petroleum ether to 10 % EtOAc in petroleum ether) gave a colourless oil (0.0285 g, 53 %). %). 1H NMR (400 MHz, $CDCl_3$): δH 9.75 (t, *J* = 1.9 Hz, 1H), 5.35 – 5.31 (m, 2H), 2.40 (td, *J* = 7.4, 1.9 Hz, 2H), 2.07 – 1.89 (m, 4H), 1.61 (t, *J* = 7.4 Hz, 2H), 1.35 – 1.23 (m, 22H), 0.89 – 0.84 (t, 3H). 13C{1H} NMR (100 MHz, $CDCl_3$): δ 202.97, 130.13, 129.78, 43.99, 31.99, 29.85, 29.75, 29.60, 29.44, 29.40, 29.33, 29.22, 29.13, 27.30, 27.23, 22.76, 22.16, 14.19. ^{1}H and ^{13}C NMR spectra are in accordance with the literature.[14]

1g Scale Reaction

To a 20 cm^3 eSym vial was added (Z)-octadec-9-en-1-ol (1.0042 g, 3.725 mmol). 3-Å molecular sieves (0.6 g), NBu_4BF_4 (0.2636 g, 0.8 mmol.), DCM (20 cm^3), 1,1,1,3,3,3-hexafluoro-2-propanol (0.78 mL, 7.45 mmol.), 2,4,6-trimethyl pyridine (0.0.98 mL, 7.45 mmol) and (methylsulfanyl)methane (0.276 mL, 3.725 mmol). The resulting mixture was electrolysed at a constant current of 10 mA while stirring at 600 rpm for 2.5 F/mol with a carbon anode and stainless steel cathode. The solution was transferred to a f funnel and the vial and electrodes were washed with DCM (20 mL). The product was diluted with diethyl ether (200 mL) and hexanes (200 mL) before being washed with 1 M HCl (3x400 mL) and deionised water (3x 400 mL). The organic layer was dried over anhydrous Na_2SO_4, filtered and solvent was removed under reduced pressure. Purification by flash column chromotography (100 % petroleum ether to 20 % EtOAc in petroleum ether) yielded**1t** as a colourless oil (0.6172 g, 64 %).

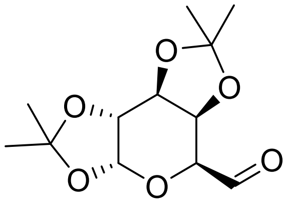

1u

Synthesized according to procedure B with (3aR,5S,5aR,8aS,8bR)-2,2,7,7-tetramethyltetrahydro-5H-bis([1,3]dioxolo)[4,5-b:4',5'-d]pyran-5-carbaldehyde (0.0520 g, 0.2 mmol.). NMR yield (53 %) was calculated using dibromomethane (0.0290 g, 0.167 mmol.) as an internal standard. The product could not be isolated from the reaction mixture due to its instability on flash column media. 1H NMR (400 MHz, $CDCl_3$): δH 9.60 (s, 1H), 5.65 (d, *J* = 4.9 Hz, 1H), 4.63 (dd, *J* = 7.8, 2.5 Hz, 1H), 4.60 – 4.57 (m, 1H), 4.37 (dd, *J* = 4.9, 2.5 Hz, 1H), 4.18 (d, *J* = 2.2 Hz, 1H), 1.49 (s, 3H), 1.42 (s, 3H), 1.33 (s, 3H), 1.30 (s, 3H). 13C{1H} NMR (100 MHz, $CDCl_3$): δ 200.35, 110.12, 109.13, 96.32, 73.29, 71.79, 70.56, 70.46, 26.07, 25.86, 24.87, 24.31.[15]

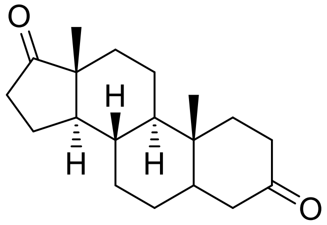

1v

Synthesized according to procedure B with (8S,9R,10R,13R,14R)-3-hydroxy-10,13-dimethylhexadecahydro-17H-cyclopenta[a]phenanthren-17-one (0.0580 g, 0.2 mmol.) to give a **1v** as a brown solid (0.0340 g, 59 %). 1H NMR (400 MHz, $CDCl_3$): δH 2.50 – 2.21 (m, 4H), 2.13 – 1.92 (m, 4H), 1.84 – 1.80 (m, 2H), 1.70 – 1.25 (m, 12H), 1.06 – 0.96 (m, 4H), 0.88 – 0.87 (m, 3H), 0.86 – 0.81 (m, 1H). 13C{1H} NMR (100 MHz, $CDCl_3$): δ 221.78, 212.68, 66.79, 53.92, 51.29, 47.91, 46.68, 44.56, 38.50, 38.07, 35.91, 35.87, 35.01, 31.50, 30.59, 28.65, 21.85, 20.77, 13.86, 11.52. ^{1}H and ^{13}C NMR are in accordance with the literature.[16]

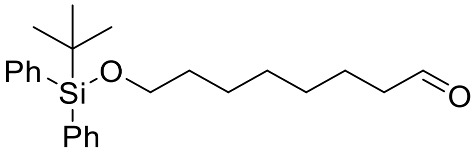

1w

Synthesized according to procedure B with 8-((tert-butyldiphenylsilyl)oxy)octan-1-ol (0.0769 g, 0.2 mmol.) to yield **1w** as a pale yellow oil (0.0681 g, 0.2 mmol.). 1H NMR (500 MHz, $CDCl_3$): δH 9.77 (t, *J* = 1.9 Hz, 1H), 7.69 (dd, *J* = 7.9, 1.6 Hz, 4H), 7.44 – 7.37 (m, 6H), 3.67 (t, *J* = 6.5 Hz, 2H), 2.42 (td, *J* = 7.4, 1.9 Hz, 2H), 1.65 – 1.54 (m, 4H), 1.41 – 1.24 (m, 8H), 1.07 (s, 9H). 13C{1H} NMR (126 MHz, $CDCl_3$): δ 203.04, 135.68, 134.21, 129.62, 127.69, 63.98, 44.00, 32.57, 29.23, 29.19, 26.99, 25.69, 22.13, 19.34. 1H and 13C NMR spectra are in accordance with the literature.[17]

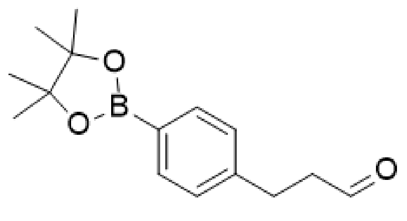

1x

Synthesized according to procedure B with 3-(4-(4,4,5,5-tetramethyl-1,3,2-dioxaborolan-2-yl)phenyl)propan-1-ol (0.0524 g, 0.2 mmol.) to give **1x** as a pale yellow oil (0.0483 g, 93 %). 1H NMR (500 MHz, $CDCl_3$): δH 9.81 (t, *J* = 1.4 Hz, 1H), 7.75 (d, *J* = 8.0 Hz, 2H), 7.21 (d, *J* = 8.0 Hz, 2H), 2.97 (t, *J* = 7.6 Hz, 2H), 2.78 (td, *J* = 7.6, 1.4 Hz, 2H), 1.34 (s, 12H). 13C{1H} NMR (126 MHz, $CDCl_3$): δ 201.59, 143.80, 135.25, 127.86, 83.86, 45.23, 28.40, 24.97. Carbon signal adjacent to boron was not observed due to B-C coupling. 1H and 13C NMR spectra in accordance with the literature. CAS number: 2755167-47-4

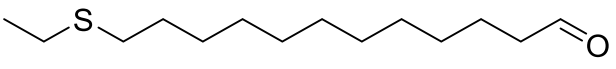

1y

Synthesized according to procedure B with 12-(ethylthio)dodecan-1-ol (0.0492 g, 0.2 mmol). NMR yield (56 %) calculated using dibromomethane (0.0311 g, 0.18 mmol.) as an internal standard. Purification by flash column chromotography (100 % petroleum ether to 20 % EtOAc in petroleum ether) yielded **1y** as a colourless oil. 0.0131 g, 27 %). 1H NMR (500 MHz, $CDCl_3$): δH 9.76 (t, *J* = 1.9 Hz, 1H), 2.55 – 2.48 (m, 4H), 2.41 (td, *J* = 7.4, 1.9 Hz, 2H), 1.65 – 1.49 (m, 6H), 1.26 (m, 15H). 13C{1H} NMR (126 MHz, $CDCl_3$): δ 203.10, 44.01, 31.75, 29.73, 29.58, 29.48, 29.43, 29.33, 29.24, 29.22, 29.04, 26.00, 22.16, 14.91. IR (cm^{-1}) 2729, 1734. Melting point: 48-51 °C No mass peak corresponding to the product found via HRMS.

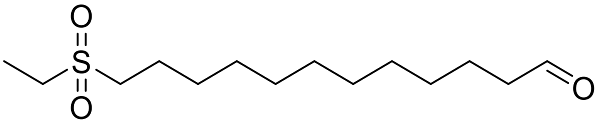

1z

Synthesized according to procedure B with 12-(ethylsulfonyl)dodecan-1-ol (0.0557 g, 0.2 mmol.) to yield **1z** as a white solid (0.0530 g, 91 %). %). 1H NMR (500 MHz, $CDCl_3$): δH 9.74 (t, *J* = 1.9 Hz, 1H), 2.97 (d, *J* = 7.6 Hz, 4H), 2.41 (td, *J* = 7.4, 1.9 Hz, 2H), 1.85 – 1.75 (m, 2H), 1.62 – 1.59 (m, 2H), 1.30 – 1.25 (m, 5H), 1.32 – 1.24 (m, 12H). 13C{1H} NMR (126 MHz, $CDCl_3$): δ 203.28, 51.95, 47.03, 43.96, 29.44, 29.38, 29.36, 29.26, 29.18, 29.10, 28.55, 22.10, 21.92, 6.69. IR (cm^{-1}) 2736, 1708. Melting point: 58 – 60 °C No mass peak corresponding to the product found via HRMS.

3.3: Synthesis of Materials for Mechanistic Studies

Synthesis of cyclododecan-1-d-1-ol

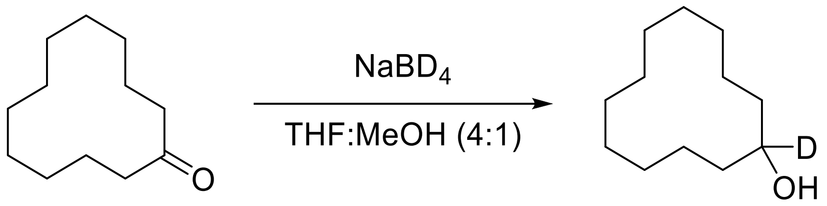

Scheme S10. Reaction conditions for the synthesis of cyclododecan-1-d-1-ol

Cyclododecanone (0.9142 g, 5 mmol.) was dissolved in methanol (2.5 mL) and THF (10 mL) before $NaBD_4$ (0.2848 g, 15 mmol.) was added in portions at 0°C. The reaction mixture was brought to room temperature and stirred for two hours before full conversion was noted by GC-MS analysis. The reaction was quenched with saturated aqueous $NaHCO_3$ (20 mL), followed by extraction with ethyl acetate (3x20 mL) The combined organic phases were washed with saturated aquoeus $NaHCO_3$ (3 x 40 mL) before solvent was removed under reduced pressure to yield cyclododecan-1-d-1-ol as a white powder (0.8792 g, 95 %) 1H NMR (500 MHz, $CDCl_3$): δH 1.48 – 1.45 (m, 2H), 1.36 – 0.98 (m, 21H). 1H NMR in accordance with the literature.[18]

Synthesis of methyl((4-phenylbutoxy)methyl)sulfane

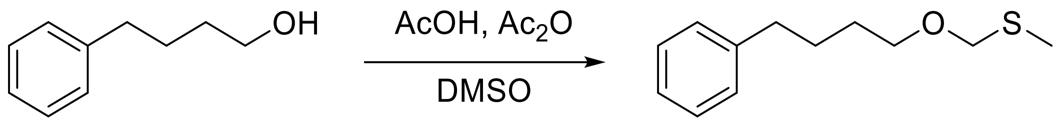

Scheme S11. Reaction conditions for the synthesis of methyl((4-phenylbutoxy)methyl)sulfane

Phenylbutan-1-ol (1.5 g, 10 mmol) was stirred in AcOH (5 mL), Ac_2O (1.5 mL) and DMSO (20 mL) for 72 hours before being quenched with Na_{2}-CO_3 (25 mL) The product was extracted with ethyl acetate (3 x 50 mL) before solvent was removed under reduced pressure followed by purification via flash column chromotography (100 % petroleum ether to 40 % EtOAc in petroleum ether) to yield the product as a pale yellow oil (0.9433 g, 45 %). 1H NMR (500 MHz, $CDCl_3$): δH 7.27 – 7.25 (m, 2H), 7.21 – 7.13 (m, 3H), 4.62 (s, 2H), 3.54 (t, *J* = 6.3 Hz, 2H), 2.64 (t, *J* = 7.5 Hz, 2H), 2.14 (s, 3H), 1.74 – 1.60 (m, 4H). 13C{1H} NMR (101 MHz, $CDCl_3$): δ 142.46, 128.51, 128.38, 125.81, 75.31, 68.03, 35.75, 29.11, 28.18, 14.04.

3.4: Incompatible substrates

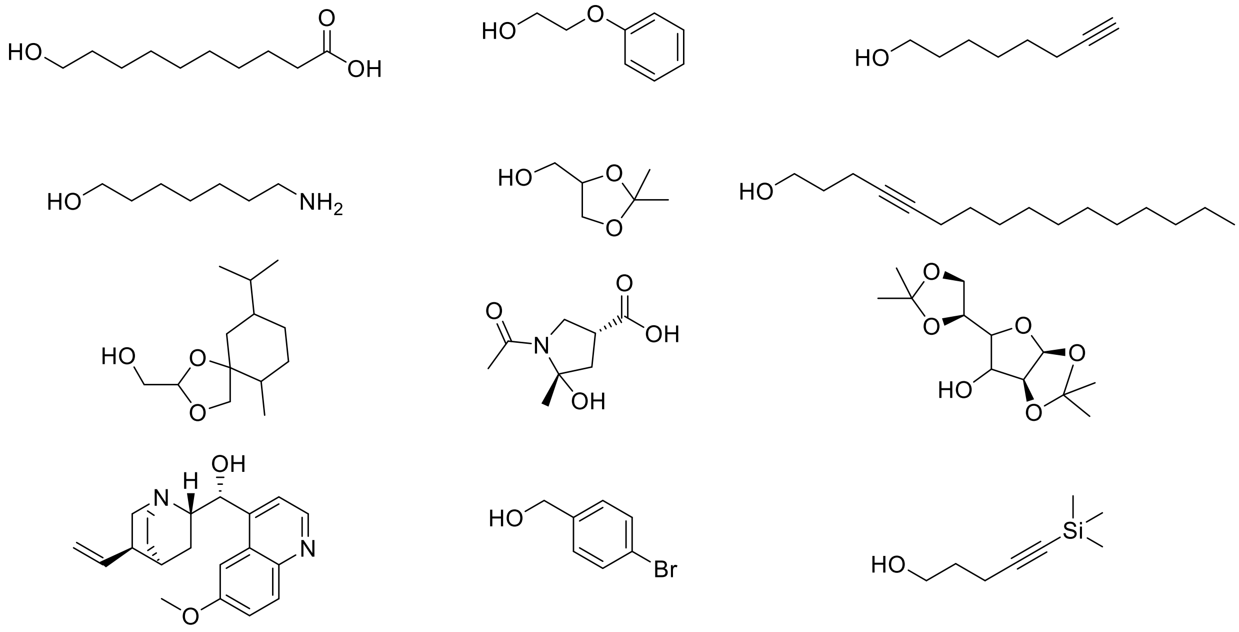

Figure S1. Substrates that resulted in no aldehyde or ketone formation in eSwern conditions

4. Mechanistic Studies

4.1: Cyclic Voltammetry

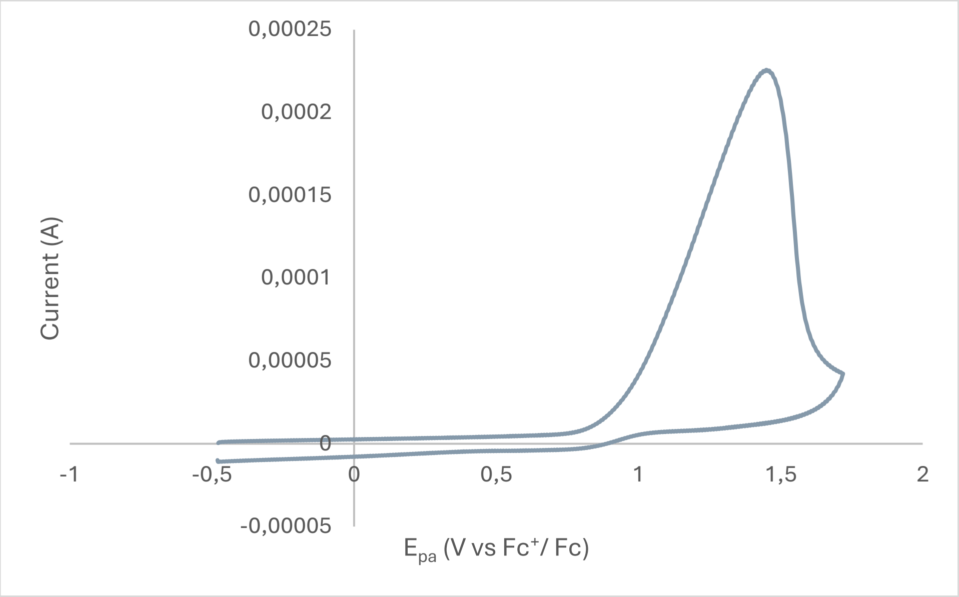

Figure S2. Cyclic voltammogram on glassy carbon (diameter 3mm); Pt wire as the counter electrode; Ag/AgCl as pseudo reference, of a 1mM solution of methyl sulfanymethane, 2-adamantylethan-1-ol, 2,4,6-Collidine and HFIP in DCM containing 0.1M of [NBu4][PF6], at 0.25 V.s-1at room temperature. IUPAC plotting convention. (Methylsulfanylmethane) exhibits a chemically non reversible oxidation at E_{pa} = 1 V vs Fc$^+$ / Fc

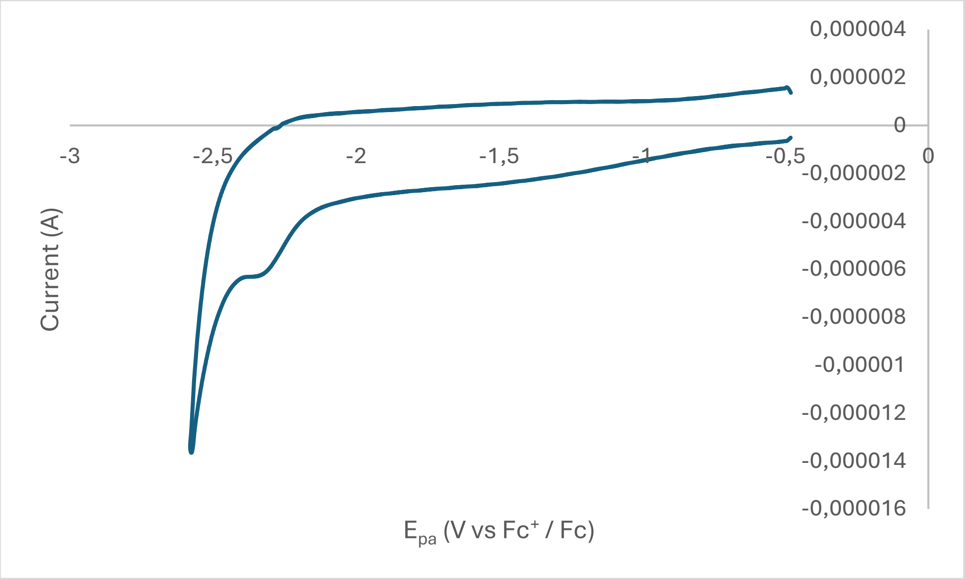

Figure S 3 Cyclic voltammogram on glassy carbon (diameter 3mm); Pt wire as the counter electrode; Ag/AgCl as pseudo reference, of a 1mM solution of (methylsulfanyl)methane, 2-adamantylethan-1-ol, 2,4,6-Collidine and HFIP in DCM containing 0.1M of [NBu4][PF6], at 0.25 V.s-1at room temperature. IUPAC plotting convention. HFIP exhibits a chemically non reversible oxidation at E_{pa} = -2.3 V vs Fc$^+$ / Fc

4.2: Deuterium labelled alcohol oxidation

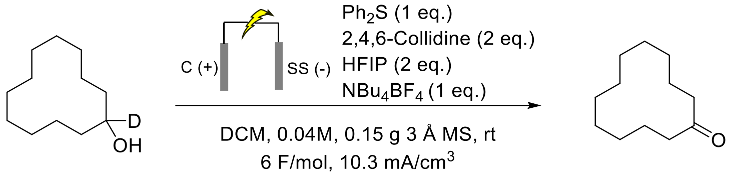

Scheme S11. Electrochemical oxidation of cyclododecan-1-d-1-ol

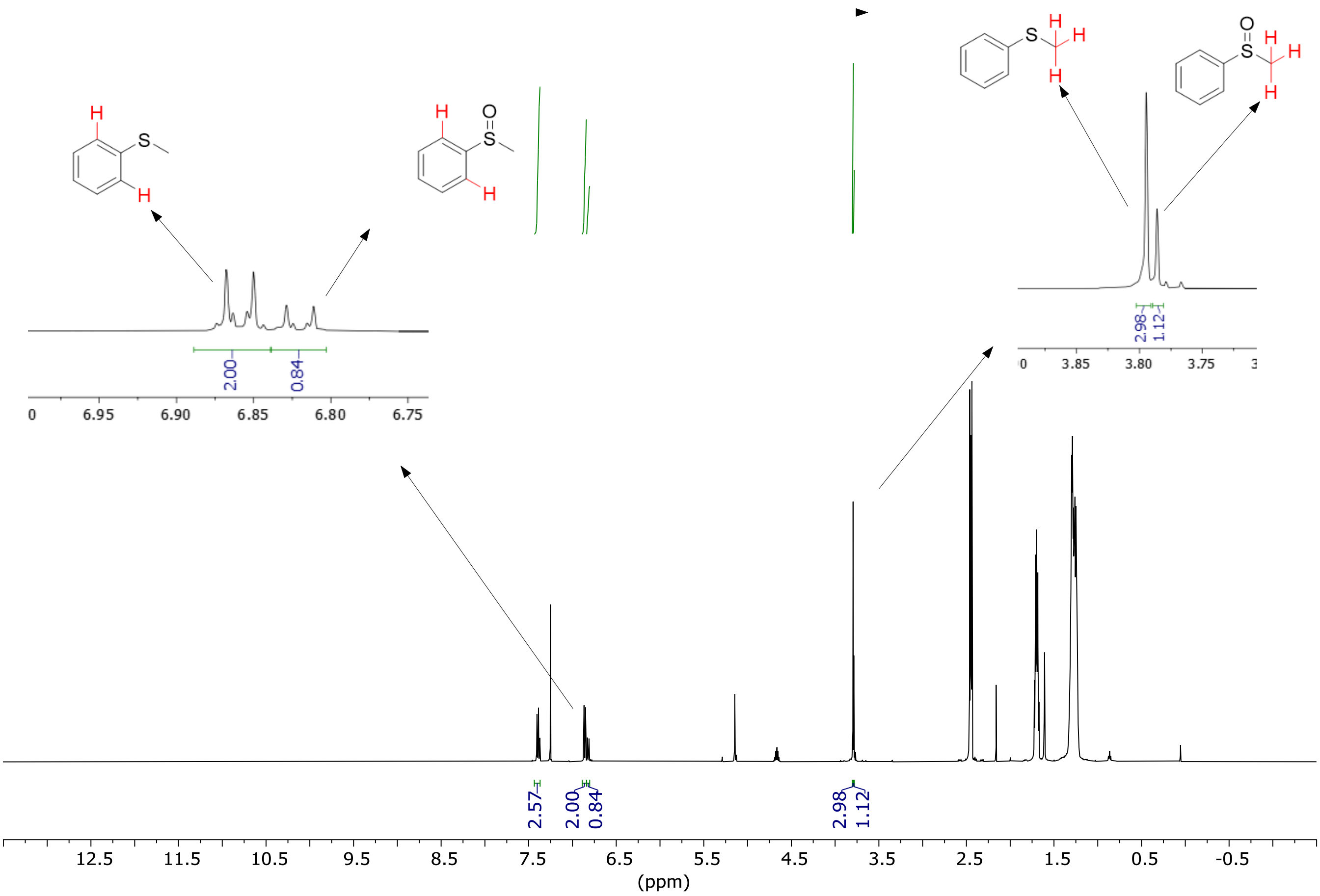

Figure S3. NMR spectrum of crude reaction mixture of oxidation on cyclododecan-1-d-1-ol. Integrations of sulfide mediator and its oxidized sulfoxide analogue show no evidence of deuterium incorporation.

5. References

1 T. L. Schull, L. Renée Olano and D. Andrew Knight, *Tetrahedron*, 2000, **56**, 7093–7097.
2 B. Xu, J. Lumb and B. A. Arndtsen, *Angew Chem Int Ed*, 2015, **54**, 4208–4211.
3 Q. Fan, D. Liu, Z. Xie, Z. Le, H. Zhu and X. Song, *J. Org. Chem.*, 2023, **88**, 14559–14570.
4 S. P. Bhoite and G. Suryavanshi, *Natural Product Research*, 2023, **37**, 3388–3394.
5 J. P. S. Choo, R. A. Kammerer, X. Li and Z. Li, *Adv Synth Catal*, 2021, **363**, 1714–1721.
6 A. 'Gus' Bakhoda, *Chem. Commun.*, 2024, **60**, 6937–6940.
7 M. Tapera, F. Borghi, J. L. Mayer-Figge, F. Mittendorf, I.-E. Celik, A. Gómez-Suárez and S. F. Kirsch, *Molecules*, 2024, **29**, 3710.
8 T. Pesnot, M. C. Gershater, J. M. Ward and H. C. Hailes, *Adv Synth Catal*, 2012, **354**, 2997–3008.
9 J. X. Qiao, D. Williams, P. Gill, L. Li, D. Norris, J. S. Tokarski, J. Wong, H. Qi, Y. Hafeji, D. P. Downes, B. Degnen, Y.-K. Wang, G. Locke, H. Fang, F. Yu, S. Xu, J. Naglich, J. Zhang, P. Nanjappa, C. Dai, L. Chourb, J. Napoline, R. Tester, C. Jorge, Y.-X. Li, A. Mathur, C. Barbieri, M. G. Soars, A. Venkatanarayan, E. Lees, R. M. Borzilleri, A. V. Gavai, M. Wichroski and T. G. M. Dhar, *J. Med. Chem.*, 2024, **67**, 19736–19754.
10 K. Kuramochi, S. Yukizawa, S. Ikeda, T. Sunoki, S. Arai, R. Matsui, A. Morita, Y. Mizushina, K. Sakaguchi, F. Sugawara, M. Ikekita and S. Kobayashi, *Bioorganic & Medicinal Chemistry*, 2008, **16**, 5039–5049.
11 M. Uemura, K. Yagi, M. Iwasaki, K. Nomura, H. Yorimitsu and K. Oshima, *Tetrahedron*, 2006, **62**, 3523–3535.
12 S. G. Taher, J. R. Al Dulayymi, H. G. Tima, H. M. Ali, M. Romano and M. S. Baird, *Tetrahedron*, 2016, **72**, 3863–3876.
13 J. Barluenga, F. González-Bobes, M. C. Murguía, S. R. Ananthoju and J. M. González, *Chemistry A European J*, 2004, **10**, 4206–4213.
14 S. Kouba, J. Braire, R. Félix, A. Chantôme, P.-A. Jaffrès, J. Lebreton, D. Dubreuil, M. Pipelier, X. Zhang, M. Trebak, C. Vandier, M. Mathé-Allainmat and M. Potier-Cartereau, *European Journal of Medicinal Chemistry*, 2020, **186**, 111854.
15 S. Kamo, K. Hori and K. Sugita, *Org. Biomol. Chem.*, 2024, **22**, 5960–5964.
16 M. S. Yusubov, P. S. Postnikov, R. Ya. Yusubova, A. Yoshimura, G. Jürjens, A. Kirschning and V. V. Zhdankin, *Adv Synth Catal*, 2017, **359**, 3207–3216.
17 M. Borie-Guichot, M. Lan Tran, V. Garcia, A. Oukhrib, F. Rodriguez, C.-O. Turrin, T. Levade, Y. Génisson, S. Ballereau and C. Dehoux, *Bioorganic Chemistry*, 2024, **146**, 107295.
18 X. Meng, C. Che, Y. Dong, Q. Liu and W. Wang, *Org. Lett.*, 2024, **26**, 8961–8966.

6. NMR Spectra

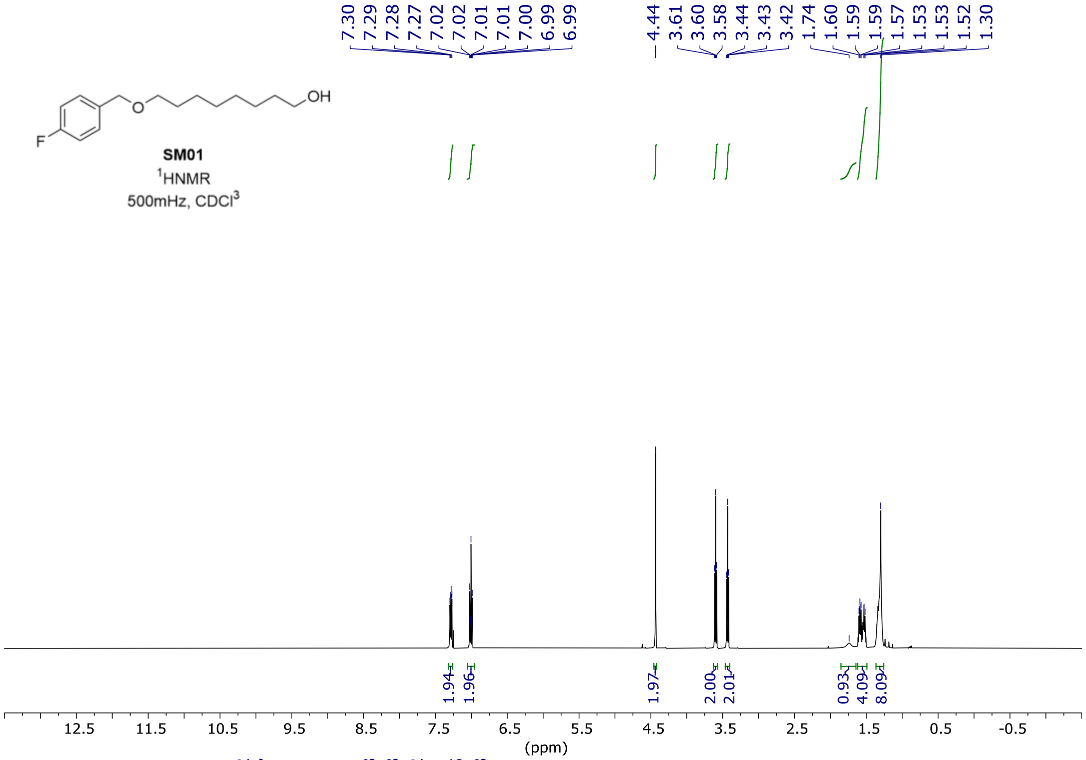

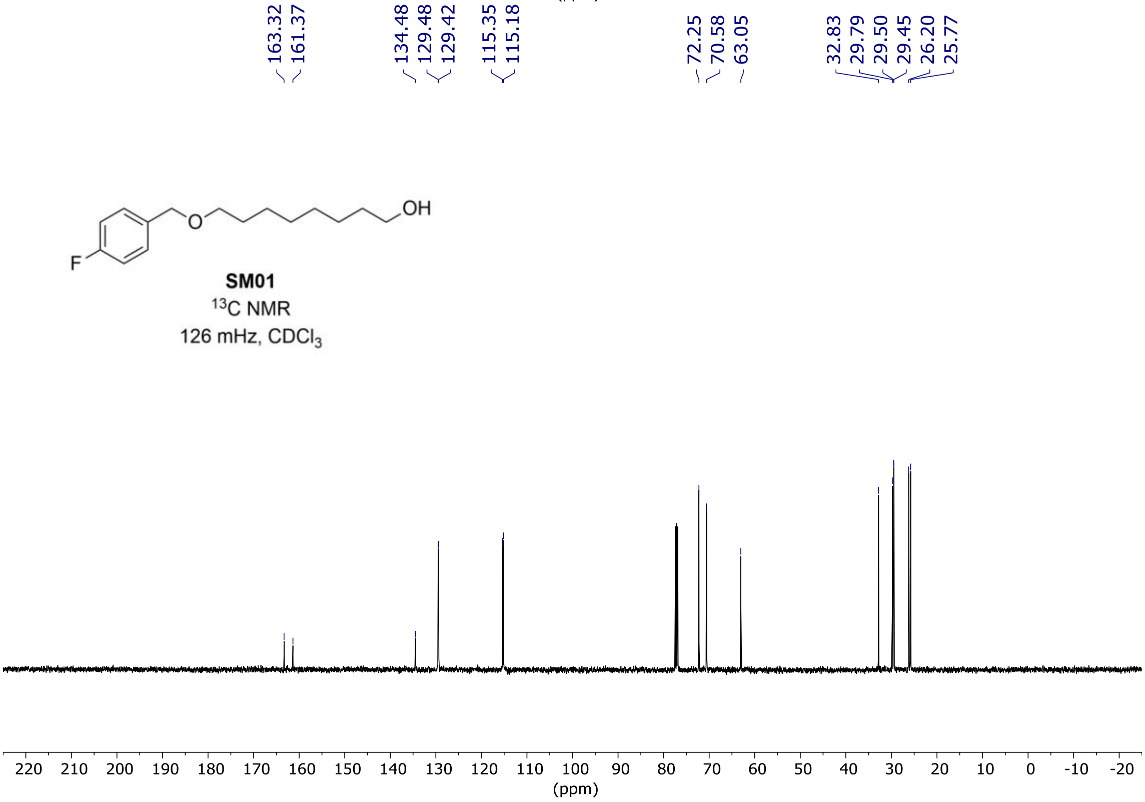

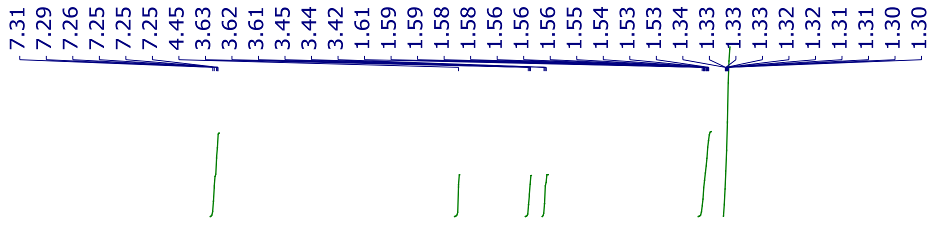
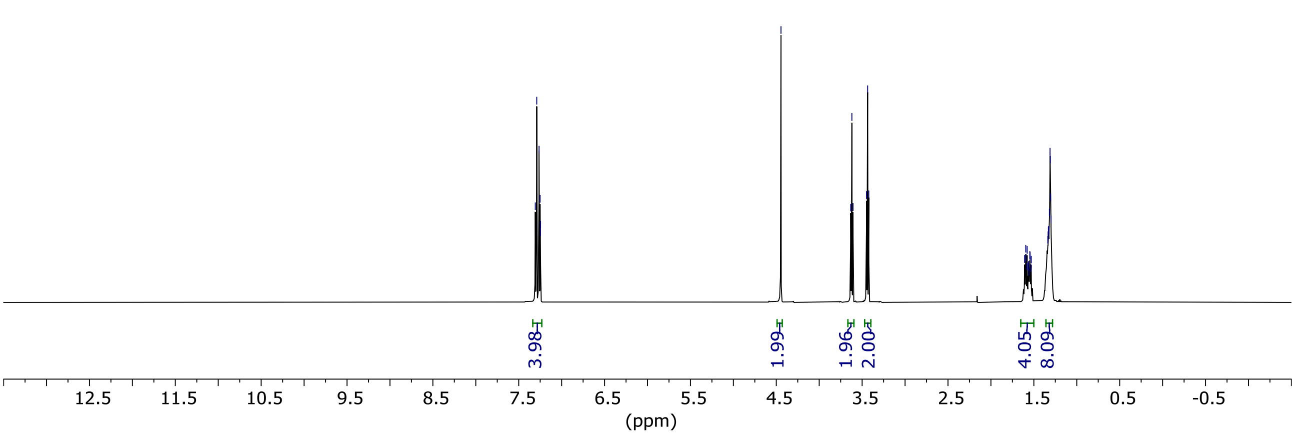

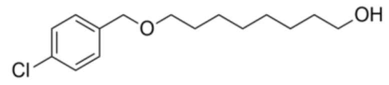

SM02
^{1}H NMR
500 mHz, $CDCl_3$

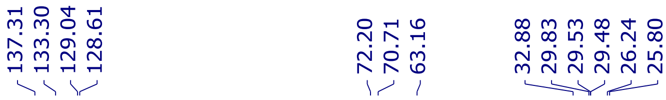
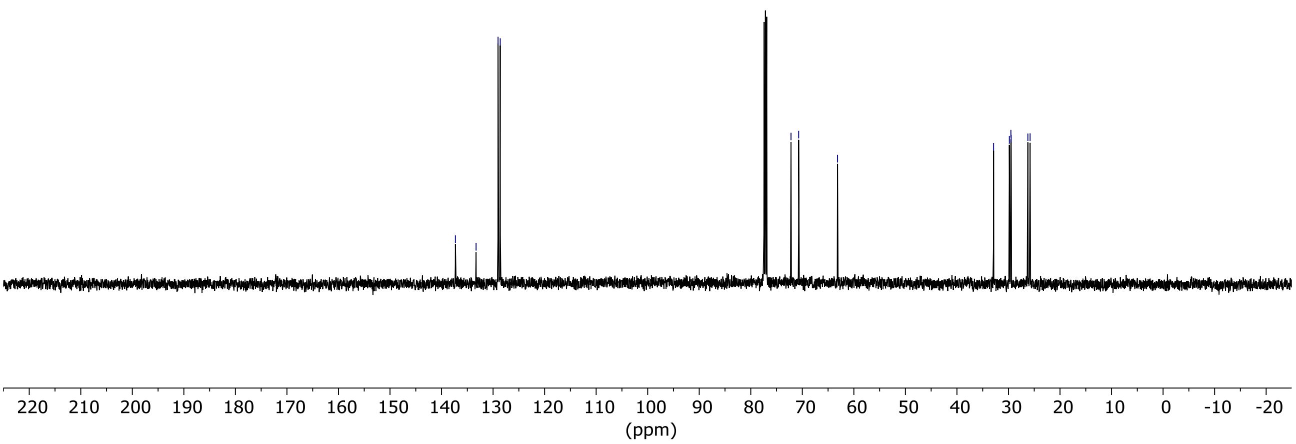

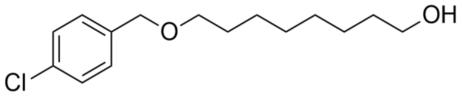

SM02
^{13}C NMR
126 mHz, $CDCl_3$

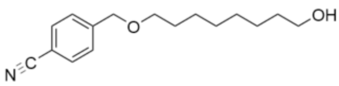

SM03
^{1}H NMR
400 mHz, $CDCl_3$

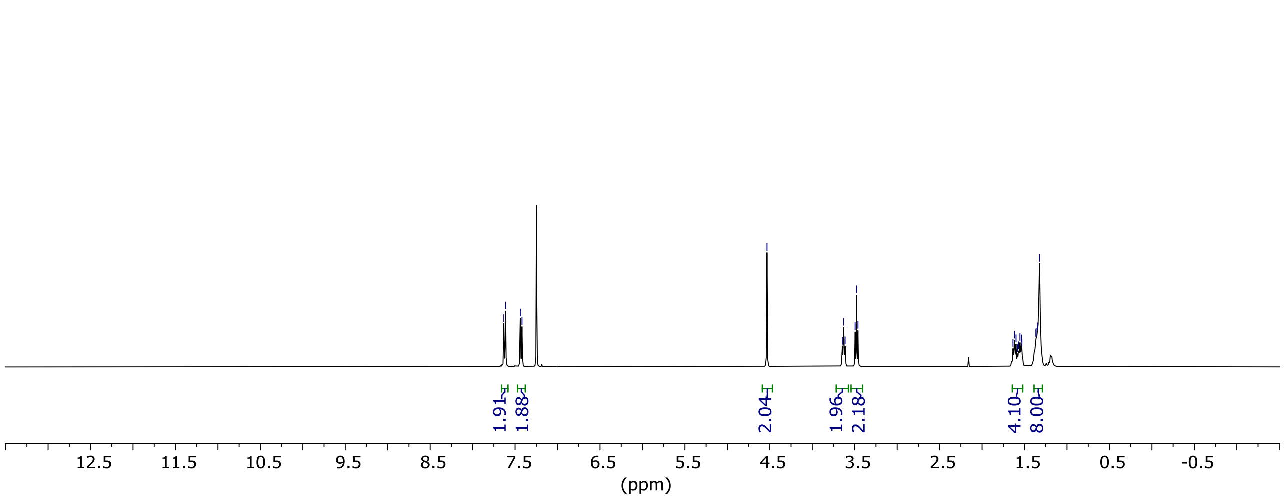

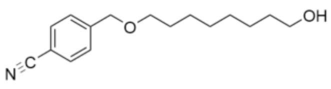

SM03
^{13}C NMR
100 mHz, $CDCl_3$

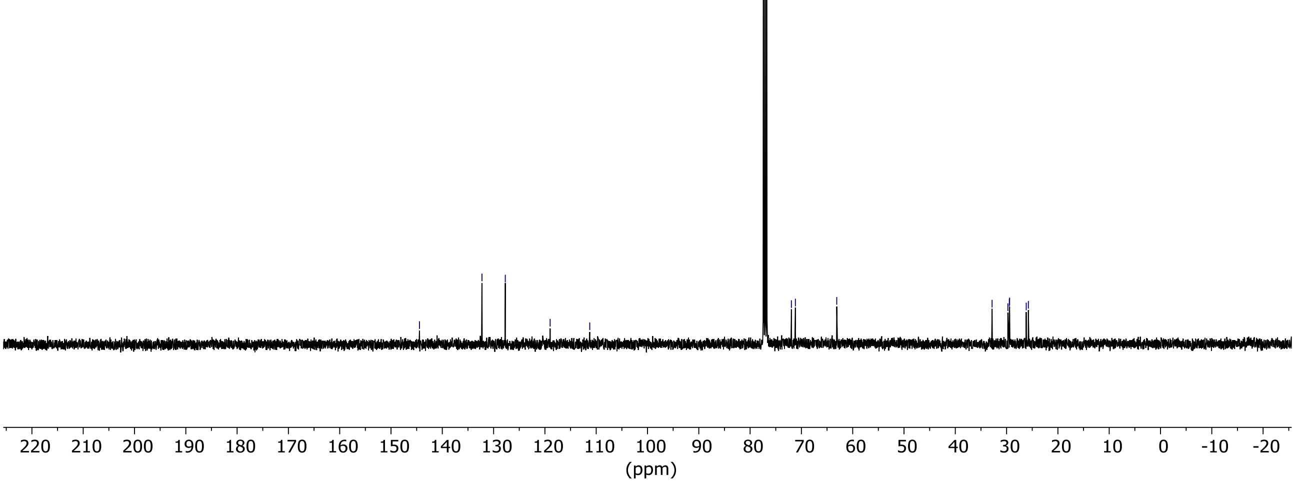

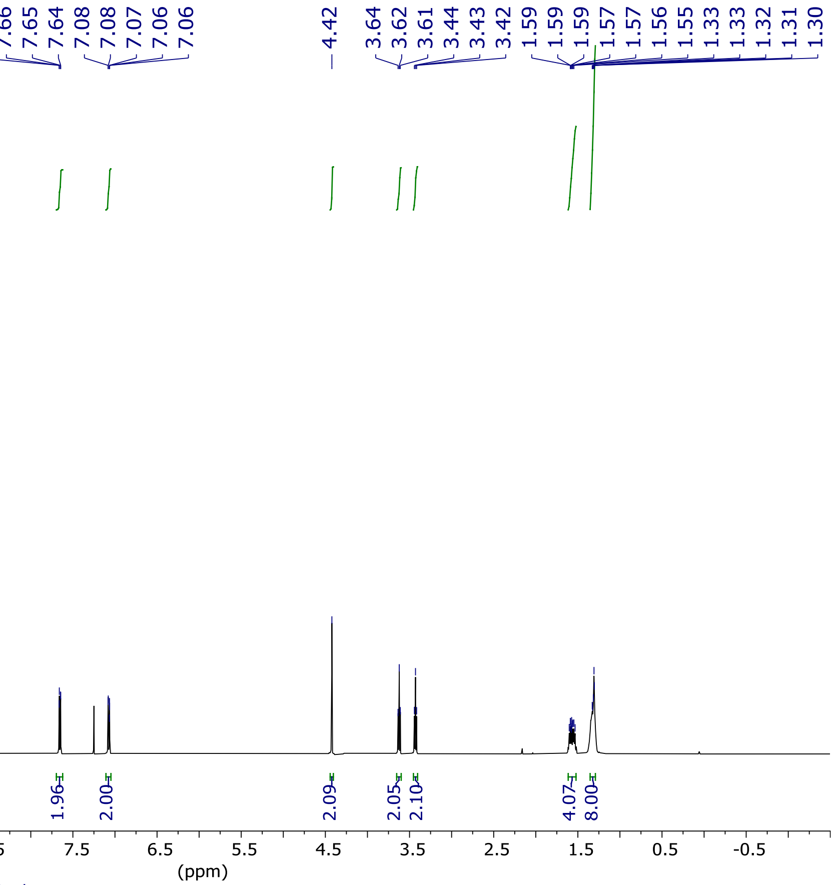

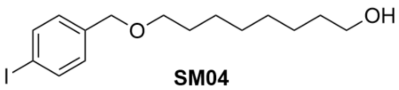

SM04

^{1}H NMR

500 mHz, $CDCl_3$

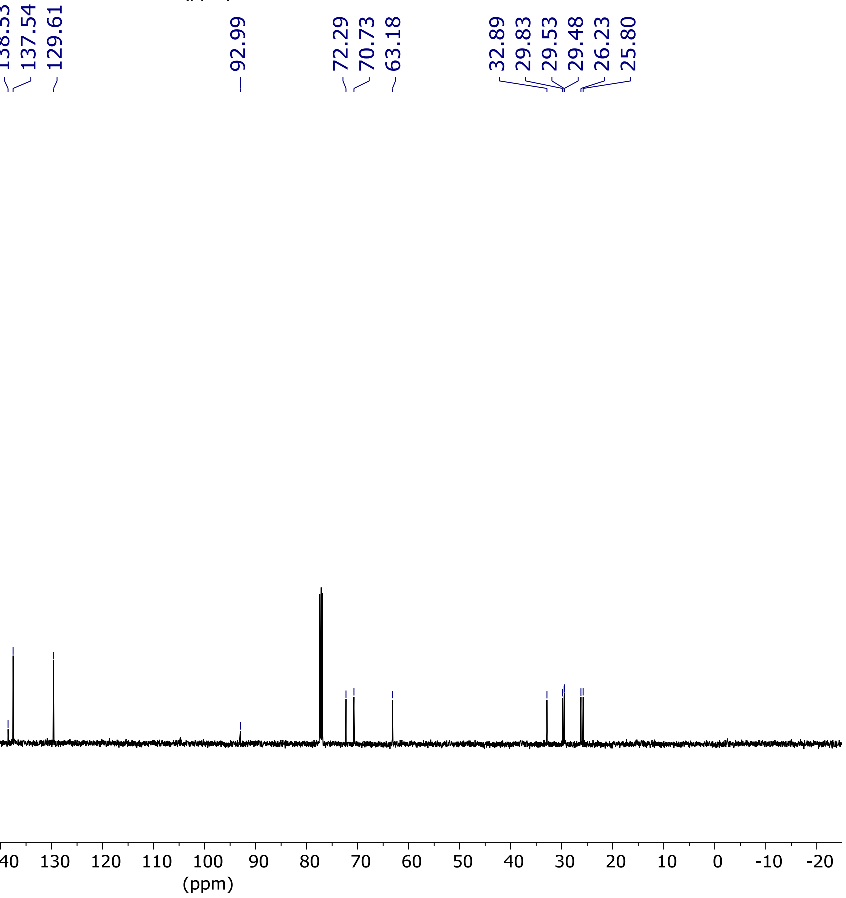

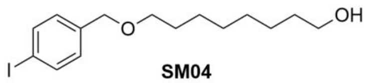

SM04

^{13}C NMR

126 mHz, $CDCl_3$

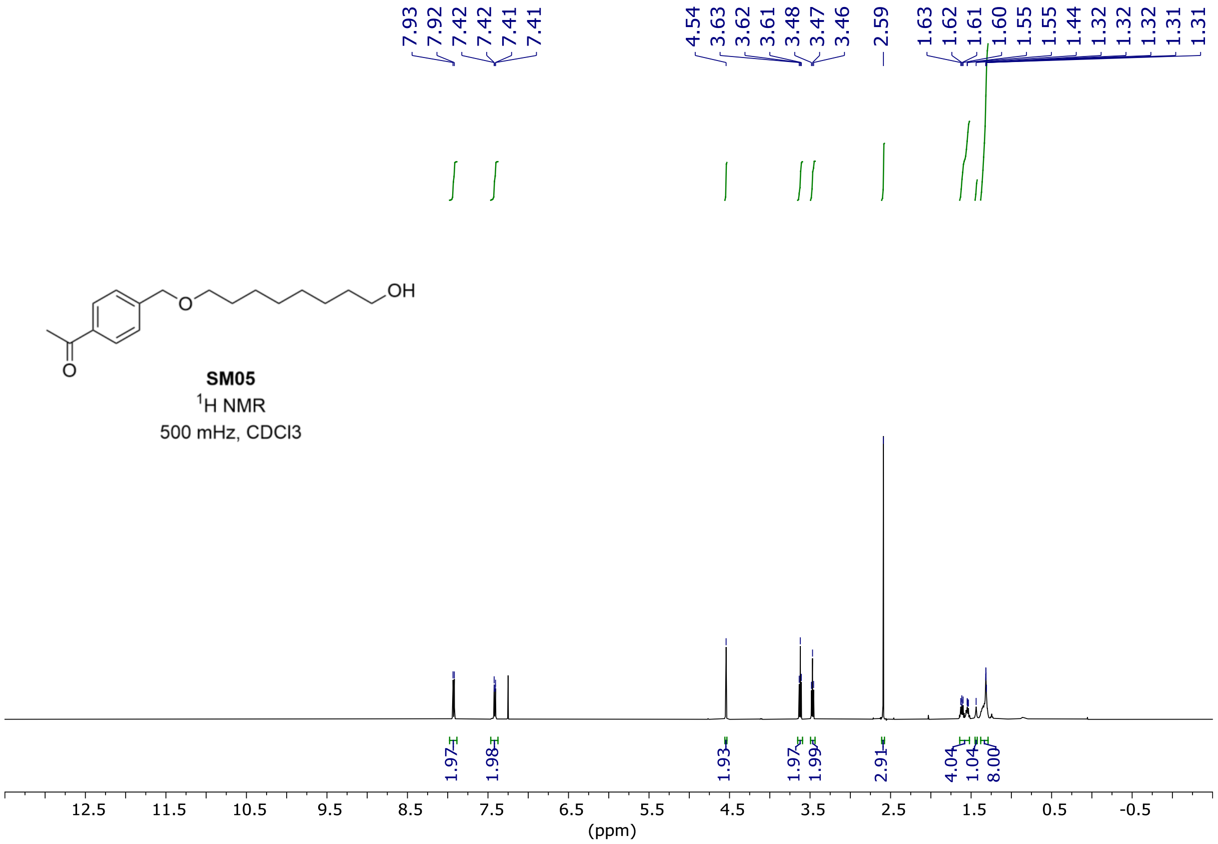

SM05

^{1}H NMR

500 mHz, CDCl3

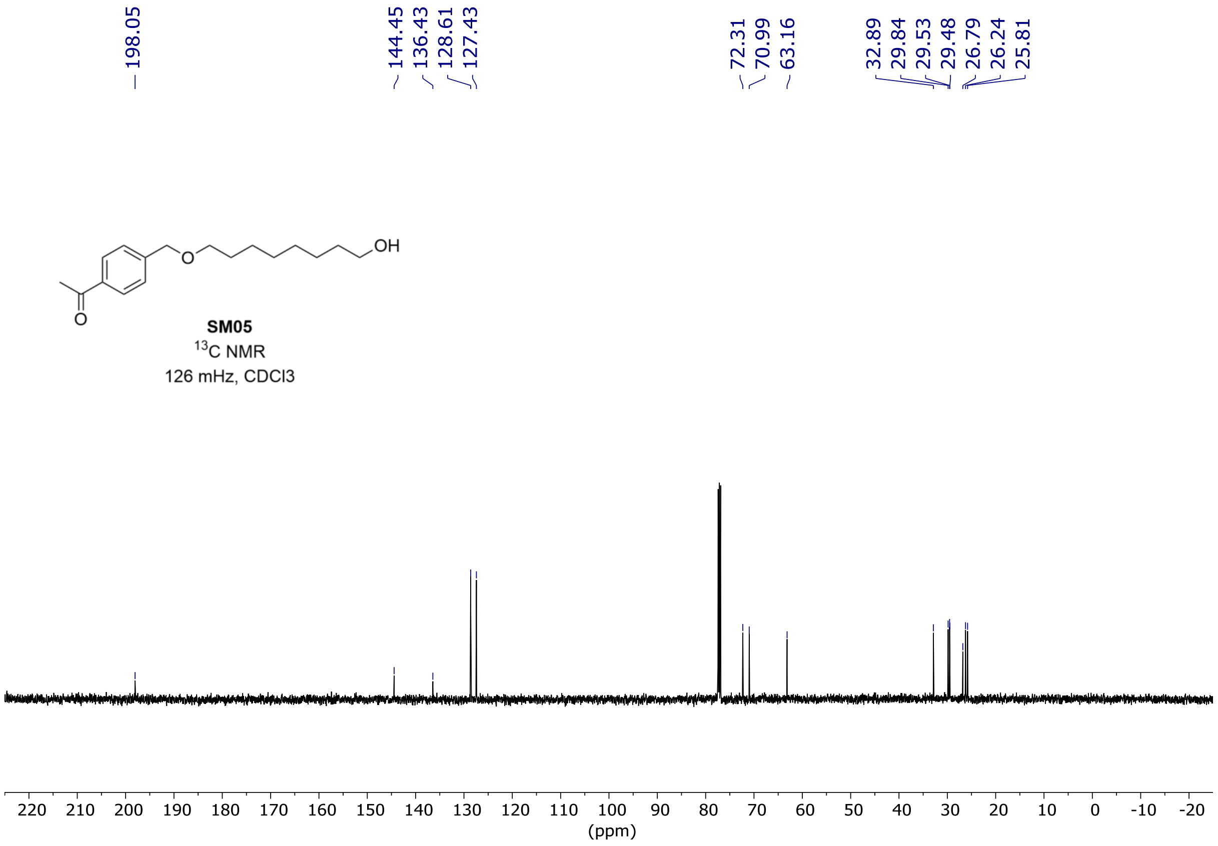

SM05

^{13}C NMR

126 mHz, CDCl3

SM06

^{1}H NMR

400 mHz, $CDCl_3$

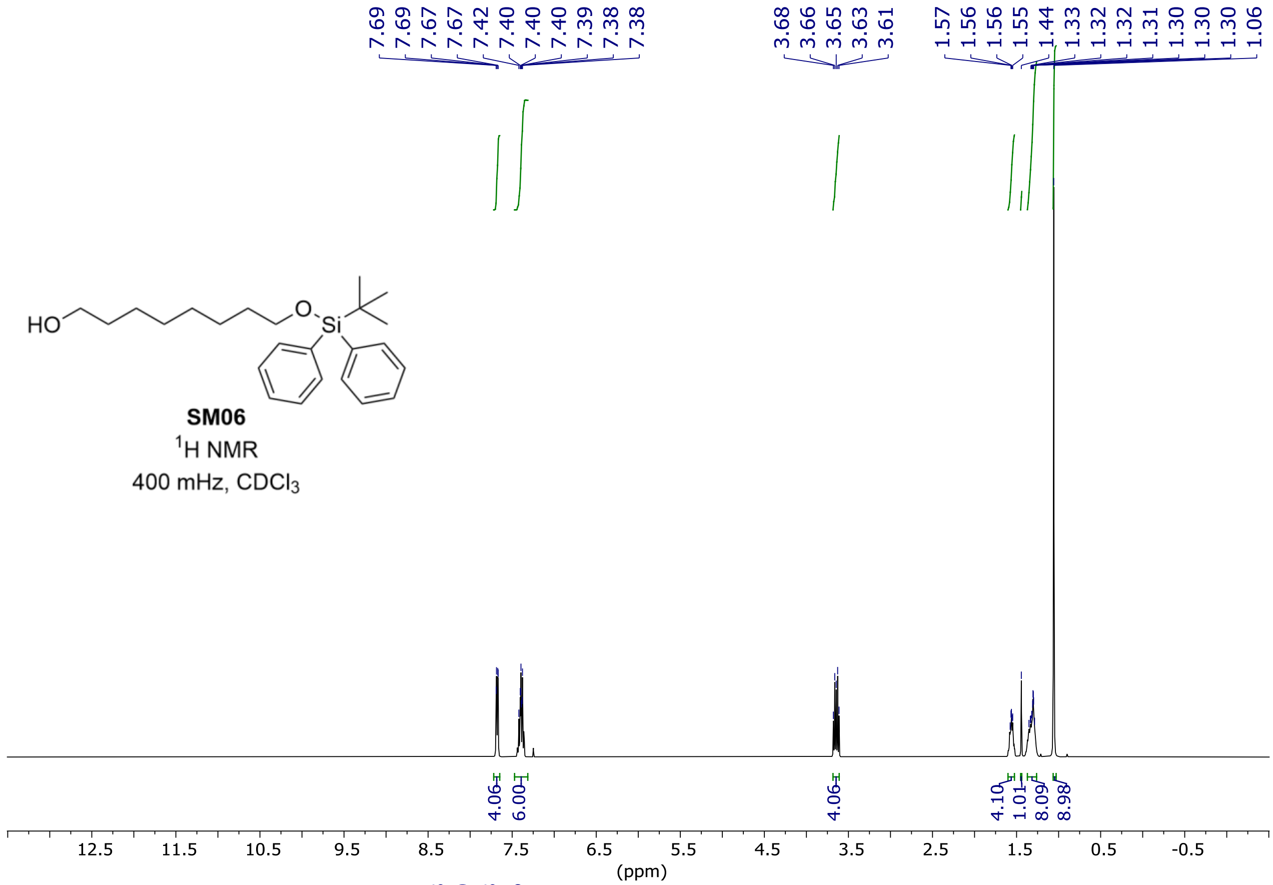

SM06

^{13}C NMR

100 mHz, $CDCl_3$

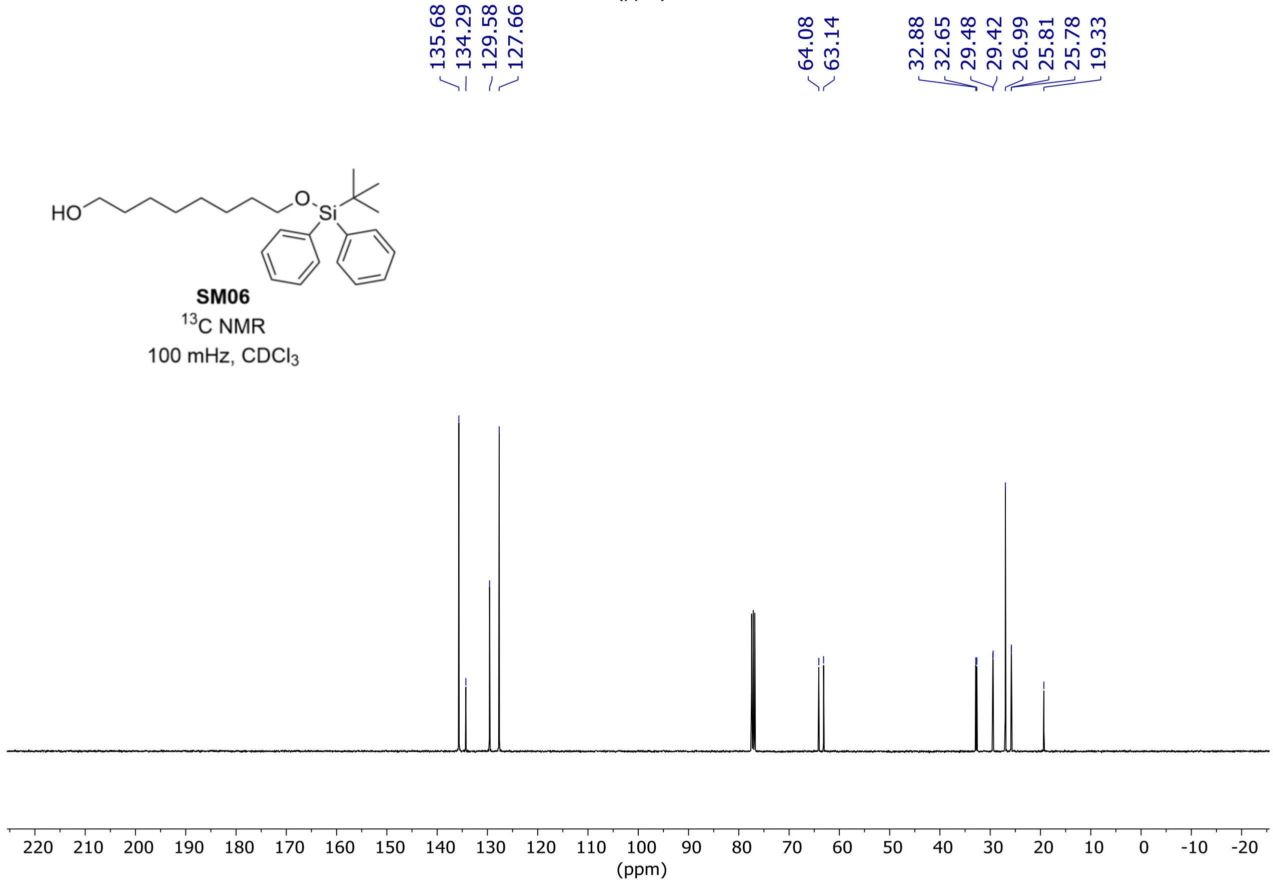

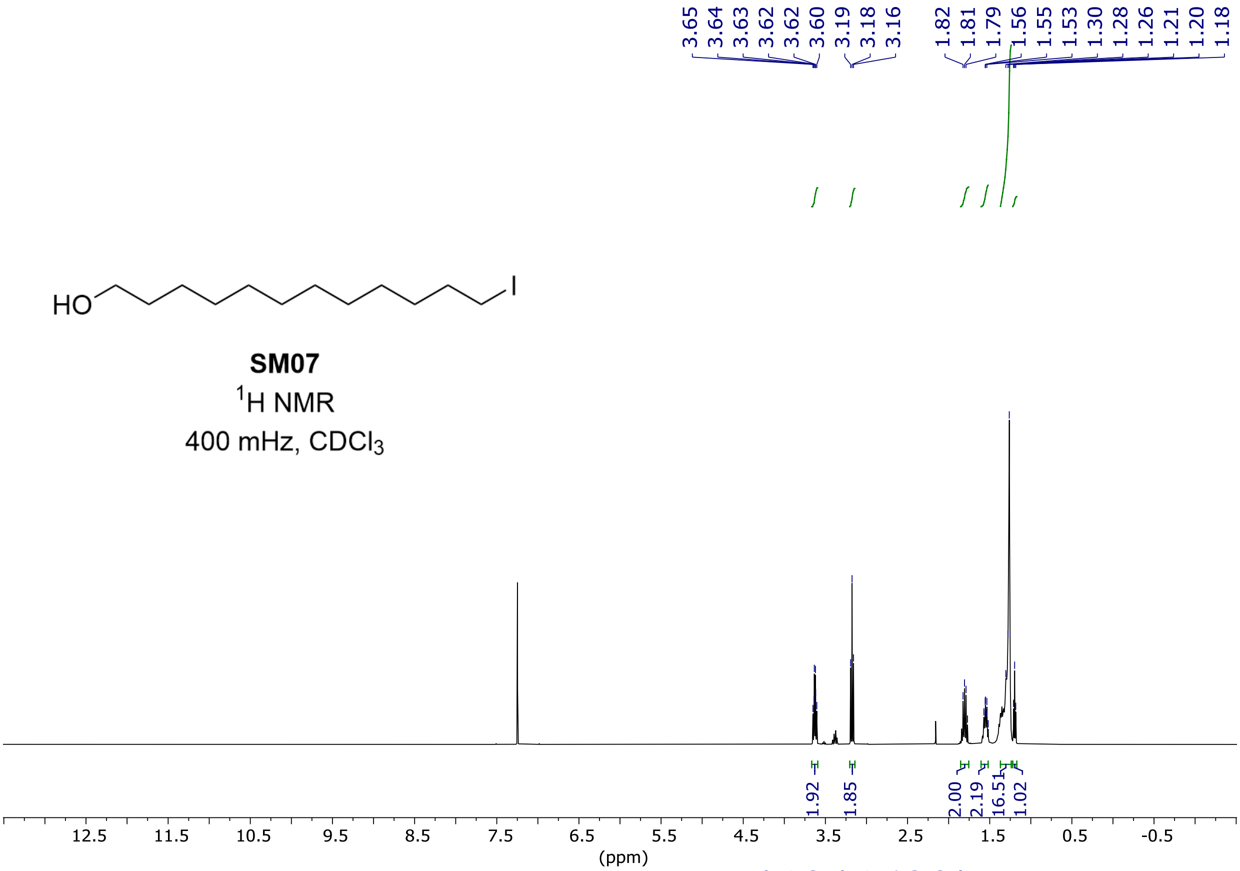

3.65 3.64 3.63 3.62 3.62 3.60 3.19 3.18 3.16 1.82 1.81 1.79 1.56 1.55 1.53 1.30 1.28 1.26 1.21 1.20 1.18
HO
I
SM07
^{1}H NMR
400 mHz, $CDCl_3$
1.92 1.85 2.00 2.19 16.51 1.02
12.5 11.5 10.5 9.5 8.5 7.5 6.5 5.5 4.5 3.5 2.5 1.5 0.5 -0.5
(ppm)

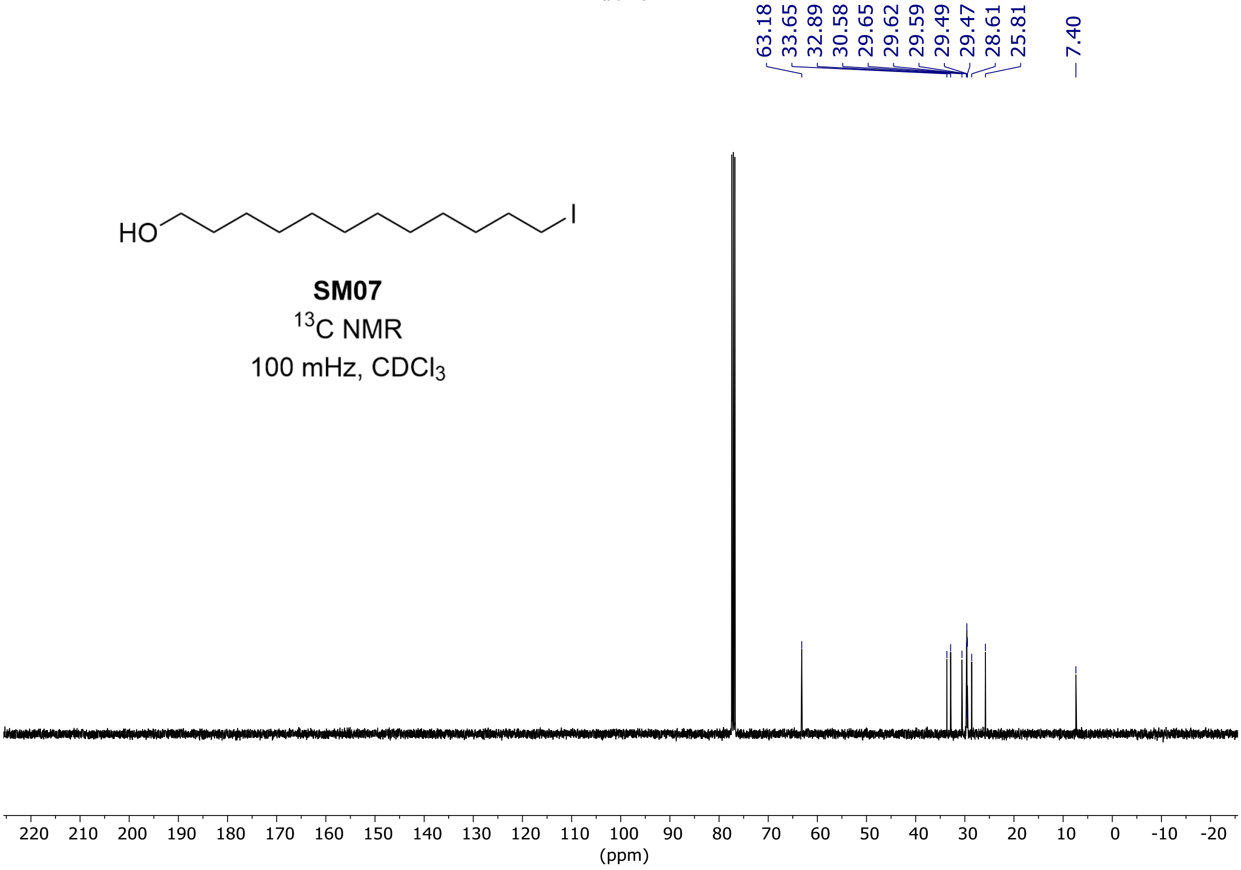

63.18 33.65 32.89 30.58 29.65 29.62 29.59 29.49 29.47 28.61 25.81 7.40
HO
I
SM07
^{13}C NMR
100 mHz, $CDCl_3$
220 210 200 190 180 170 160 150 140 130 120 110 100 90 80 70 60 50 40 30 20 10 0 -10 -20
(ppm)

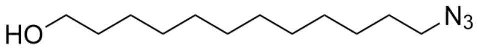

SM08

^{1}H NMR

400 mHz, $CDCl_3$

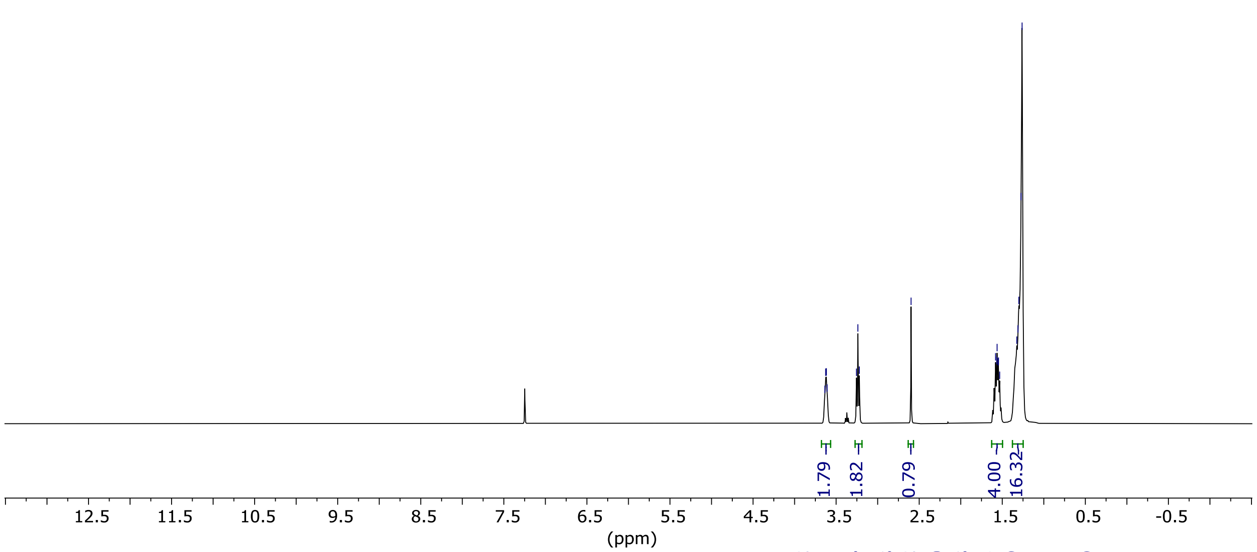

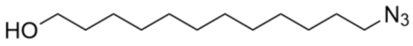

SM08

^{13}C NMR

100 mHz, $CDCl_3$

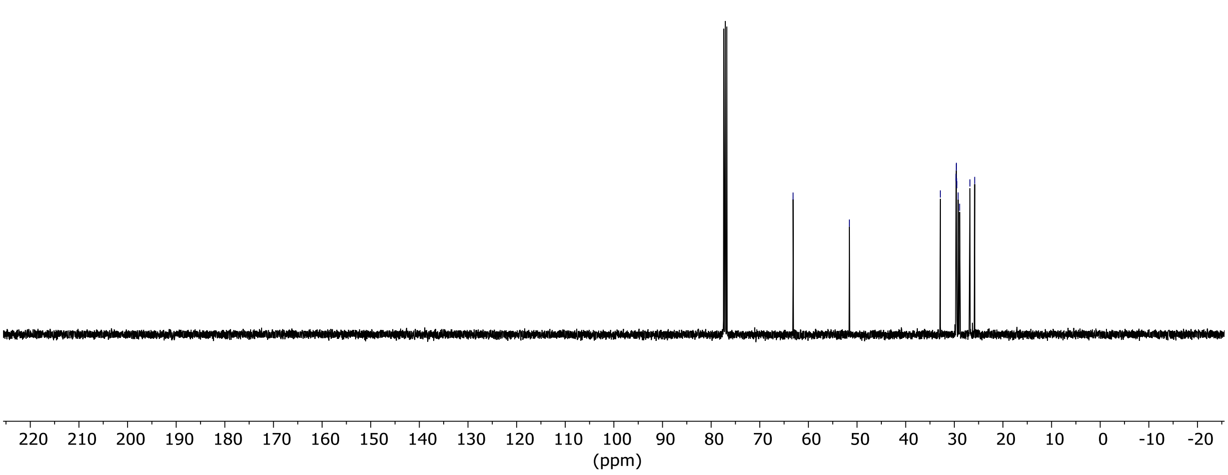

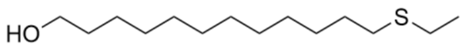

SM09

^{1}H NMR

500 mHz, $CDCl_3$

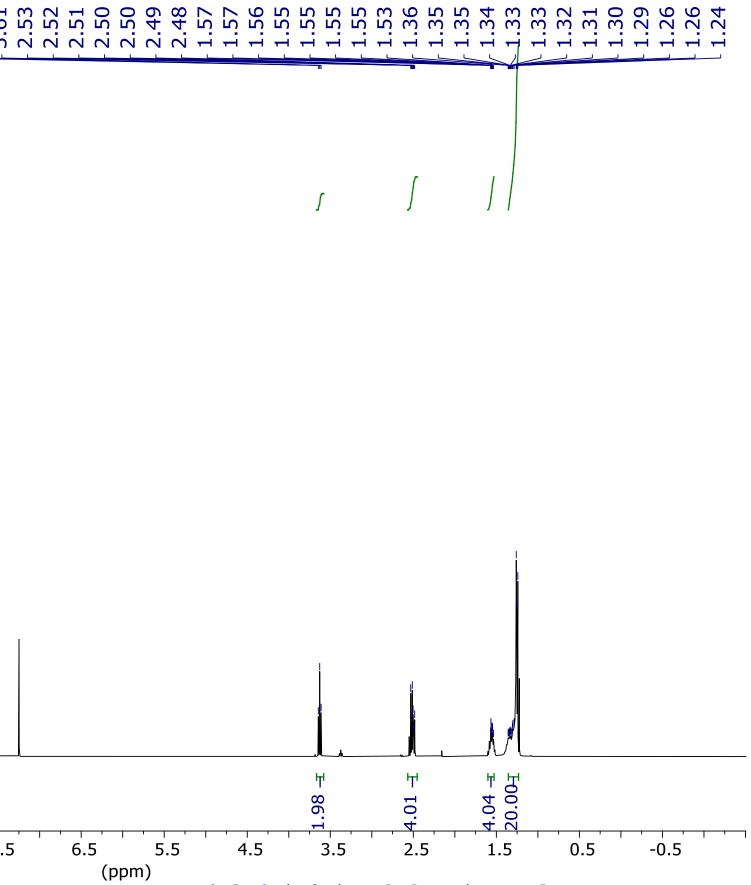

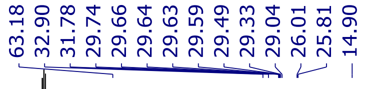

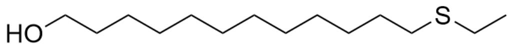

SM09

^{13}C NMR

126 mHz, $CDCl_3$

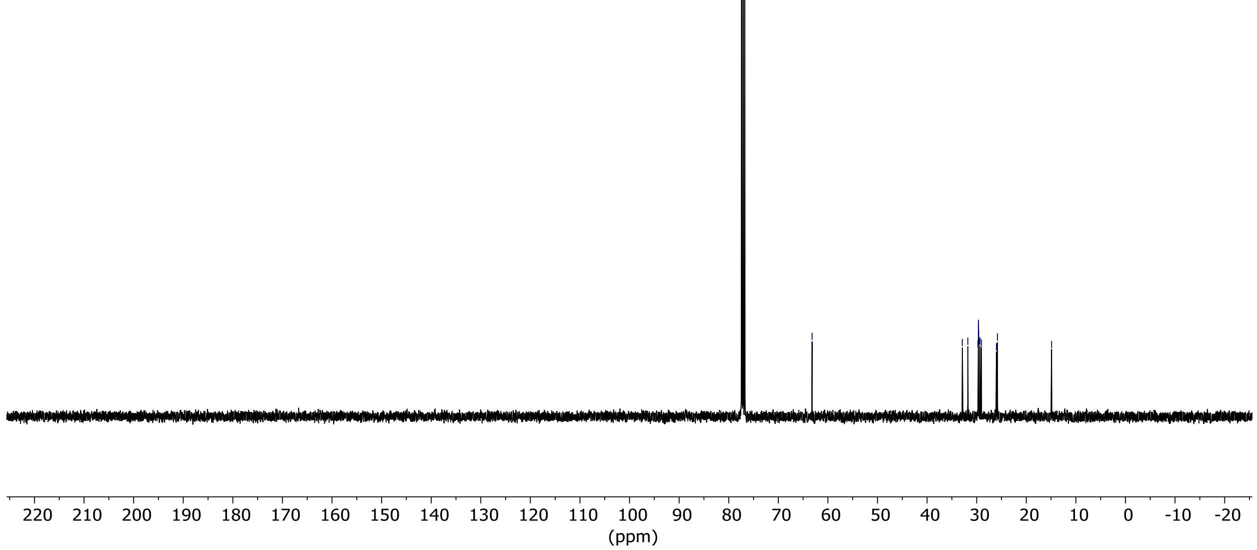

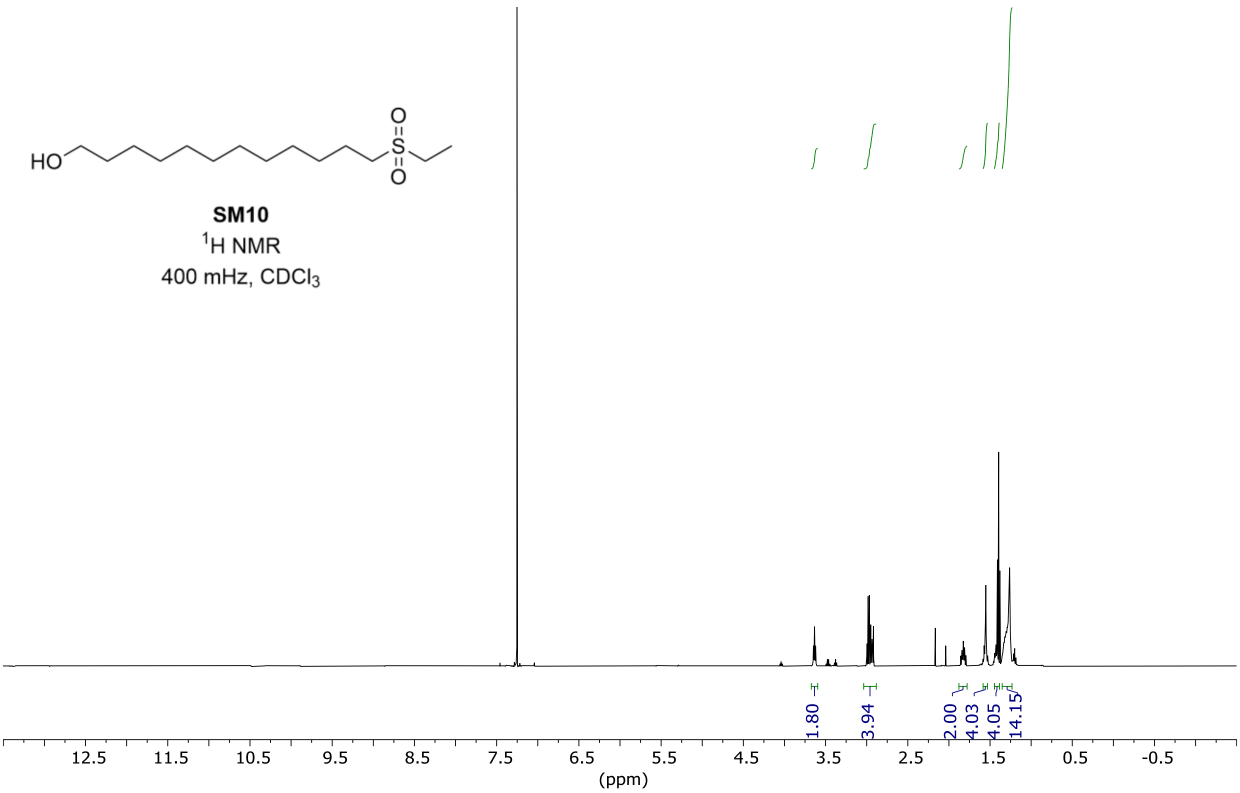
HO
O
S
O
SM10
1H NMR
400 mHz, CDCl3
1.80
3.94
2.00
4.03
4.05
14.15
12.5 11.5 10.5 9.5 8.5 7.5 6.5 5.5 4.5 3.5 2.5 1.5 0.5 -0.5
(ppm)

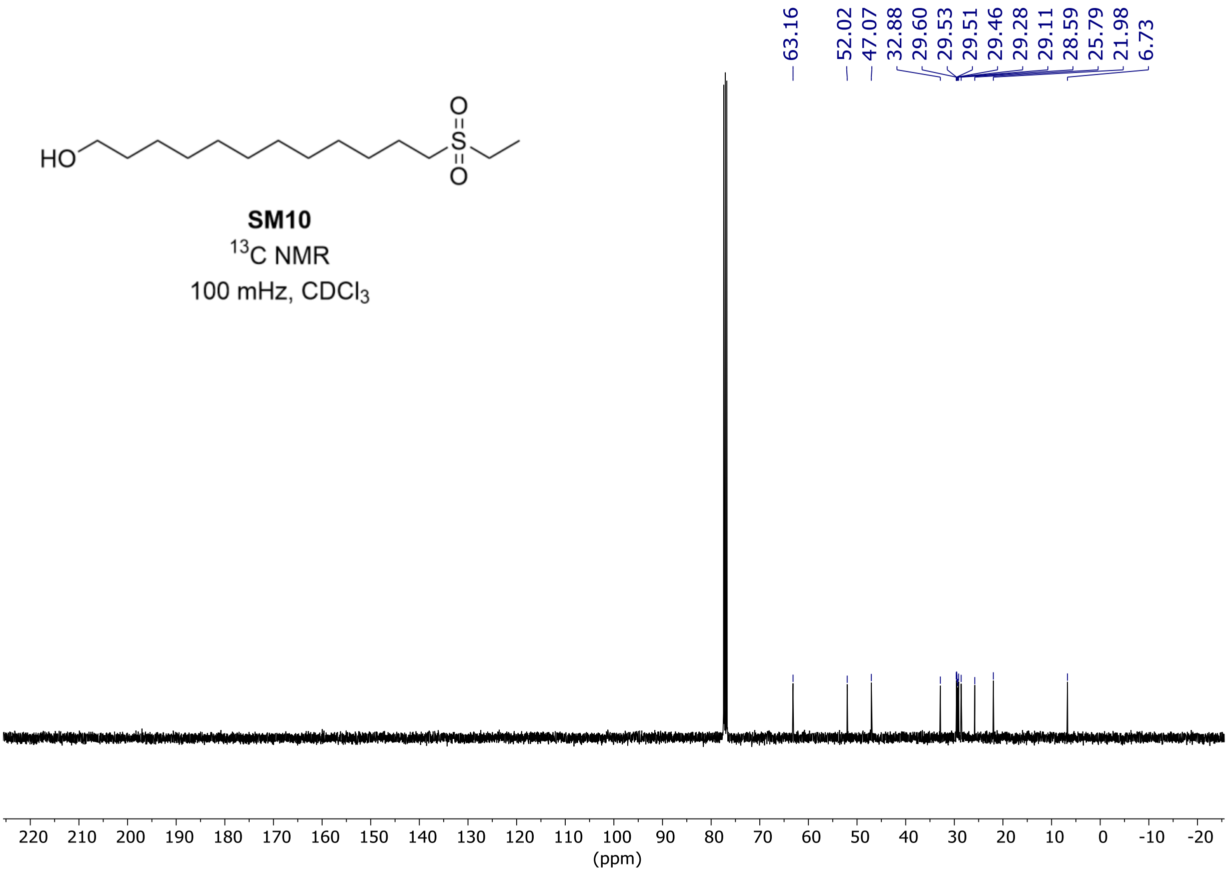
63.16
52.02
47.07
32.88
29.60
29.53
29.51
29.46
29.28
29.11
28.59
25.79
21.98
6.73
HO
O
S
O
SM10
13C NMR
100 mHz, CDCl3
220 210 200 190 180 170 160 150 140 130 120 110 100 90 80 70 60 50 40 30 20 10 0 -10 -20
(ppm)

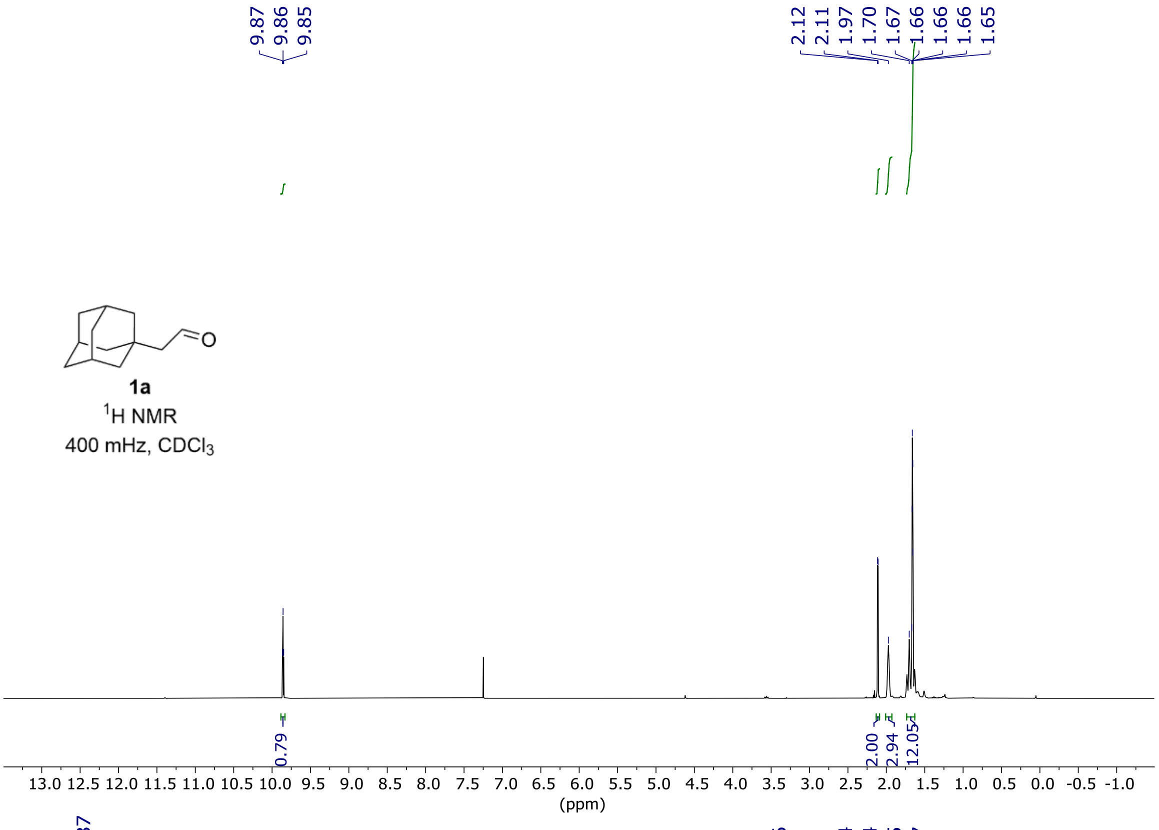
9.87
9.86
9.85
2.12
2.11
1.97
1.70
1.67
1.66
1.66
1.66
1.66
1.65
O
1a
^{1}H NMR
400 mHz, CDCl$_3$
0.79
2.00
2.94
12.05
13.0 12.5 12.0 11.5 11.0 10.5 10.0 9.5 9.0 8.5 8.0 7.5 7.0 6.5 6.0 5.5 5.0 4.5 4.0 3.5 3.0 2.5 2.0 1.5 1.0 0.5 0.0 -0.5 -1.0
(ppm)

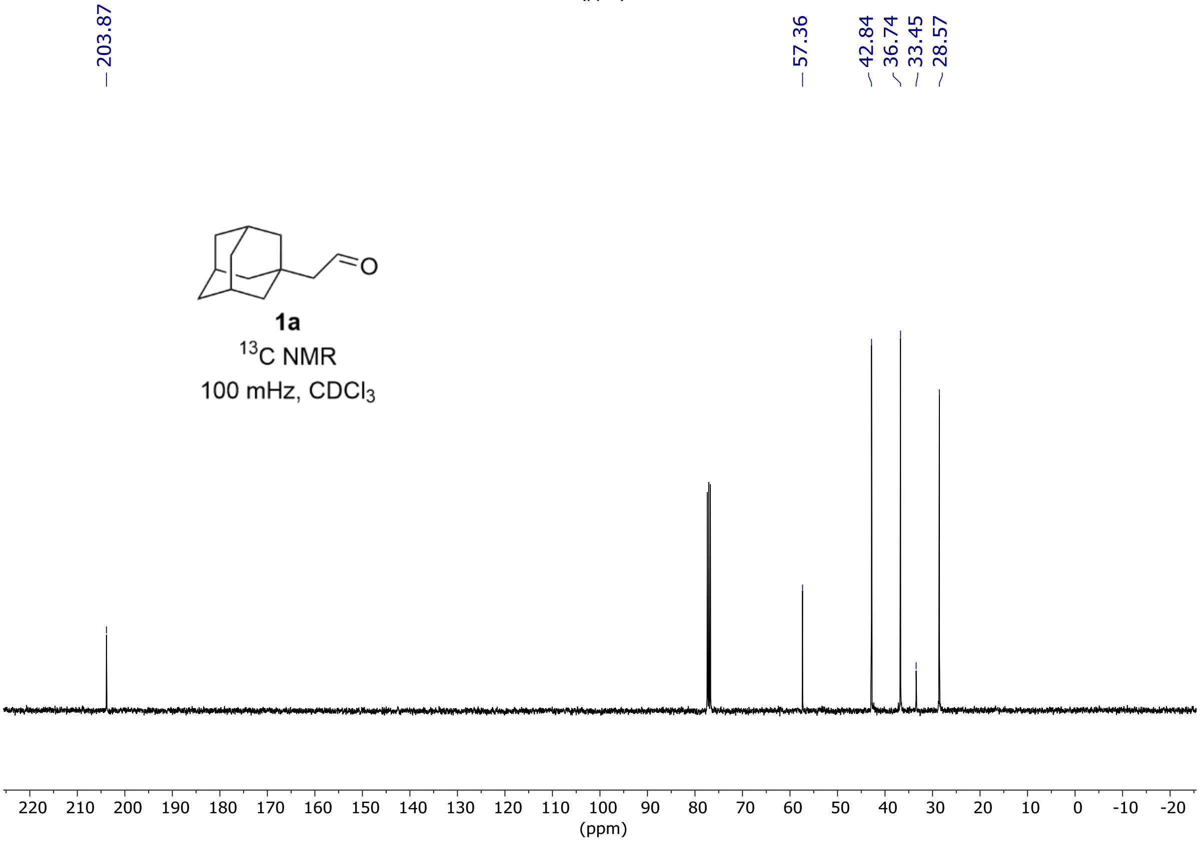
203.87
57.36
42.84
36.74
33.45
28.57
O
1a
^{13}C NMR
100 mHz, CDCl$_3$
220 210 200 190 180 170 160 150 140 130 120 110 100 90 80 70 60 50 40 30 20 10 0 -10 -20
(ppm)

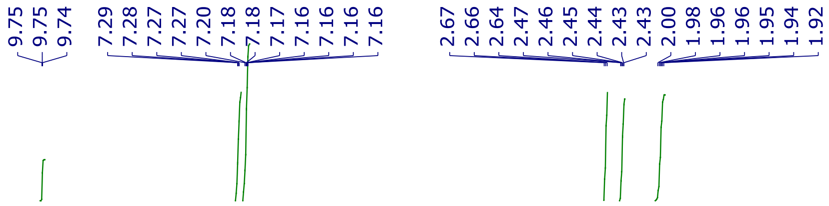

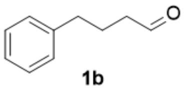

1b

^{1}H NMR

400 mHz, DMSO-d_6

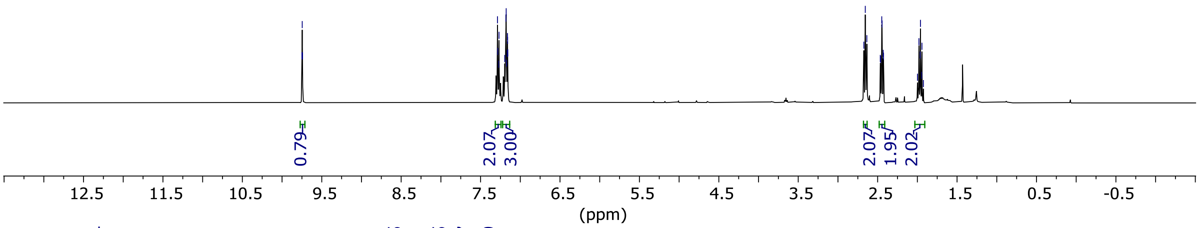

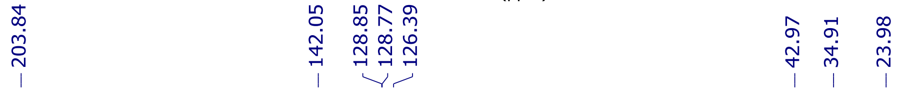

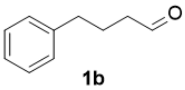

1b

^{13}C NMR

126 mHz, DMSO-d_6

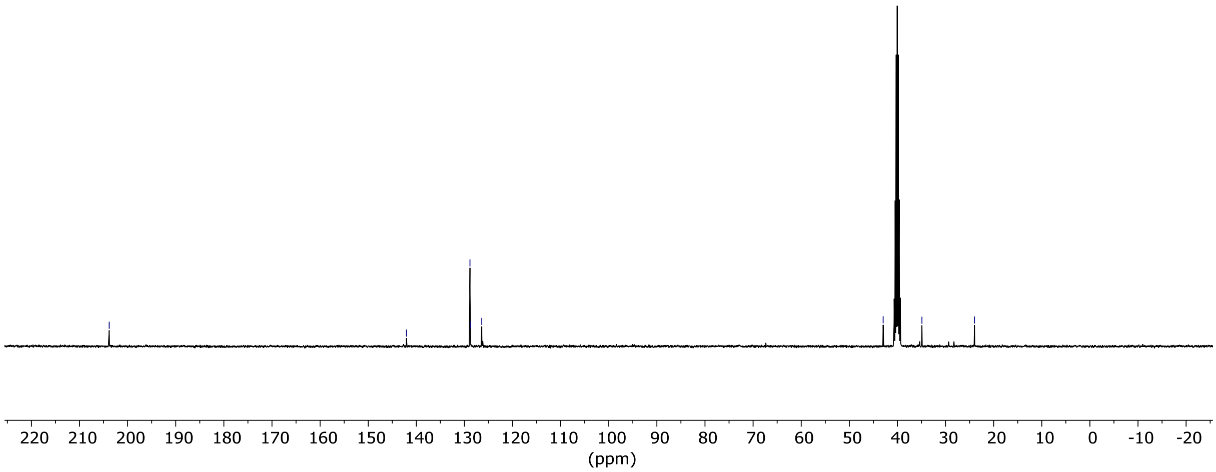

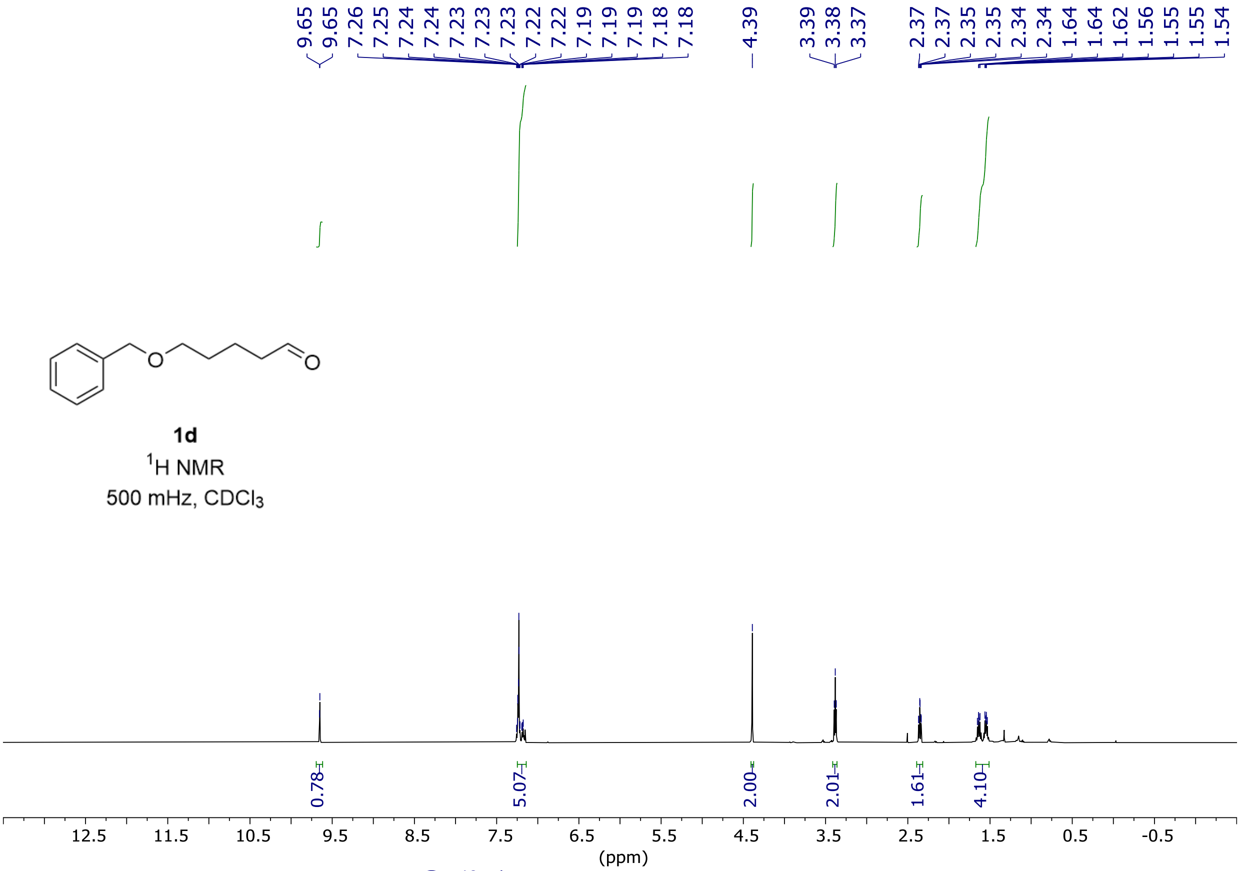

1d

^{1}H NMR

500 mHz, $CDCl_3$

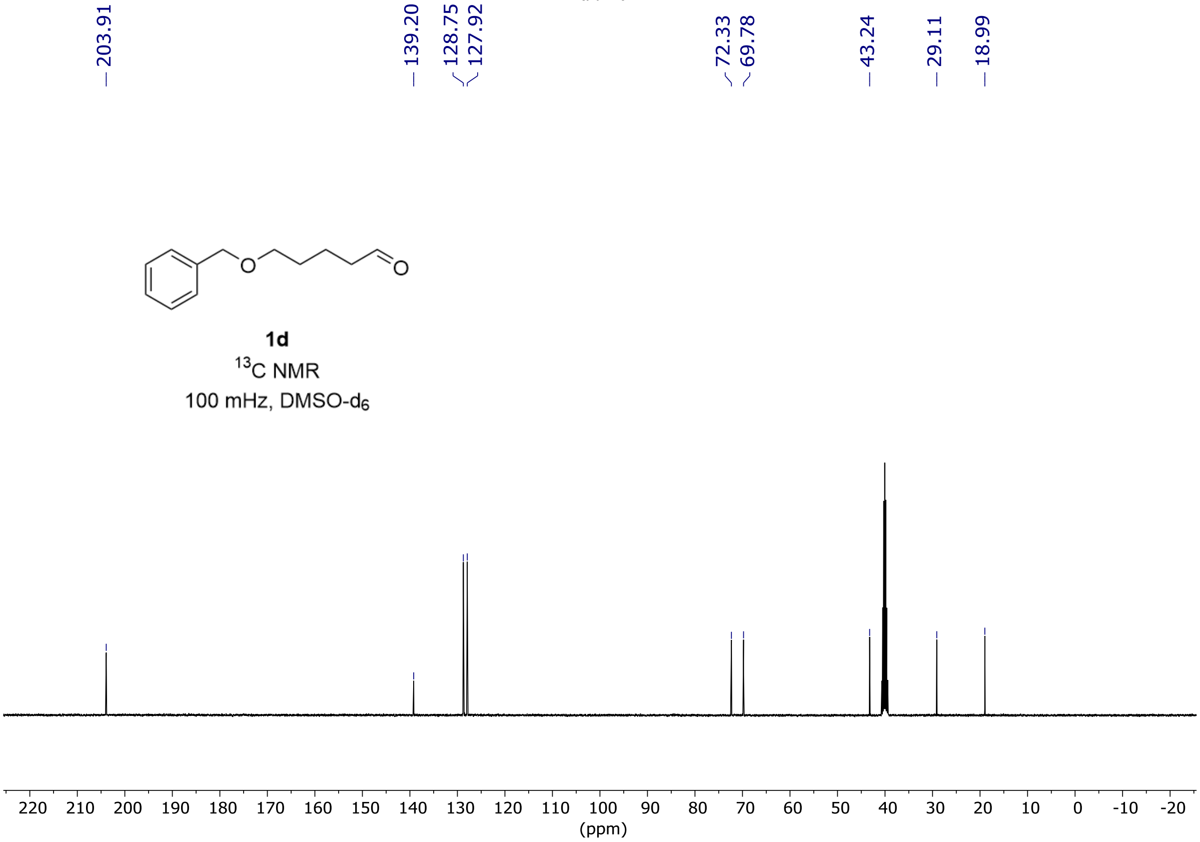

1d

^{13}C NMR

100 mHz, DMSO-d_6

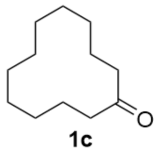

1c

^{1}H NMR

400 mHz, $CDCl_3$

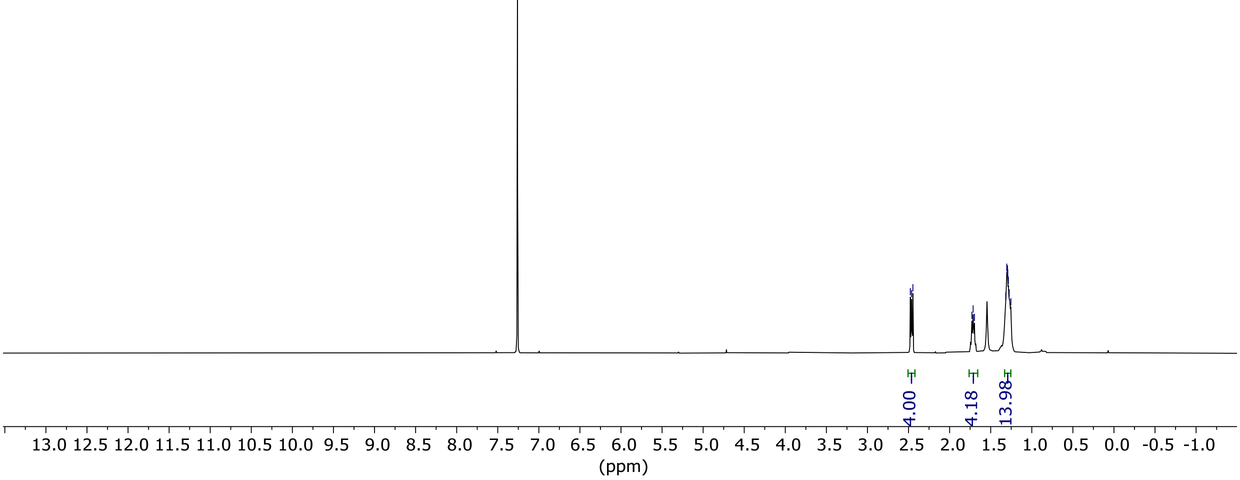

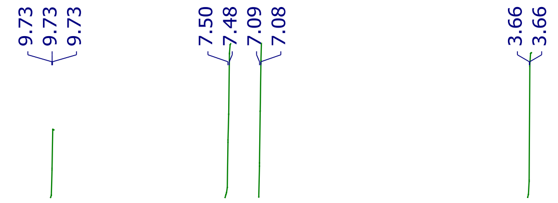

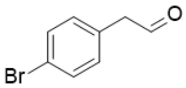

1e

^{1}H NMR

500 mHz, $CDCl_3$

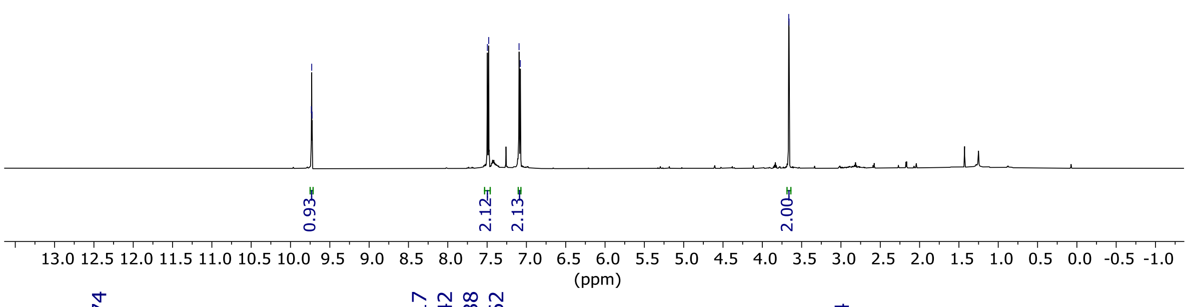

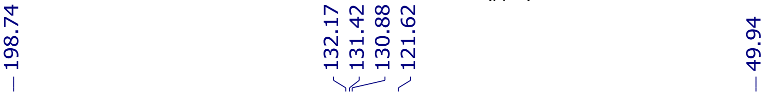

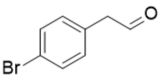

1e

^{13}C NMR

126 mHz, $CDCl_3$

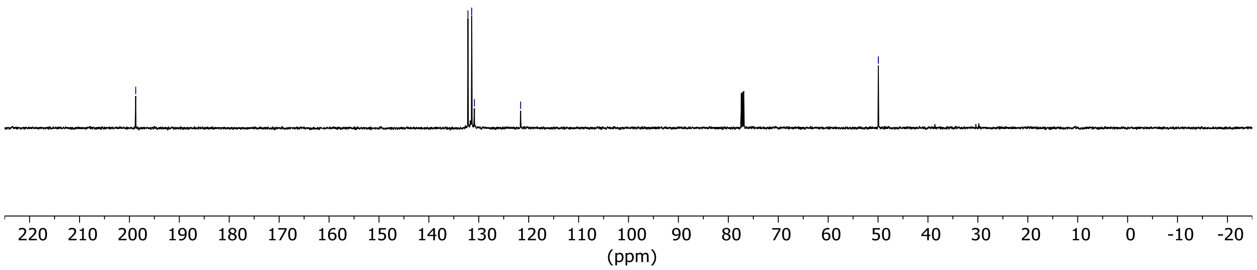

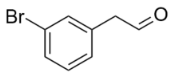

1f

^{1}H NMR

400 mHz, $CDCl_3$

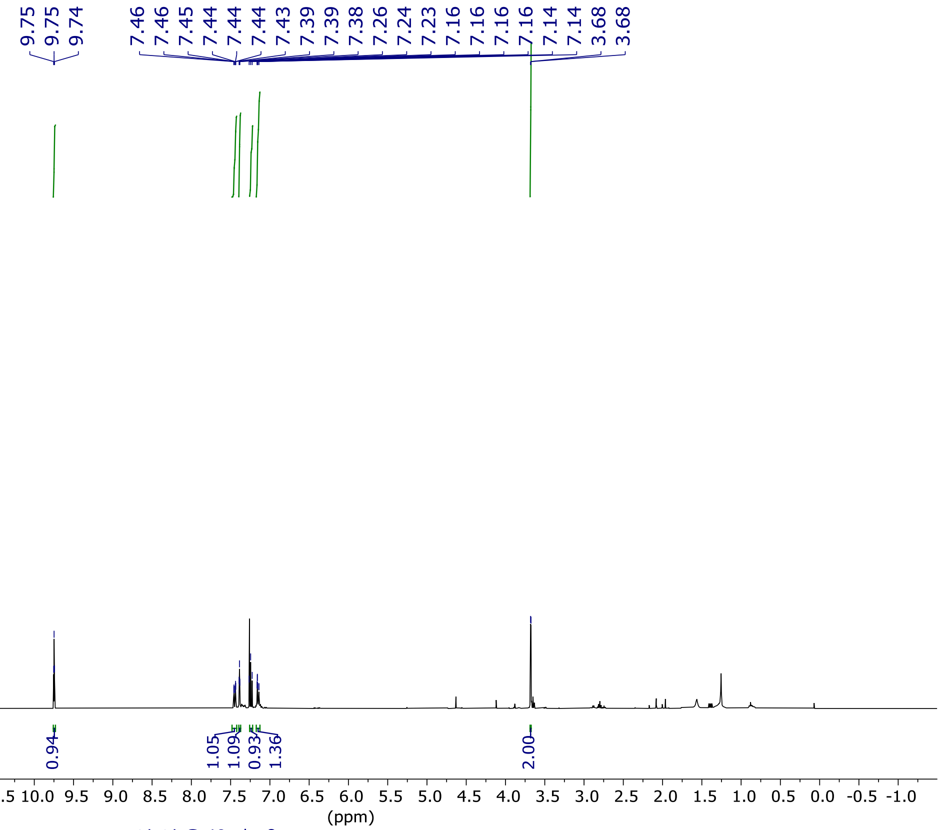

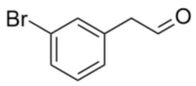

1f

^{13}C NMR

100 mHz, $CDCl_3$

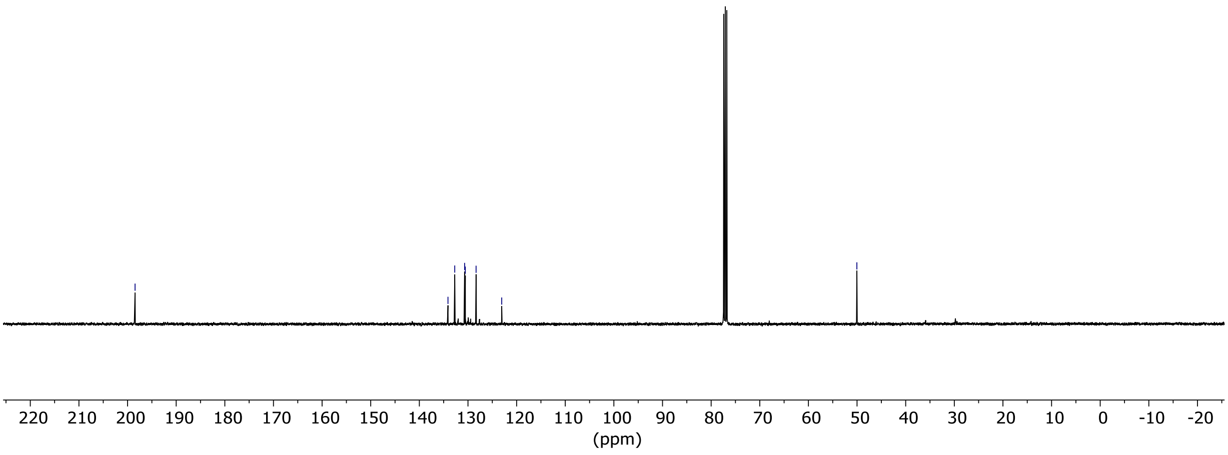

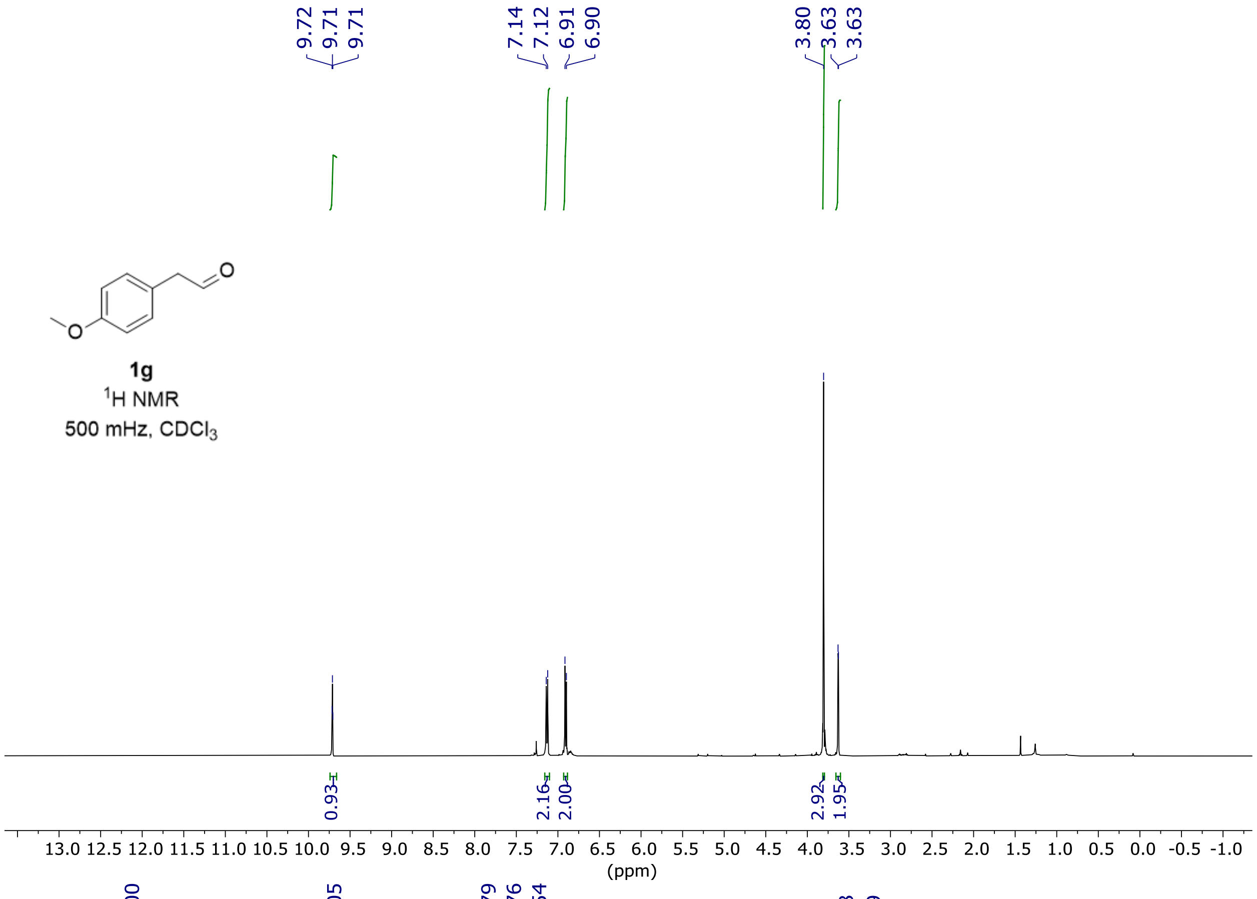
9.72
9.71
9.71
7.14
7.12
6.91
6.90
3.80
3.63
3.63
O
O
1g
1H NMR
500 mHz, CDCl3
0.93
2.16
2.00
2.92
1.95
13.0 12.5 12.0 11.5 11.0 10.5 10.0 9.5 9.0 8.5 8.0 7.5 7.0 6.5 6.0 5.5 5.0 4.5 4.0 3.5 3.0 2.5 2.0 1.5 1.0 0.5 0.0 -0.5 -1.0
(ppm)

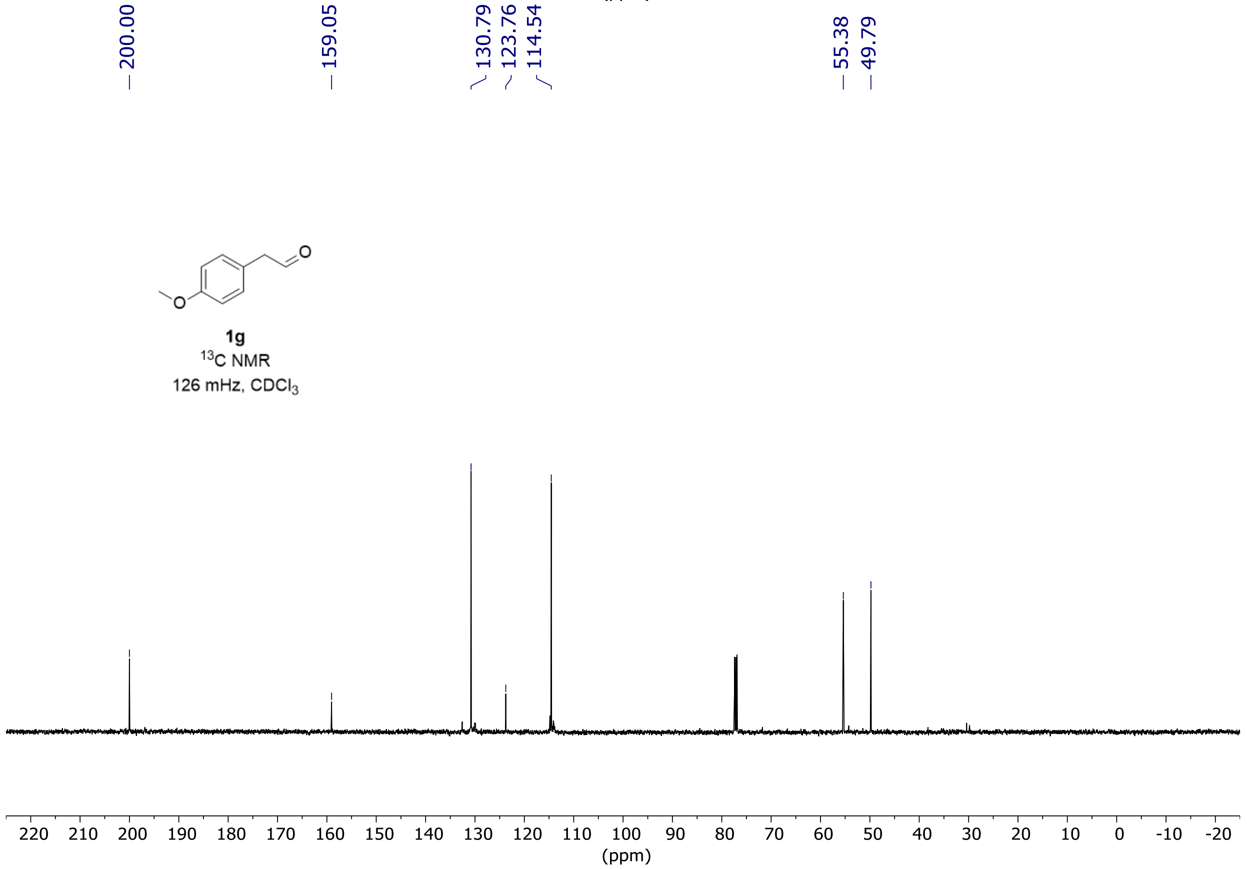
200.00
159.05
130.79
123.76
114.54
55.38
49.79
O
O
1g
13C NMR
126 mHz, CDCl3
220 210 200 190 180 170 160 150 140 130 120 110 100 90 80 70 60 50 40 30 20 10 0 -10 -20
(ppm)

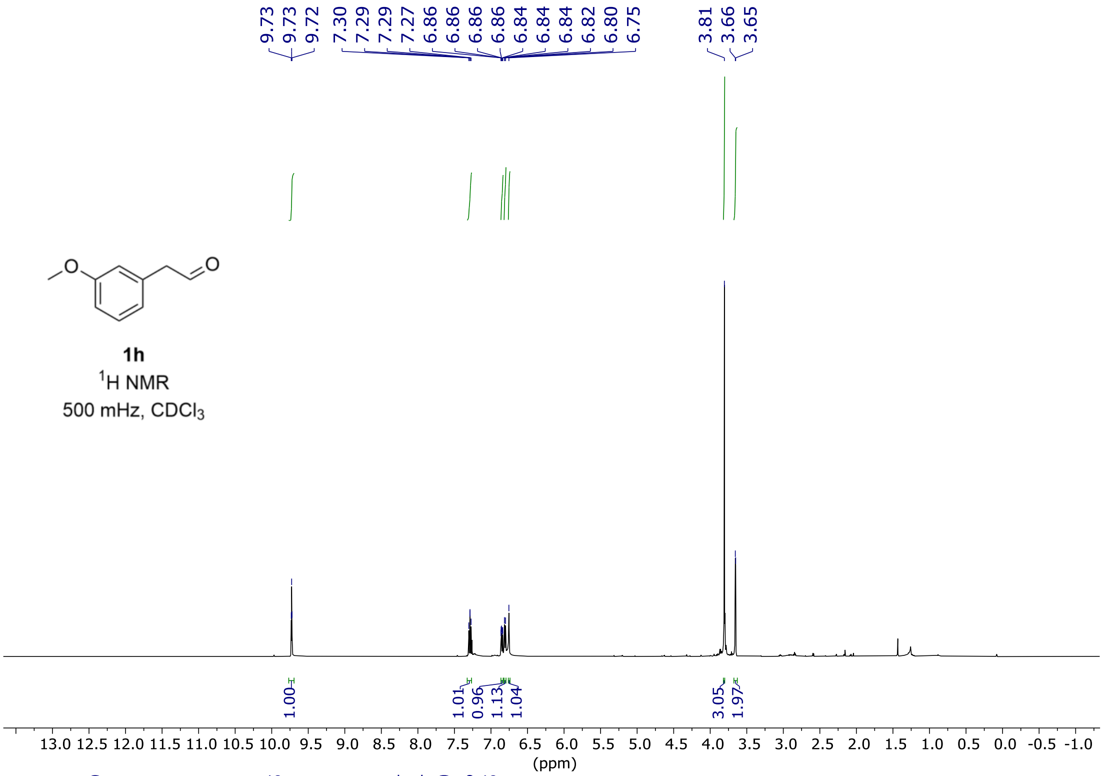

1h
^{1}H NMR
500 mHz, CDCl$_3$
9.73
9.73
9.72
7.30
7.29
7.29
7.27
6.86
6.86
6.86
6.86
6.84
6.84
6.84
6.82
6.80
6.75
3.81
3.66
3.65
1.00
1.01
0.96
1.13
1.04
3.05
1.97
13.0 12.5 12.0 11.5 11.0 10.5 10.0 9.5 9.0 8.5 8.0 7.5 7.0 6.5 6.0 5.5 5.0 4.5 4.0 3.5 3.0 2.5 2.0 1.5 1.0 0.5 0.0 -0.5 -1.0
(ppm)

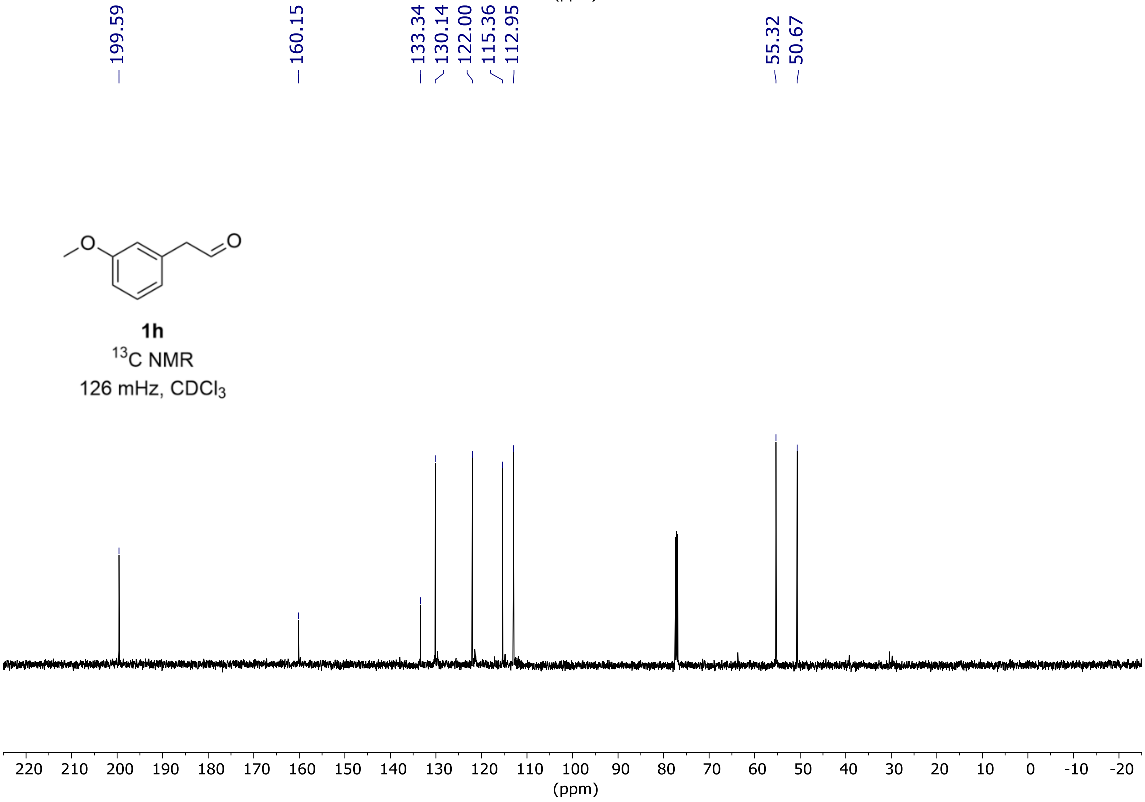

199.59
160.15
133.34
130.14
122.00
115.36
112.95
55.32
50.67
1h
^{13}C NMR
126 mHz, CDCl$_3$
220 210 200 190 180 170 160 150 140 130 120 110 100 90 80 70 60 50 40 30 20 10 0 -10 -20
(ppm)

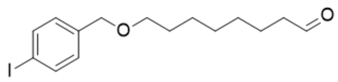

1i

^{1}H NMR

500 mHz, $CDCl_3$

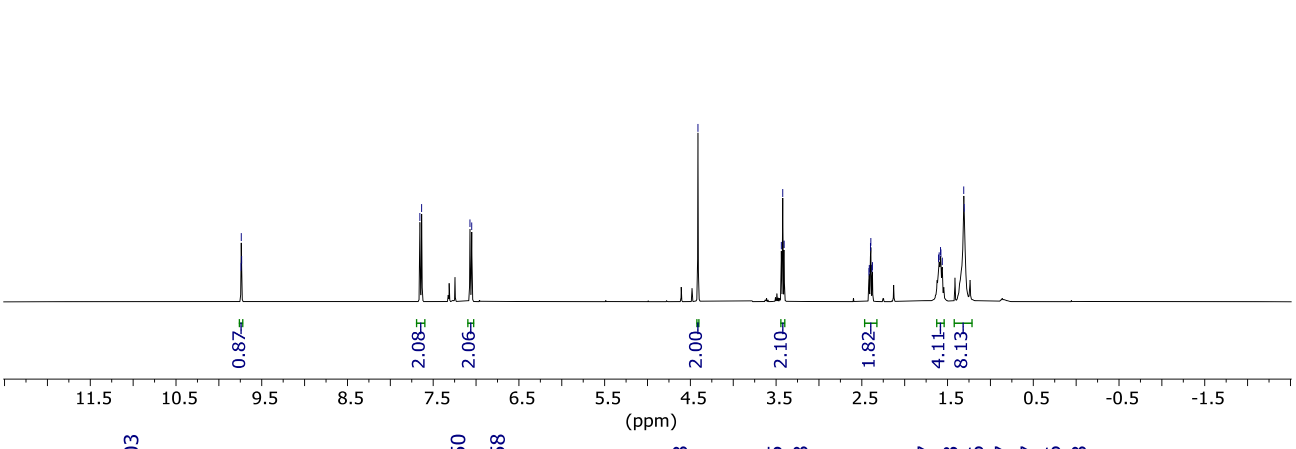

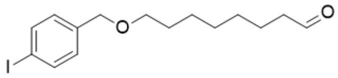

1i

^{13}C NMR

126 mHz, $CDCl_3$

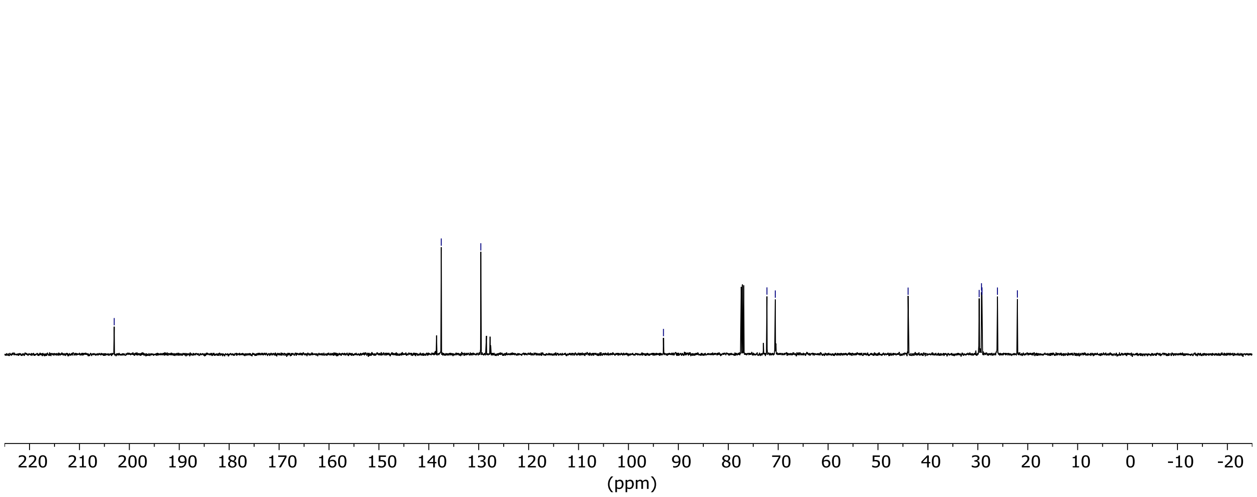

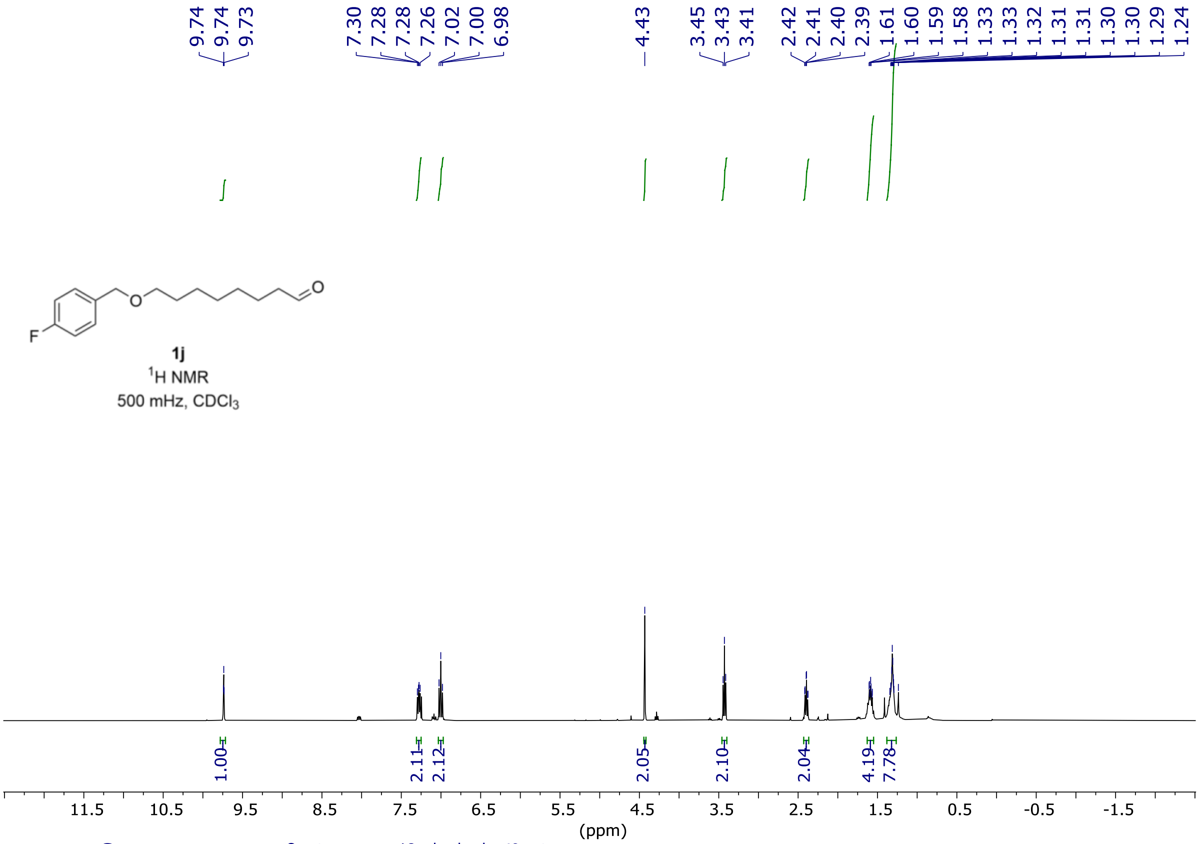
1j
^{1}H NMR
500 mHz, $CDCl_3$
(ppm)

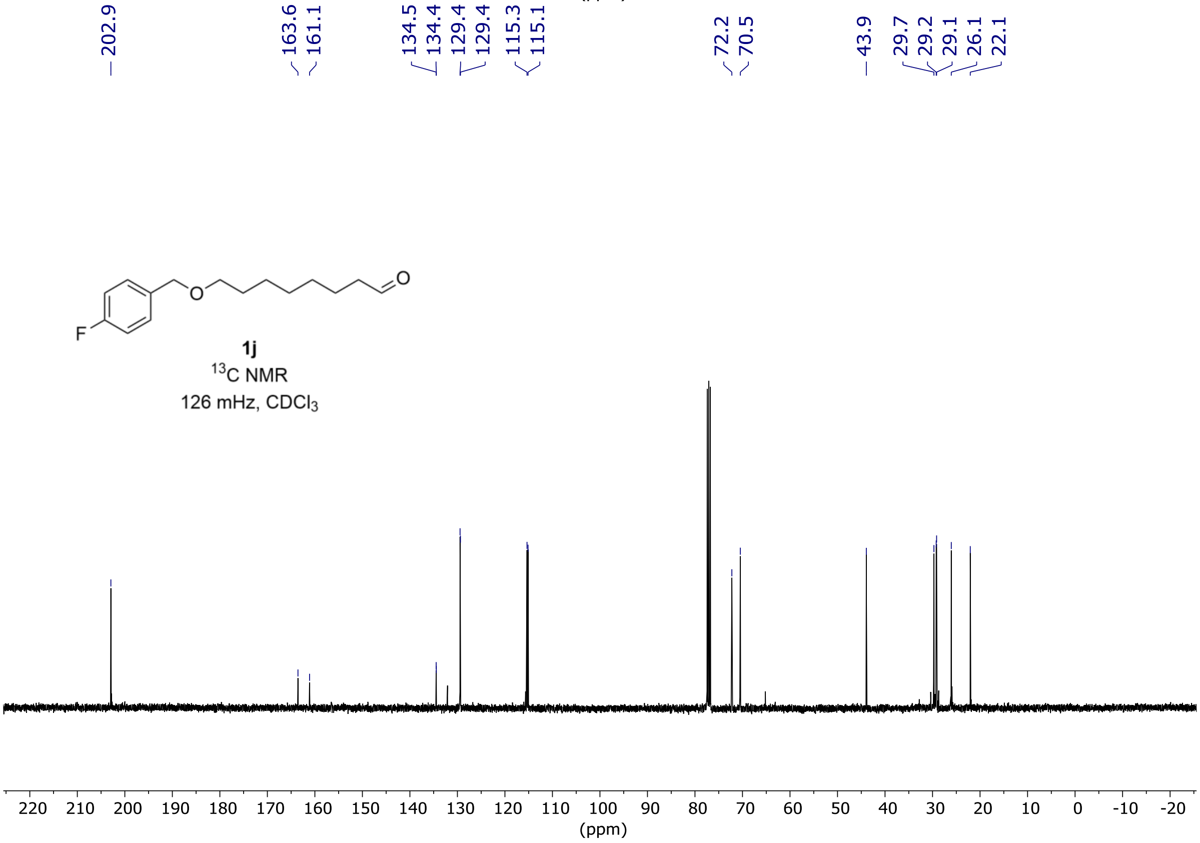
1j
^{13}C NMR
126 mHz, $CDCl_3$
(ppm)

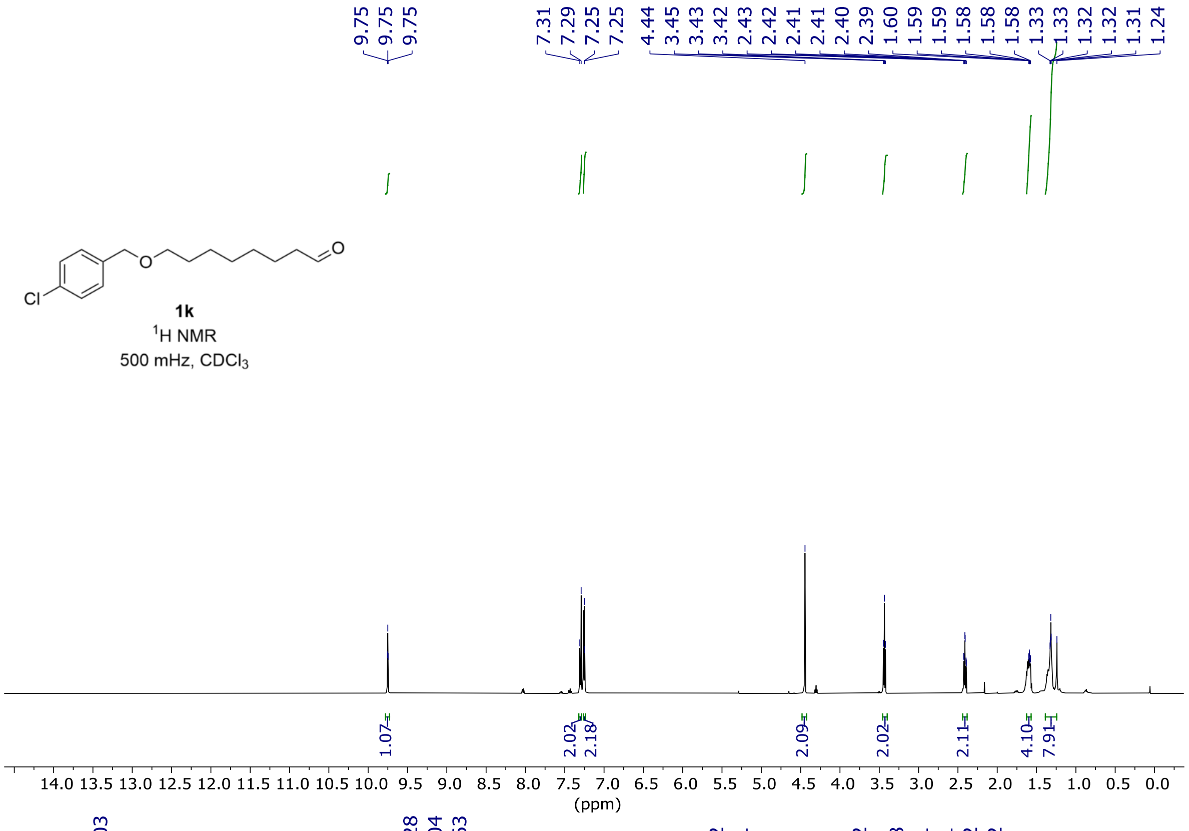
9.75
9.75
9.75
7.31
7.29
7.25
7.25
4.44
3.45
3.43
3.42
2.43
2.42
2.41
2.41
2.40
2.39
1.60
1.59
1.59
1.58
1.58
1.58
1.33
1.33
1.32
1.32
1.31
1.24
Cl
O
1k
1H NMR
500 mHz, CDCl3
1.07
2.02
2.18
2.09
2.02
2.11
4.10
7.91
14.0 13.5 13.0 12.5 12.0 11.5 11.0 10.5 10.0 9.5 9.0 8.5 8.0 7.5 7.0 6.5 6.0 5.5 5.0 4.5 4.0 3.5 3.0 2.5 2.0 1.5 1.0 0.5 0.0
(ppm)

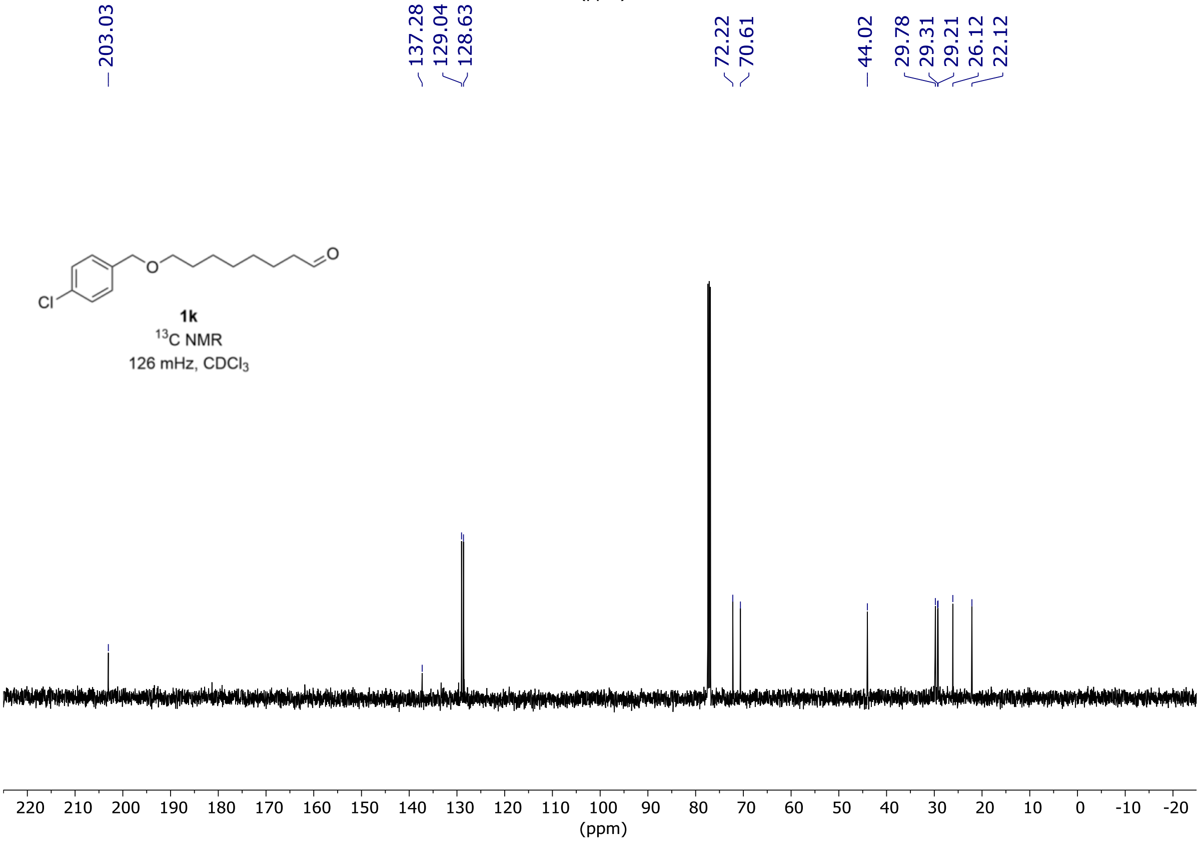
203.03
137.28
129.04
128.63
72.22
70.61
44.02
29.78
29.31
29.21
26.12
22.12
Cl
O
1k
13C NMR
126 mHz, CDCl3
220 210 200 190 180 170 160 150 140 130 120 110 100 90 80 70 60 50 40 30 20 10 0 -10 -20
(ppm)

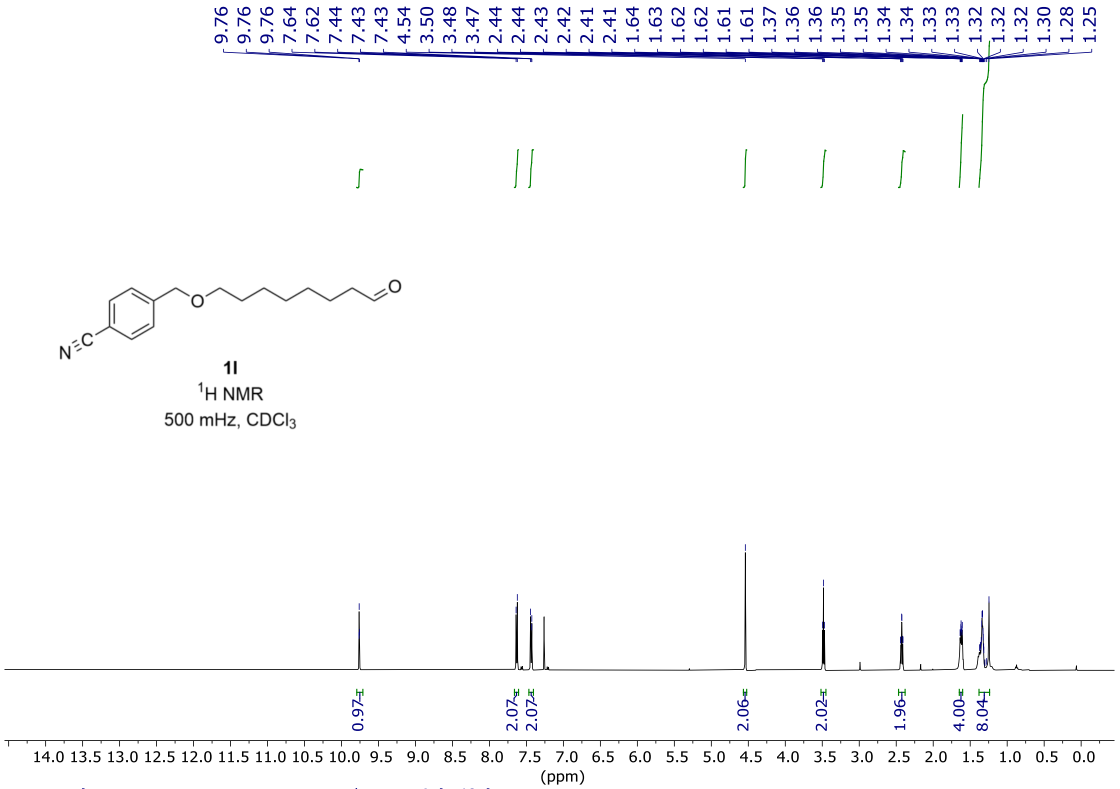

1l

^{1}H NMR

500 mHz, $CDCl_3$

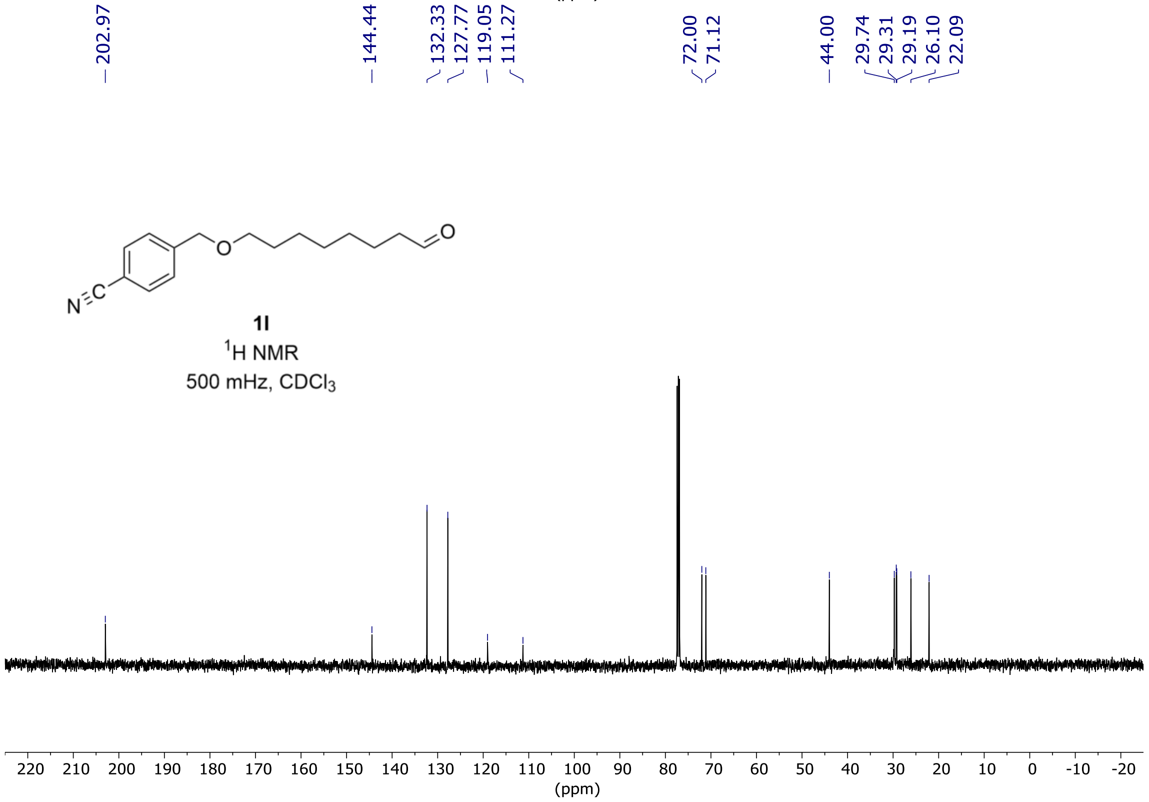

1l

^{1}H NMR

500 mHz, $CDCl_3$

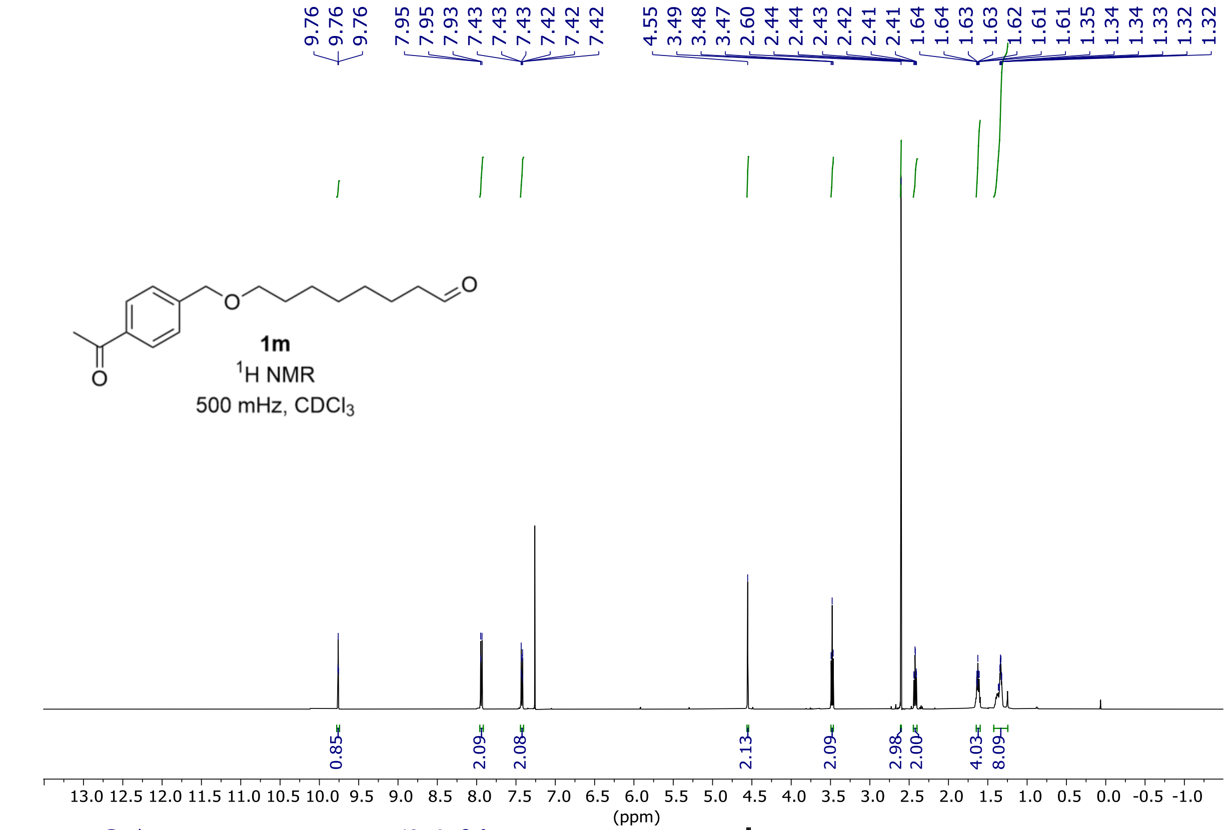
9.76 9.76 9.76 7.95 7.95 7.93 7.43 7.43 7.43 7.42 7.42 7.42 4.55 3.49 3.48 3.47 2.60 2.44 2.44 2.43 2.42 2.41 2.41 1.64 1.64 1.63 1.63 1.62 1.61 1.61 1.35 1.34 1.34 1.33 1.32 1.32
1m
^{1}H NMR
500 mHz, $CDCl_3$
0.85 2.09 2.08 2.13 2.09 2.98 2.00 4.03 8.09
13.0 12.5 12.0 11.5 11.0 10.5 10.0 9.5 9.0 8.5 8.0 7.5 7.0 6.5 6.0 5.5 5.0 4.5 4.0 3.5 3.0 2.5 2.0 1.5 1.0 0.5 0.0 -0.5 -1.0
(ppm)

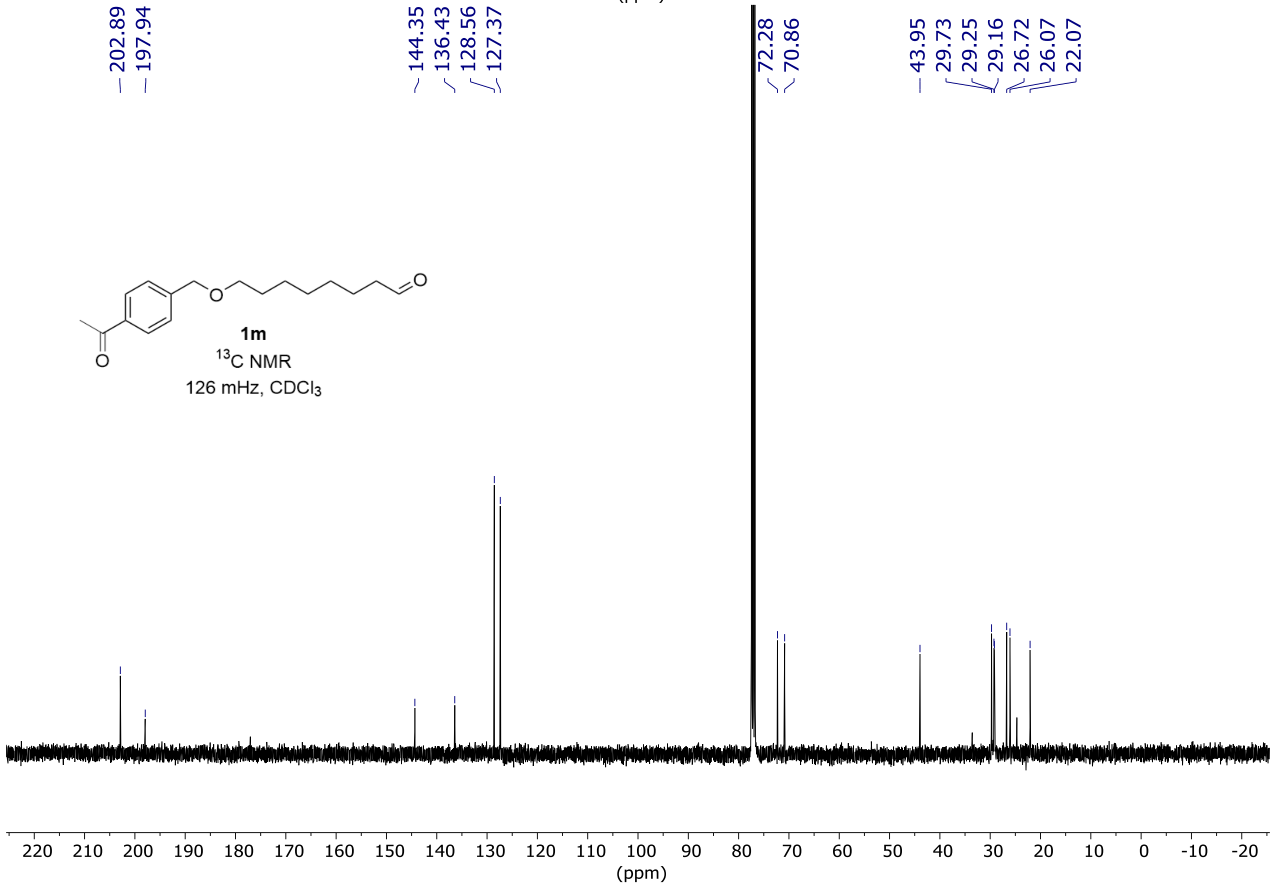
202.89 197.94 144.35 136.43 128.56 127.37 72.28 70.86 43.95 29.73 29.25 29.16 26.72 26.07 22.07
1m
^{13}C NMR
126 mHz, $CDCl_3$
220 210 200 190 180 170 160 150 140 130 120 110 100 90 80 70 60 50 40 30 20 10 0 -10 -20
(ppm)

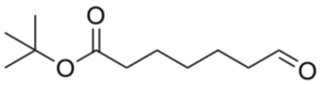

1n

^{1}H NMR

500 mHz, $CDCl_3$

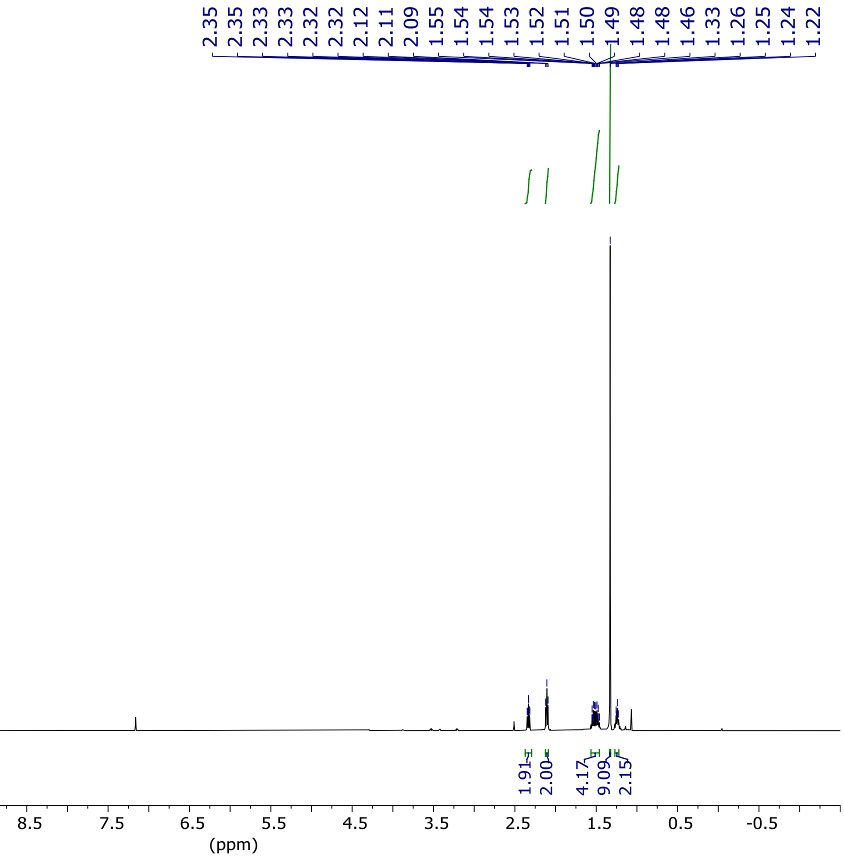

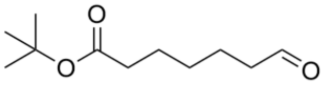

1n

^{13}C NMR

126 mHz, $CDCl_3$

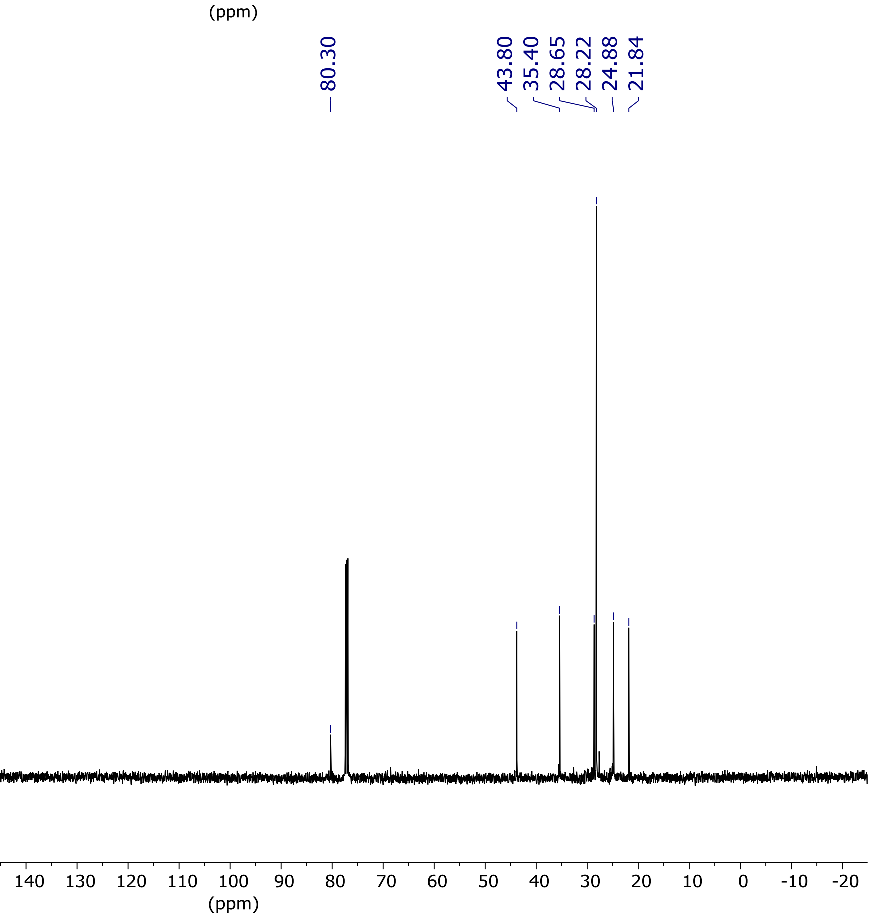

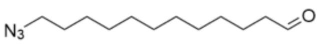

1o
^1H NMR
500 mHz, $CDCl_3$

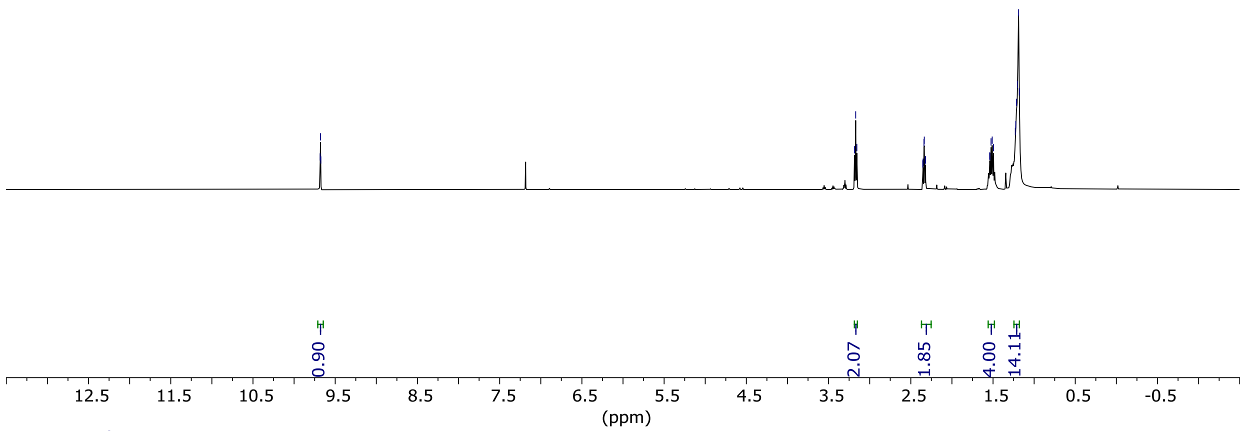

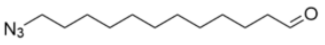

1o
^{13}C NMR
126 mHz, $CDCl_3$

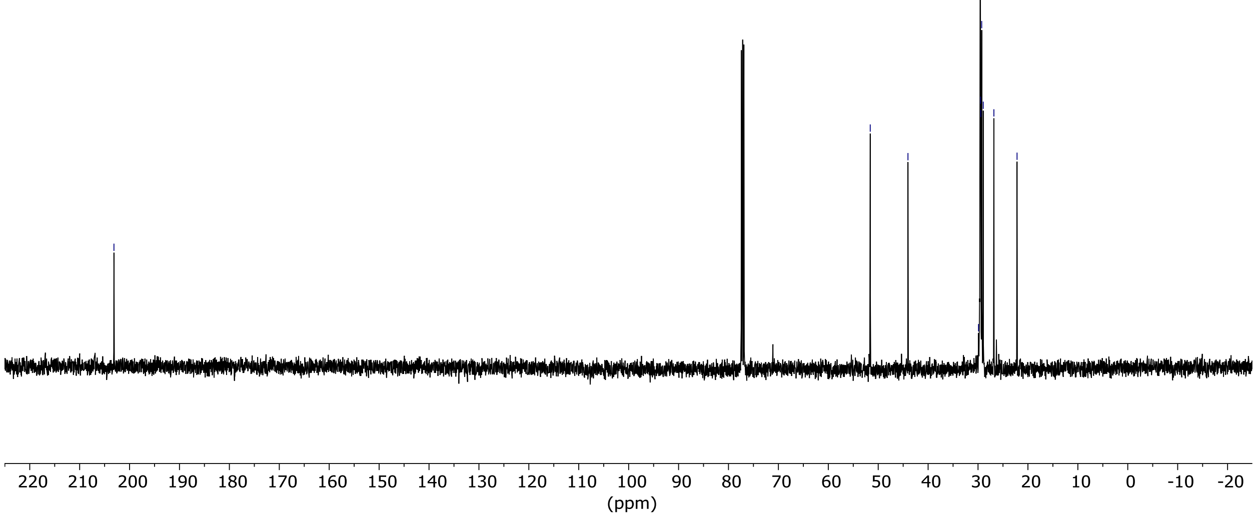

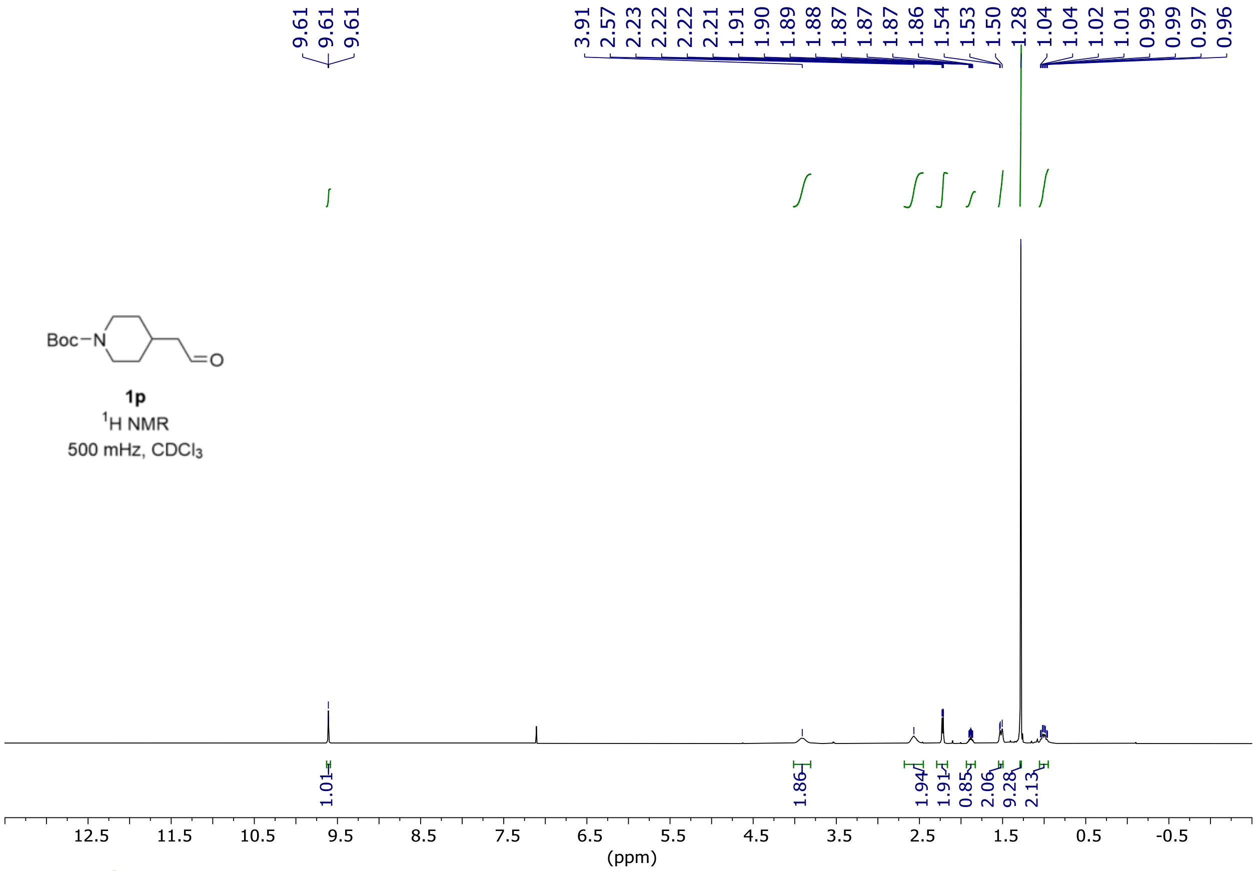
9.61
9.61
9.61
3.91
2.57
2.23
2.22
2.22
2.21
1.91
1.90
1.89
1.88
1.87
1.87
1.87
1.86
1.54
1.53
1.50
1.28
1.04
1.04
1.02
1.01
0.99
0.99
0.97
0.96
Boc
N
O
1p
^{1}H NMR
500 mHz, CDCl$_3$
1.01
1.86
1.94
1.91
0.85
2.06
9.28
2.13
12.5
11.5
10.5
9.5
8.5
7.5
6.5
5.5
4.5
3.5
2.5
1.5
0.5
-0.5
(ppm)

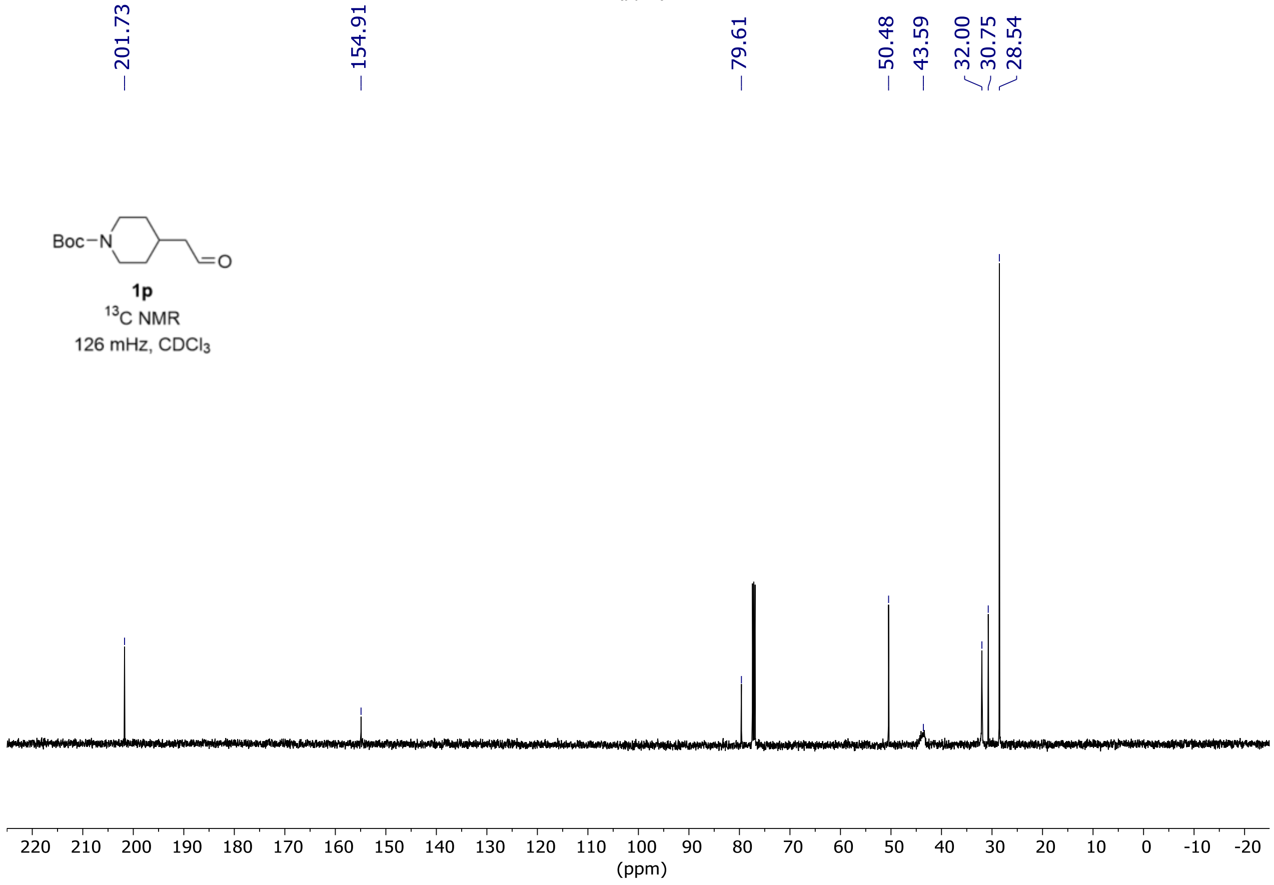
201.73
154.91
79.61
50.48
43.59
32.00
30.75
28.54
Boc
N
O
1p
^{13}C NMR
126 mHz, CDCl$_3$
220
210
200
190
180
170
160
150
140
130
120
110
100
90
80
70
60
50
40
30
20
10
0
-10
-20
(ppm)

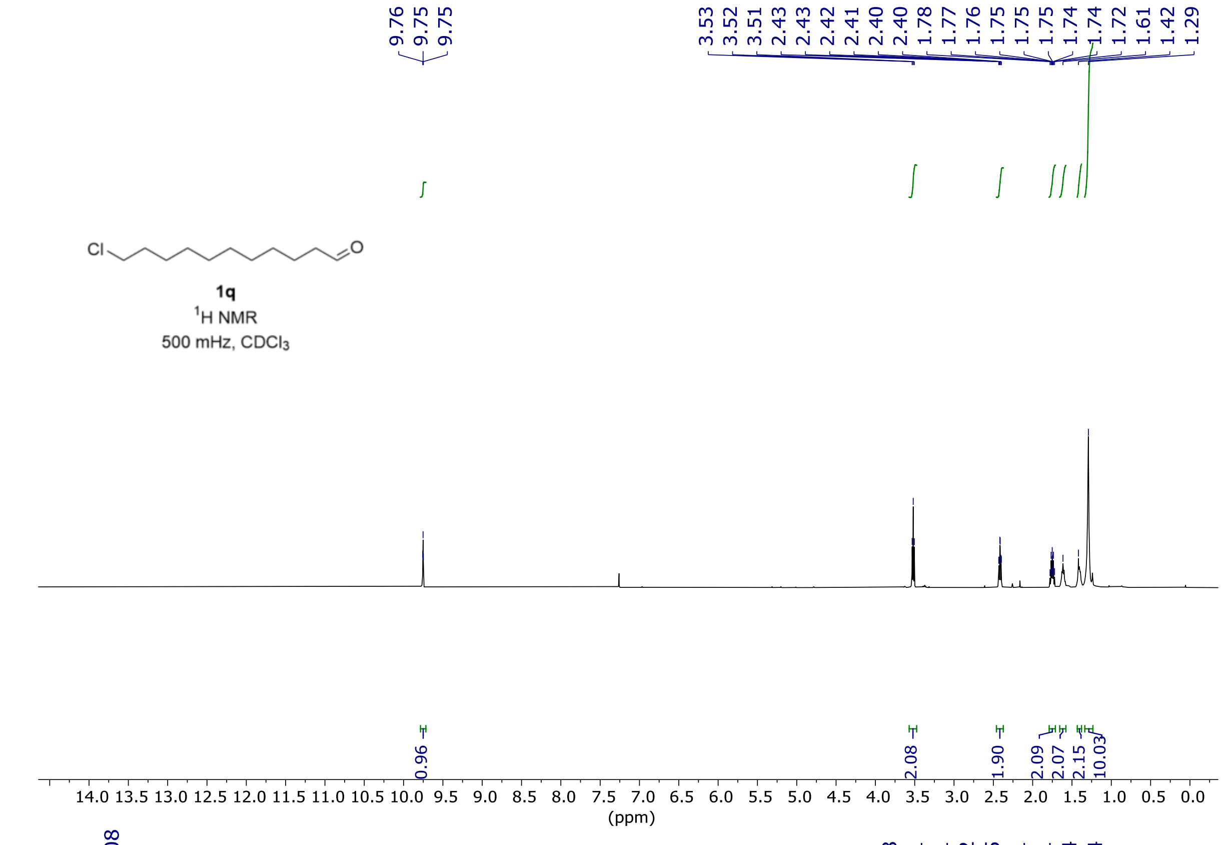
9.76
9.75
9.75
3.53
3.52
3.51
2.43
2.43
2.42
2.41
2.40
2.40
1.78
1.77
1.76
1.75
1.75
1.75
1.74
1.74
1.72
1.61
1.42
1.29
Cl
O
1q
1H NMR
500 mHz, CDCl3
0.96
2.08
1.90
2.09
2.07
2.15
10.03
14.0 13.5 13.0 12.5 12.0 11.5 11.0 10.5 10.0 9.5 9.0 8.5 8.0 7.5 7.0 6.5 6.0 5.5 5.0 4.5 4.0 3.5 3.0 2.5 2.0 1.5 1.0 0.5 0.0
(ppm)

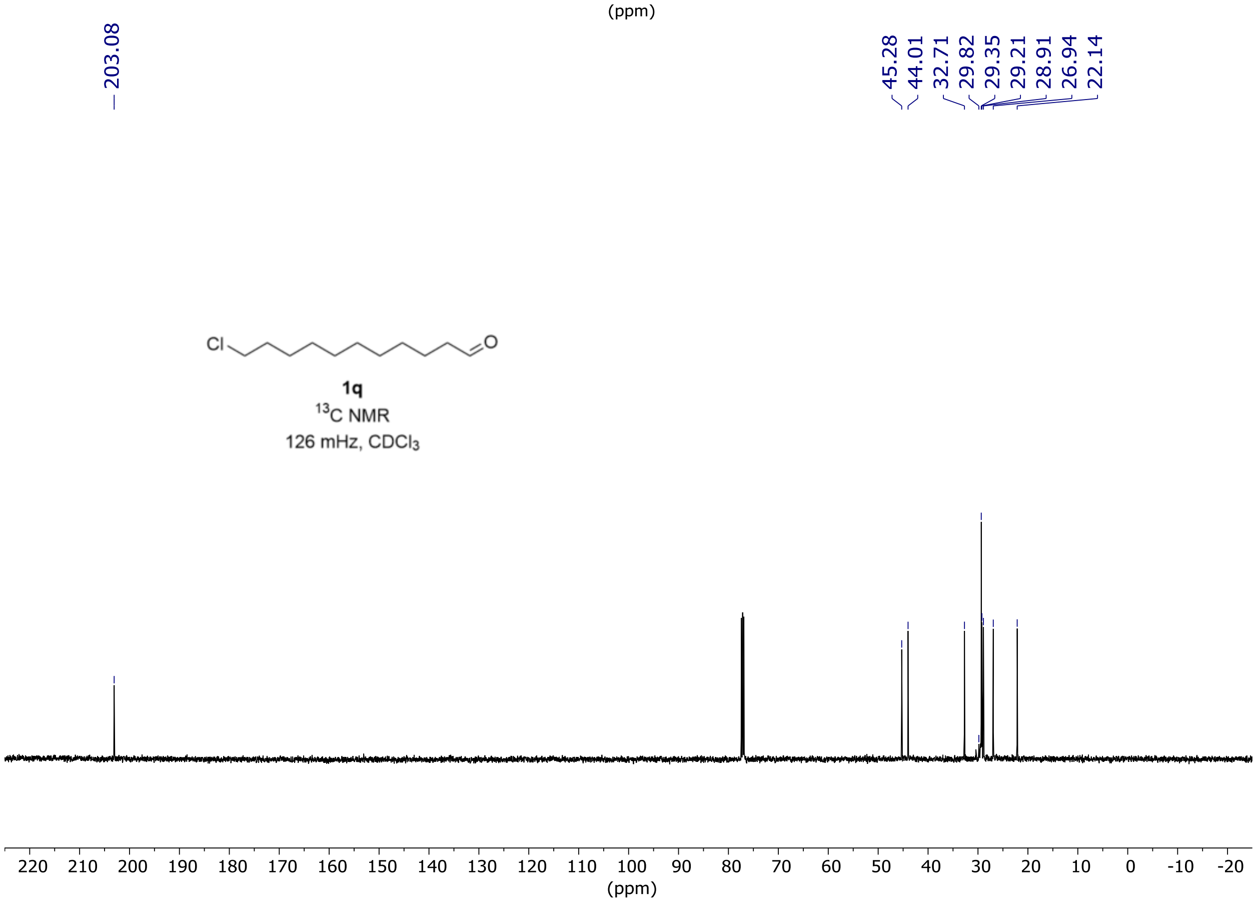
203.08
45.28
44.01
32.71
29.82
29.35
29.21
28.91
26.94
22.14
Cl
O
1q
13C NMR
126 mHz, CDCl3
220 210 200 190 180 170 160 150 140 130 120 110 100 90 80 70 60 50 40 30 20 10 0 -10 -20
(ppm)

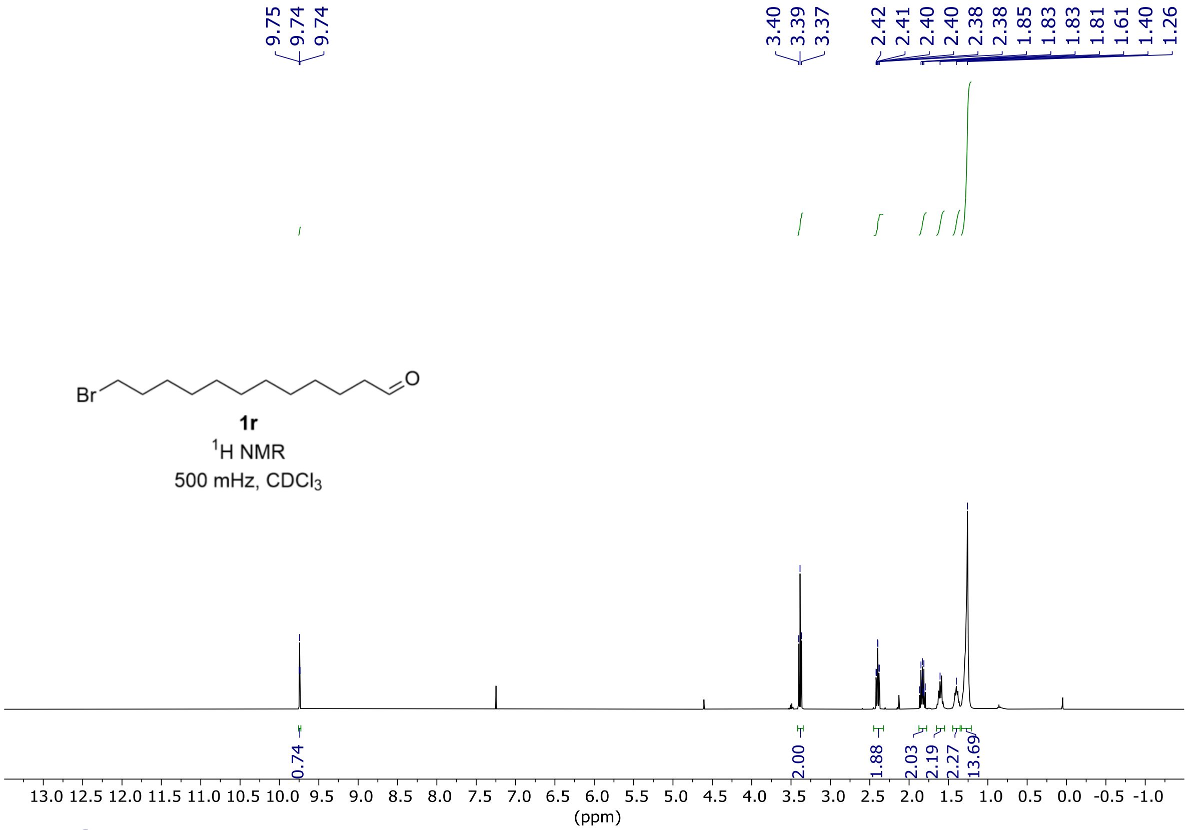
9.75
9.74
9.74
3.40
3.39
3.37
2.42
2.41
2.40
2.40
2.38
2.38
1.85
1.83
1.83
1.81
1.61
1.40
1.26
Br
O
1r
^{1}H NMR
500 mHz, $CDCl_3$
0.74
2.00
1.88
2.03
2.19
2.27
13.69
13.0 12.5 12.0 11.5 11.0 10.5 10.0 9.5 9.0 8.5 8.0 7.5 7.0 6.5 6.0 5.5 5.0 4.5 4.0 3.5 3.0 2.5 2.0 1.5 1.0 0.5 0.0 -0.5 -1.0
(ppm)

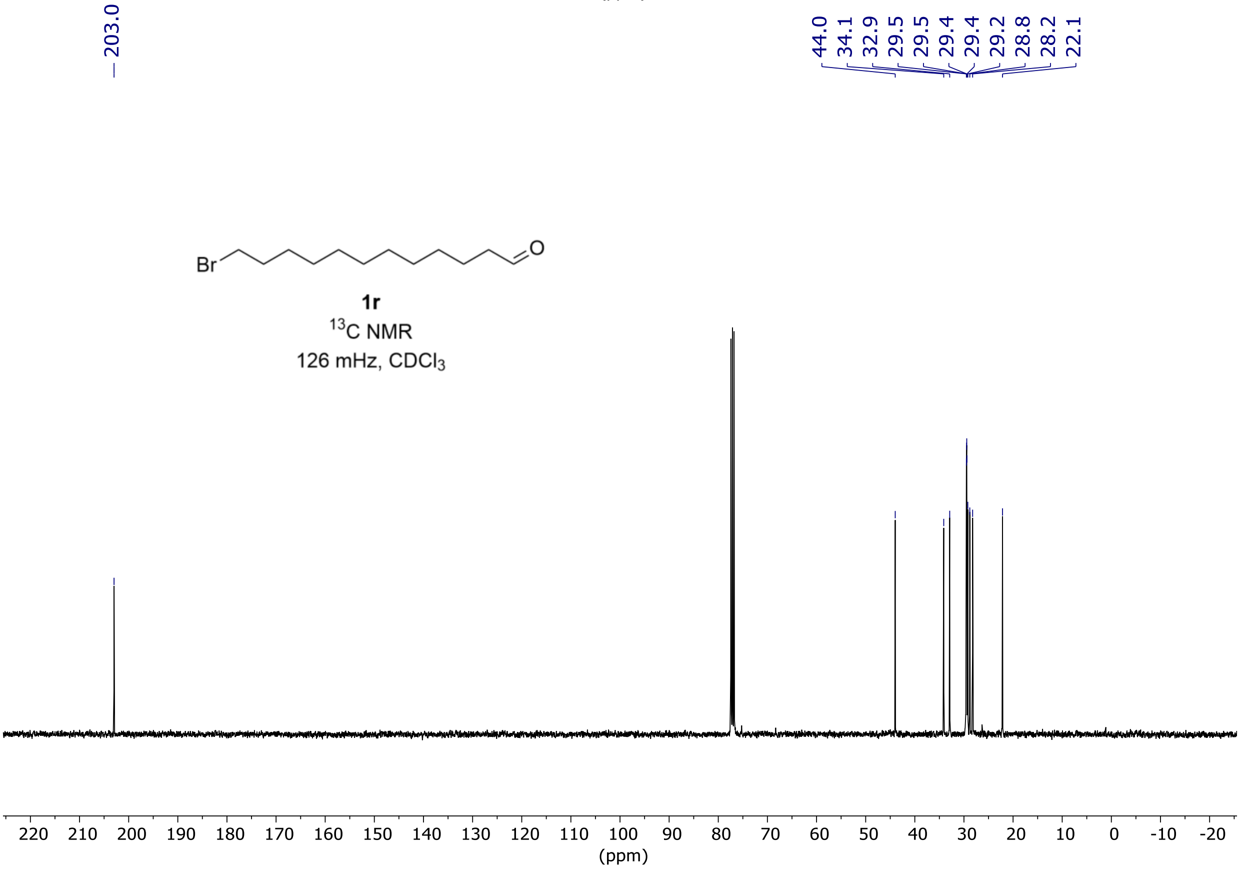
203.0
44.0
34.1
32.9
29.5
29.5
29.4
29.4
29.2
28.8
28.2
22.1
Br
O
1r
^{13}C NMR
126 mHz, $CDCl_3$
220 210 200 190 180 170 160 150 140 130 120 110 100 90 80 70 60 50 40 30 20 10 0 -10 -20
(ppm)

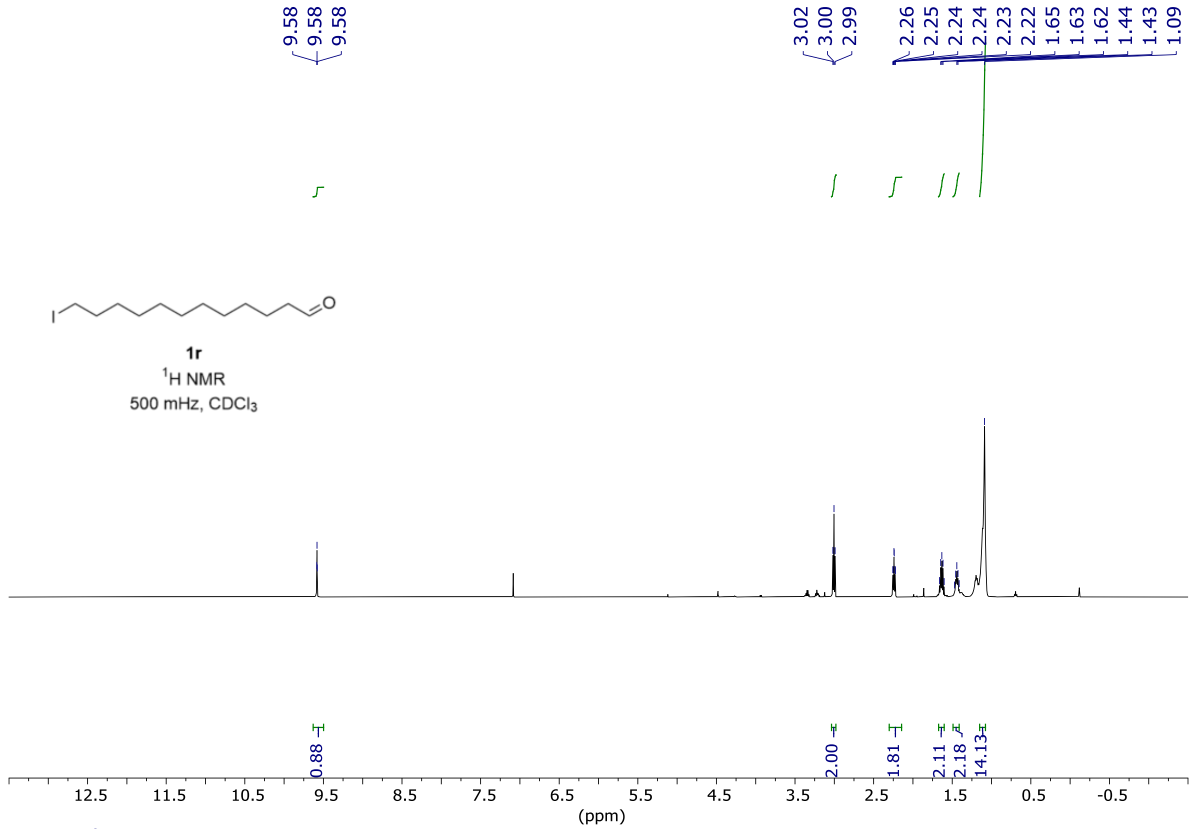

1r

^{1}H NMR

500 mHz, $CDCl_3$

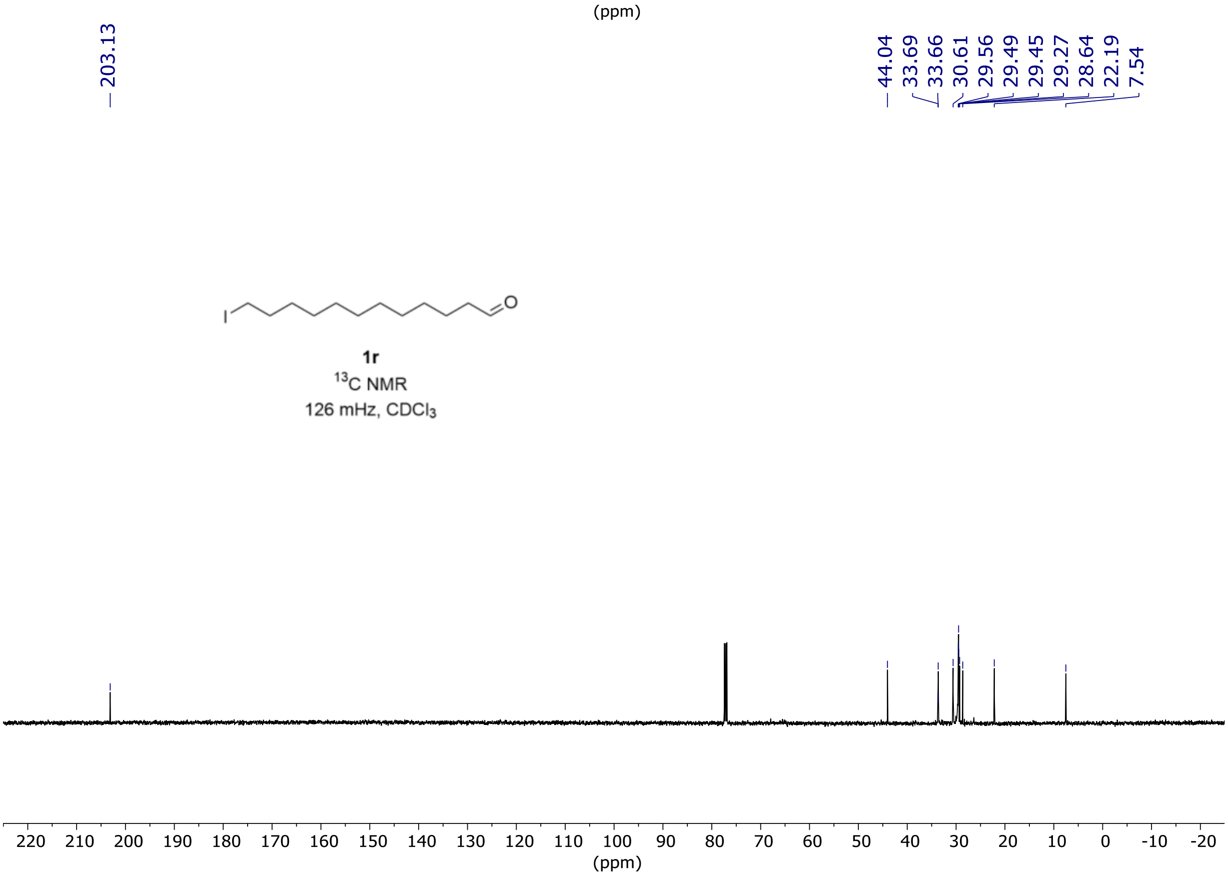

1r

^{13}C NMR

126 mHz, $CDCl_3$

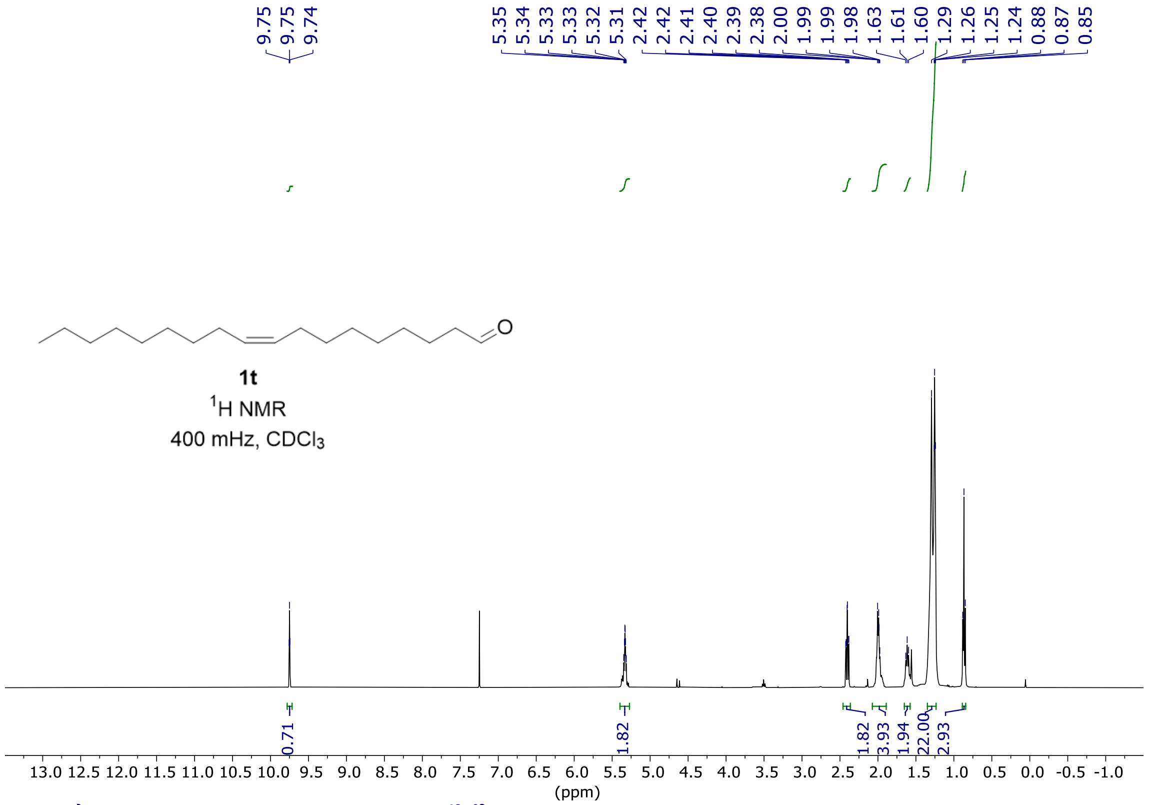
9.75
9.75
9.74
5.35
5.34
5.33
5.33
5.32
5.31
2.42
2.42
2.41
2.40
2.39
2.38
2.00
1.99
1.99
1.98
1.63
1.61
1.60
1.29
1.26
1.25
1.24
0.88
0.87
0.85
1t
1H NMR
400 mHz, CDCl3
0.71
1.82
1.82
3.93
1.94
22.00
2.93
(ppm)

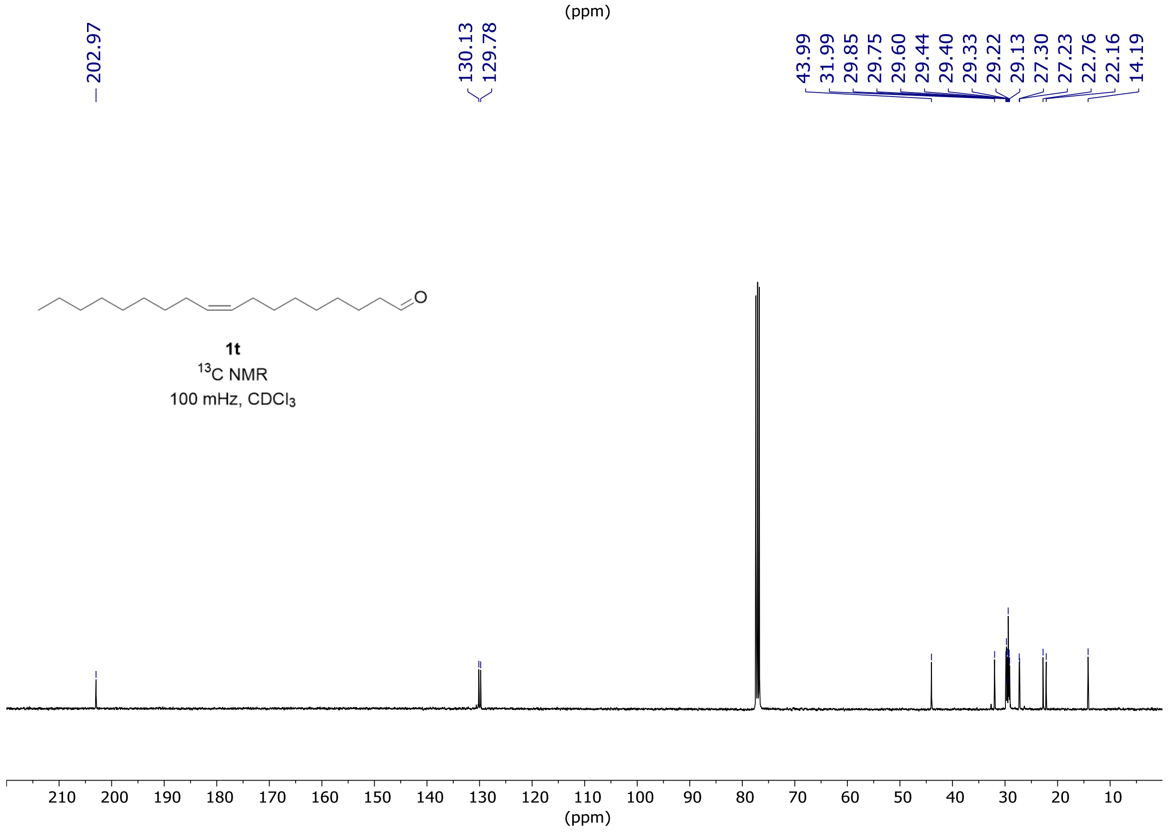
202.97
130.13
129.78
43.99
31.99
29.85
29.75
29.60
29.44
29.40
29.33
29.22
29.13
27.30
27.23
22.76
22.16
14.19
1t
13C NMR
100 mHz, CDCl3
(ppm)

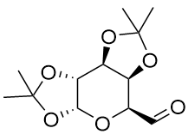

1u

^{1}H NMR

400 mHz, $CDCl_3$

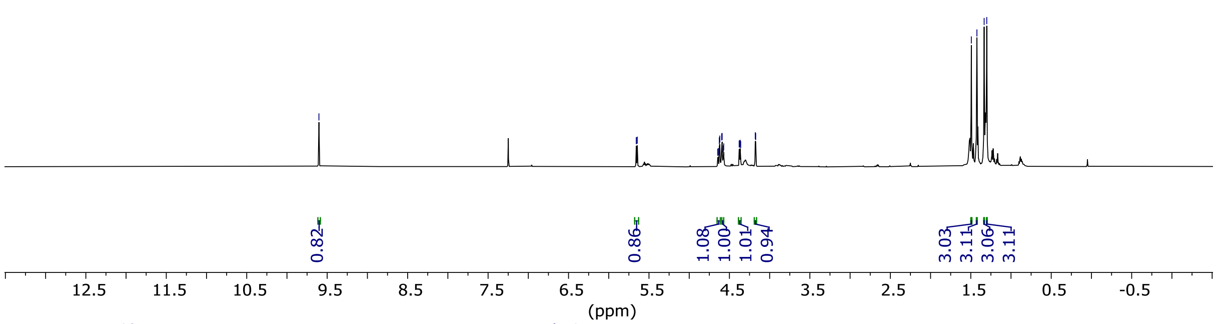

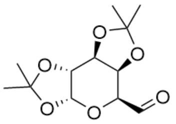

1u

^{13}C NMR

100 mHz, $CDCl_3$

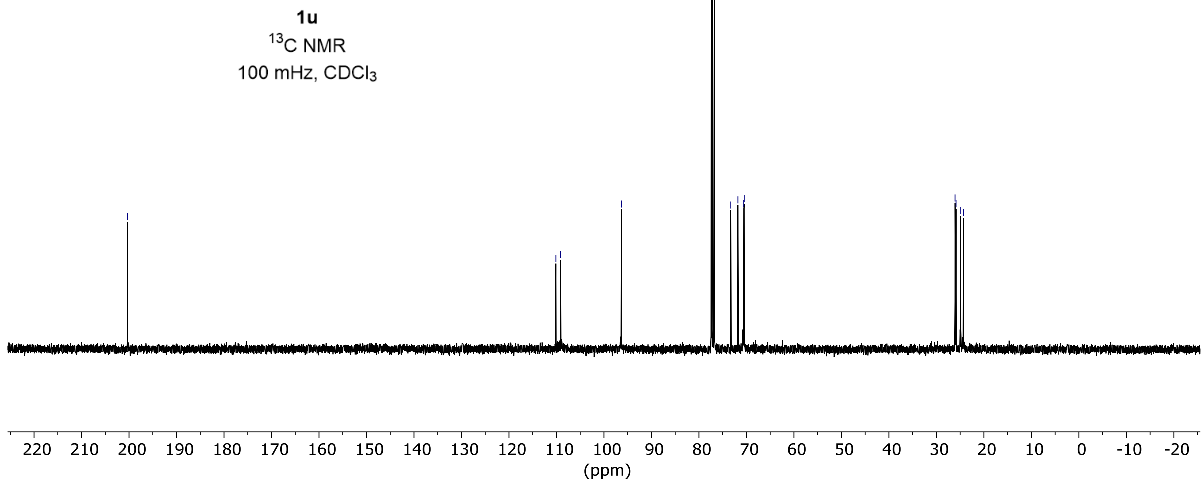

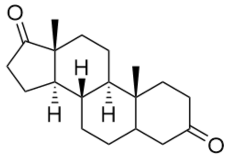

1v

^{1}H NMR

500 mHz, $CDCl_3$

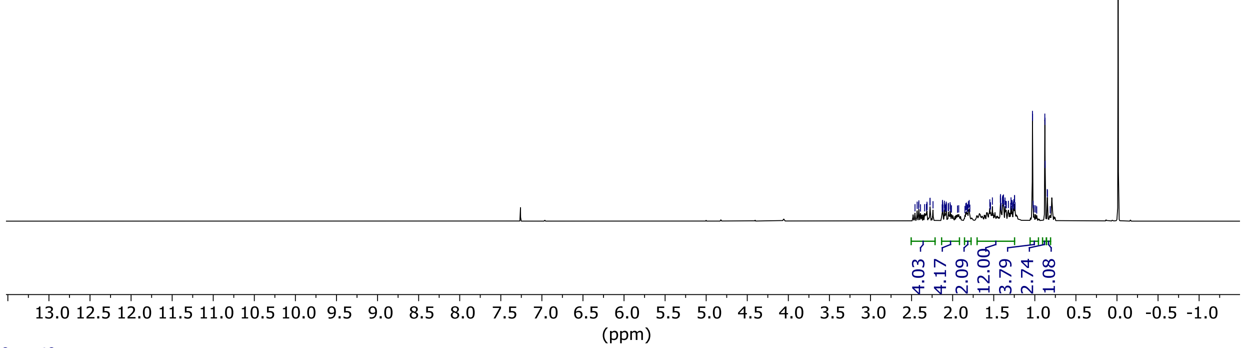

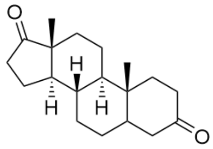

1v

^{13}C NMR

126 mHz, $CDCl_3$

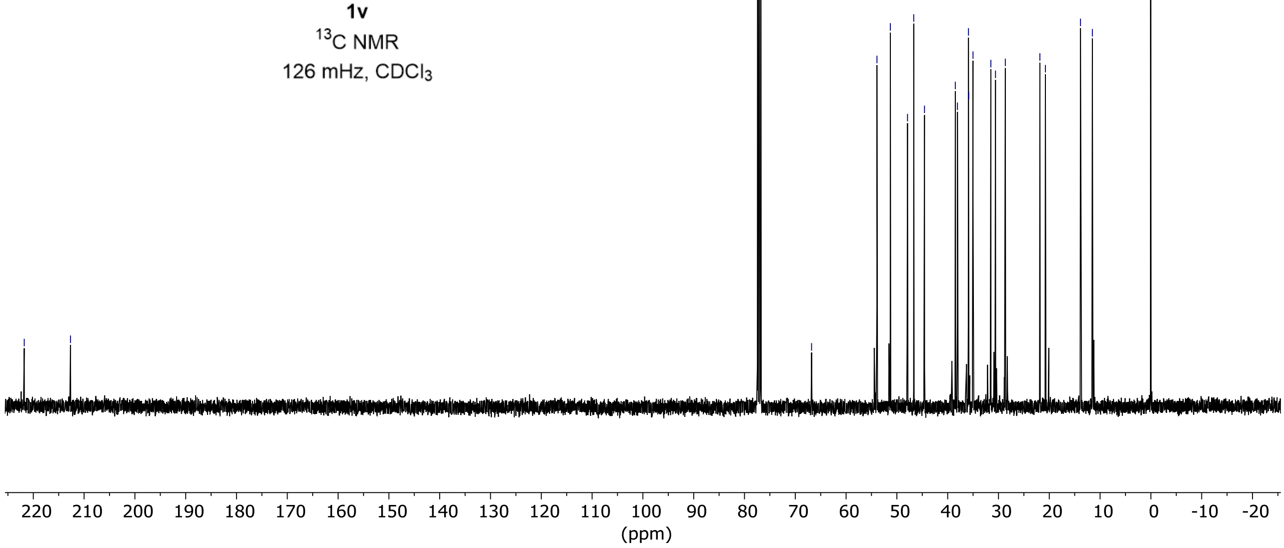

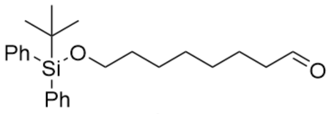

1w

^{1}H NMR

500 mHz, $CDCl_3$

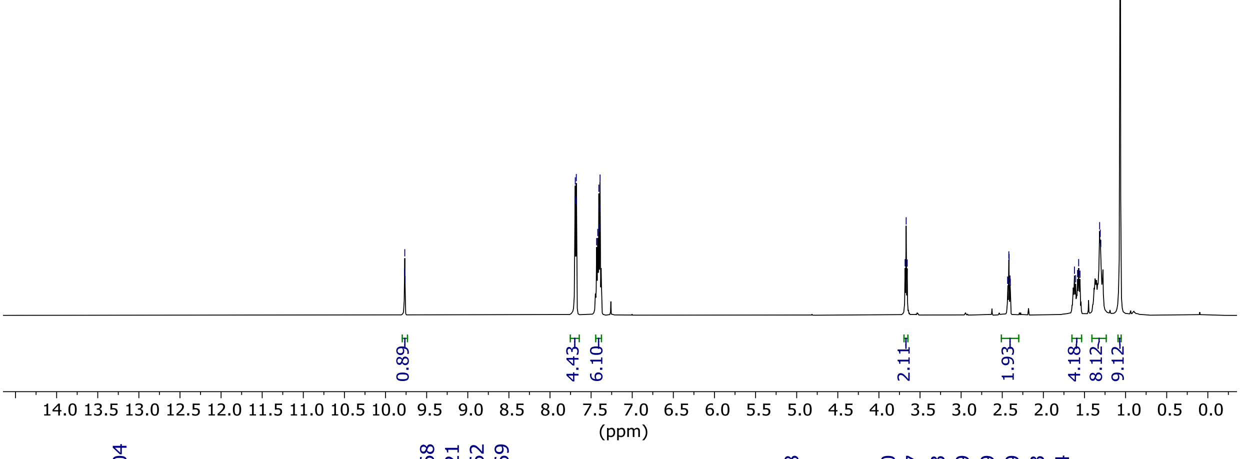

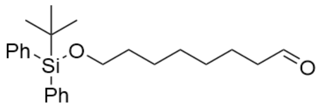

1w

^{13}C NMR

126 mHz, $CDCl_3$

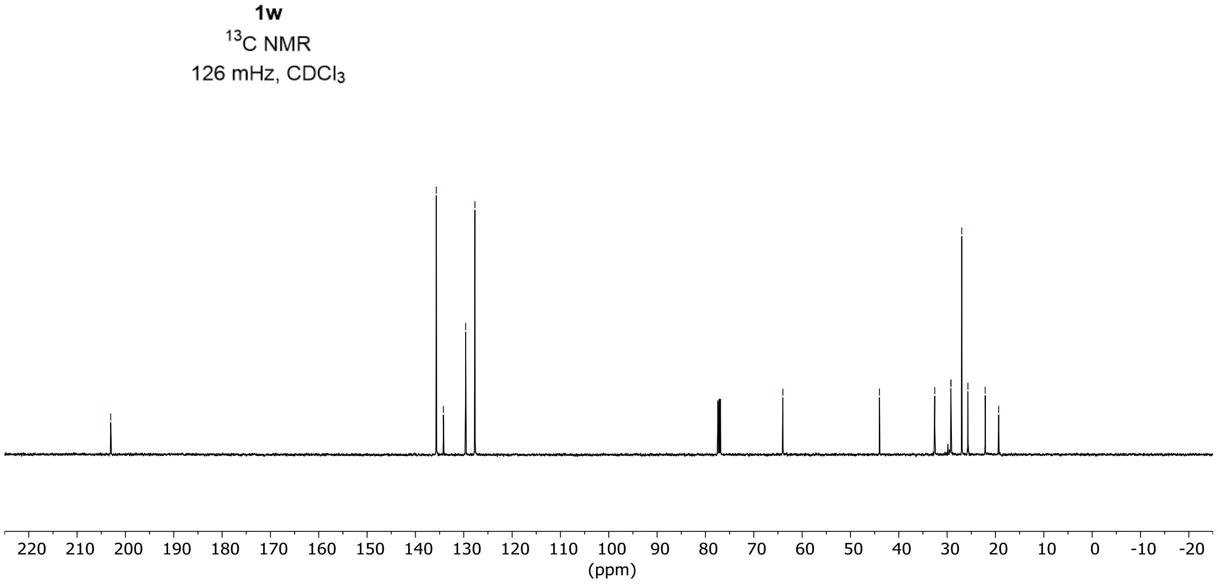

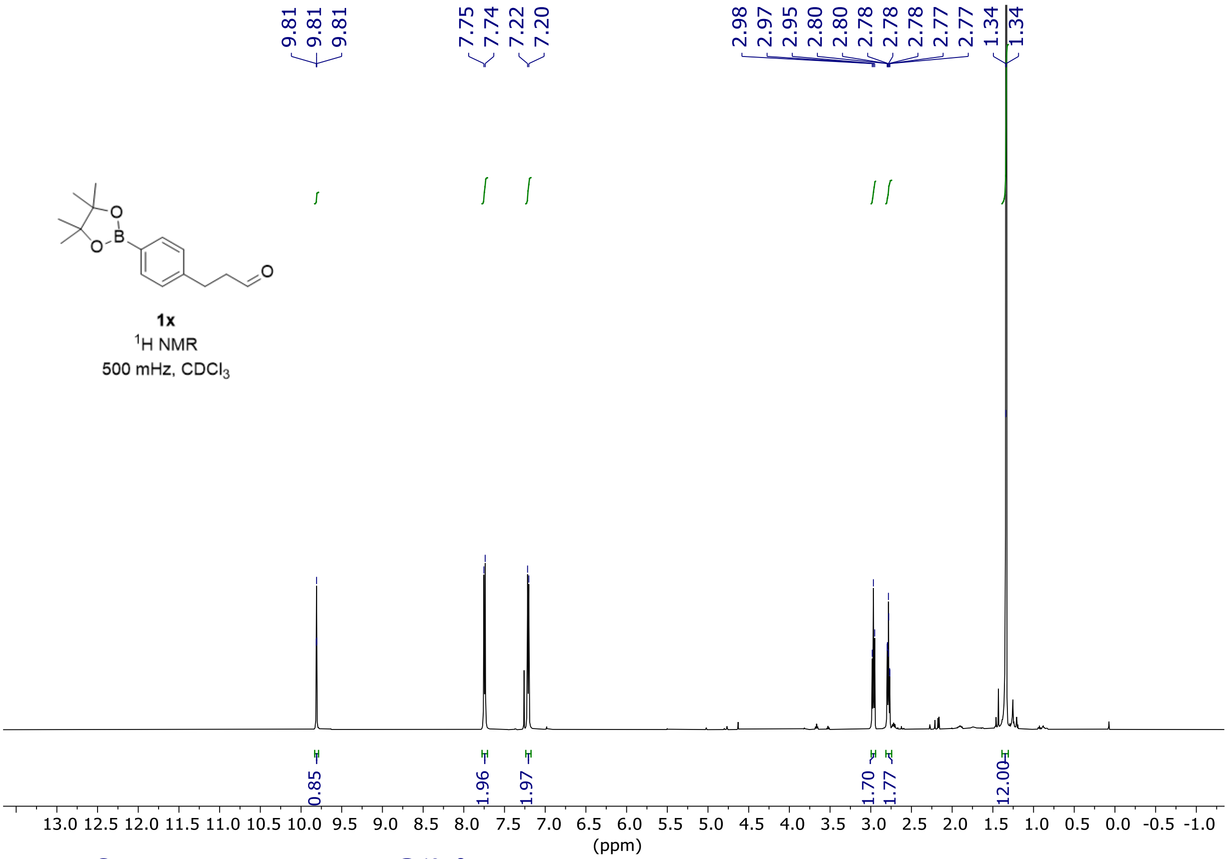
9.81
9.81
9.81
7.75
7.74
7.22
7.20
2.98
2.97
2.95
2.80
2.80
2.78
2.78
2.78
2.77
2.77
1.34
1.34
1x
1H NMR
500 mHz, CDCl3
0.85
1.96
1.97
1.70
1.77
12.00
13.0 12.5 12.0 11.5 11.0 10.5 10.0 9.5 9.0 8.5 8.0 7.5 7.0 6.5 6.0 5.5 5.0 4.5 4.0 3.5 3.0 2.5 2.0 1.5 1.0 0.5 0.0 -0.5 -1.0
(ppm)

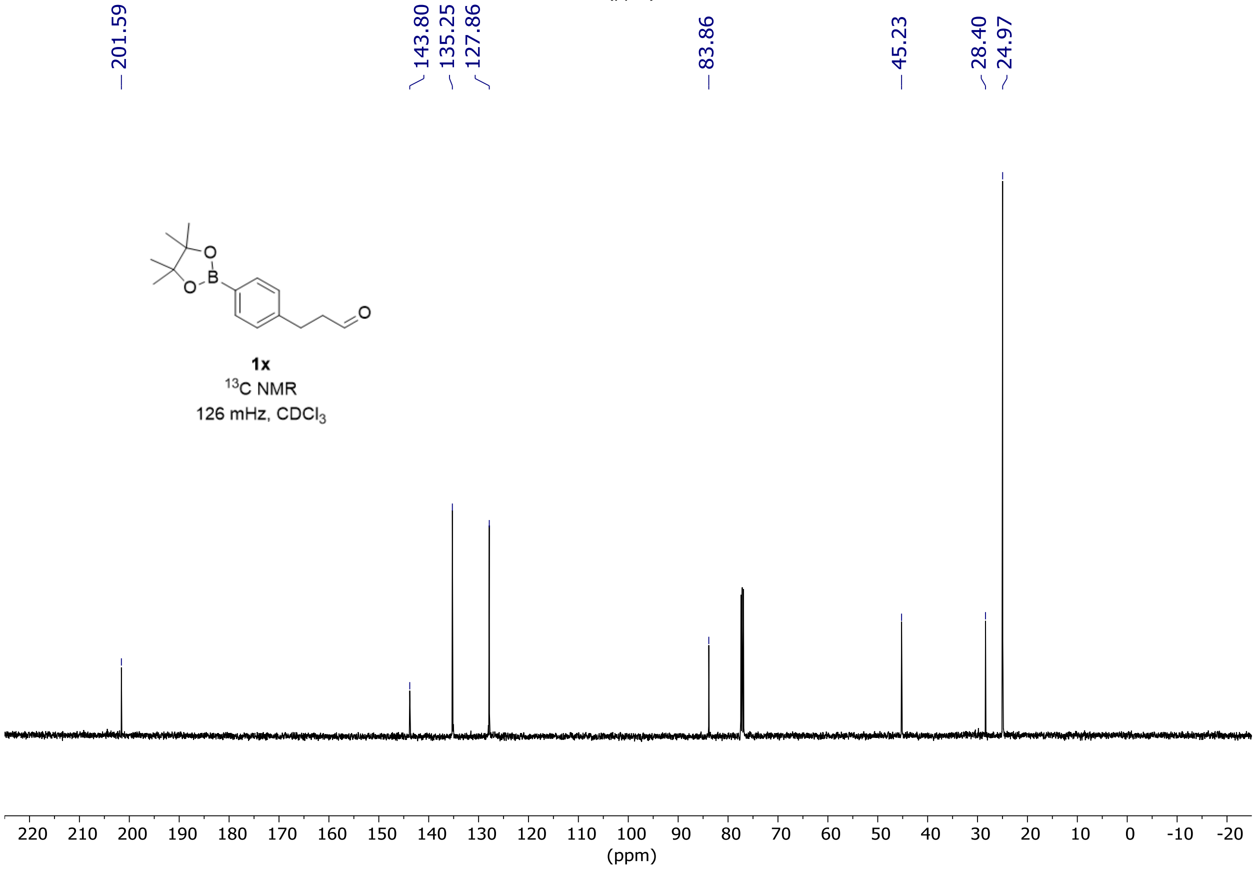
201.59
143.80
135.25
127.86
83.86
45.23
28.40
24.97
1x
13C NMR
126 mHz, CDCl3
220 210 200 190 180 170 160 150 140 130 120 110 100 90 80 70 60 50 40 30 20 10 0 -10 -20
(ppm)

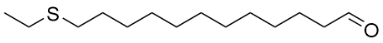

1y

^{13}C NMR

126 mHz, $CDCl_3$

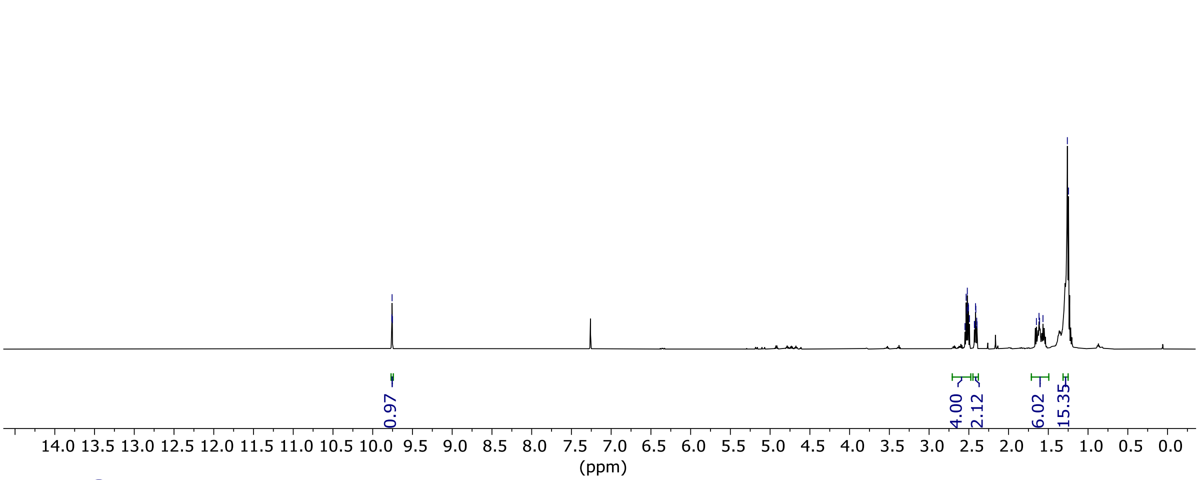

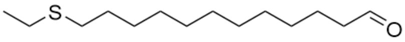

1y

^{13}C NMR

126 mHz, $CDCl_3$

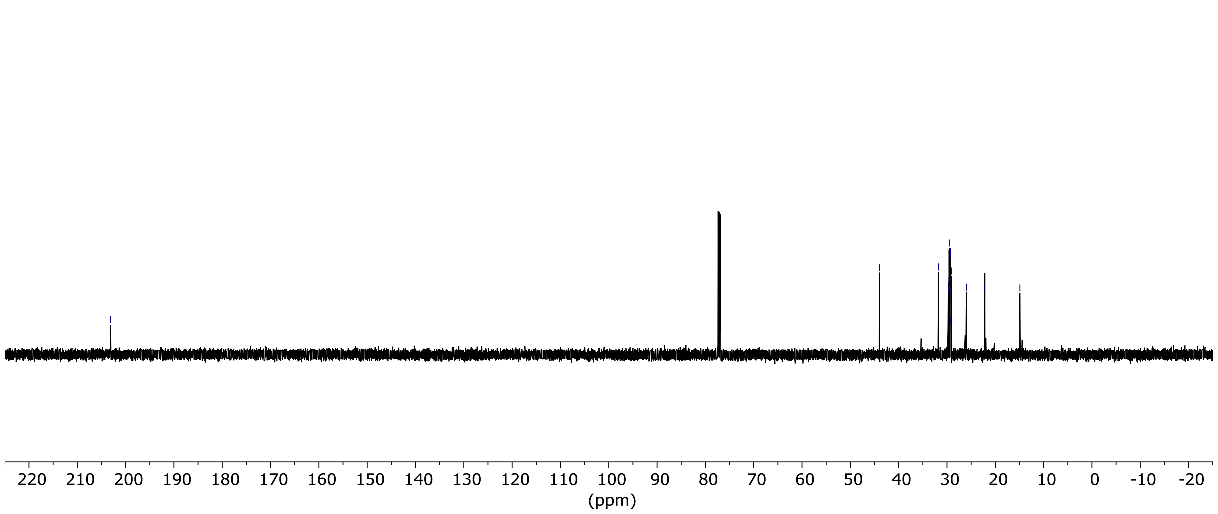

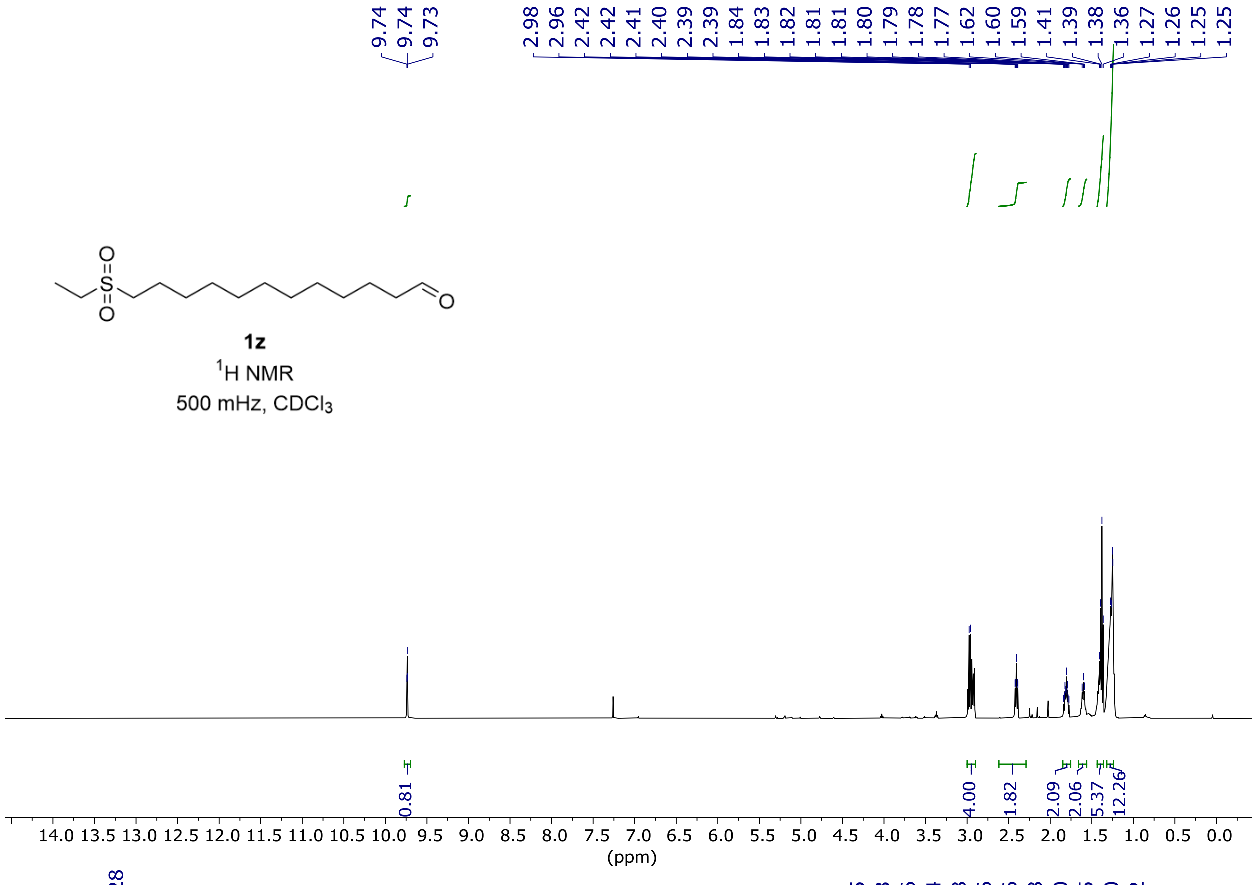

1z
^{1}H NMR
500 mHz, $CDCl_3$

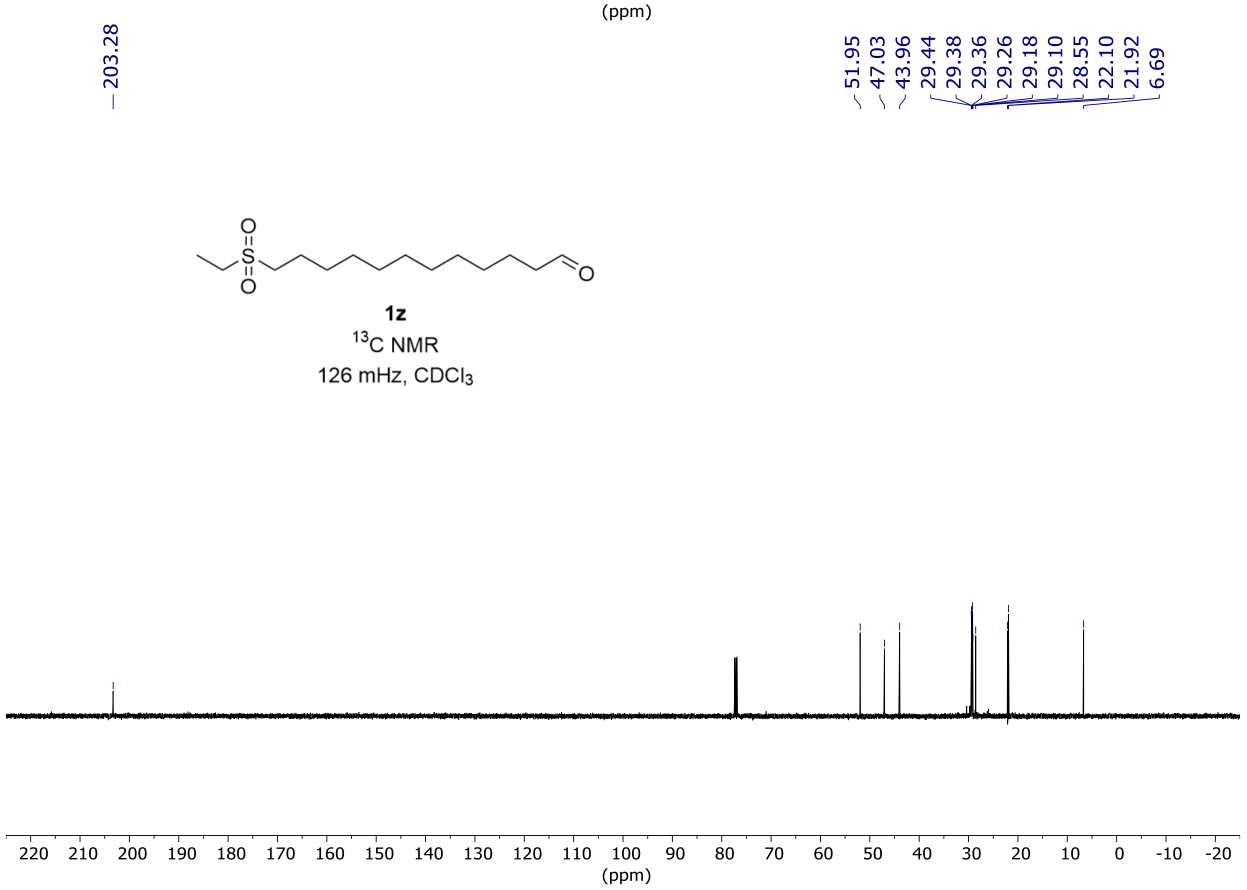

1z
^{13}C NMR
126 mHz, $CDCl_3$

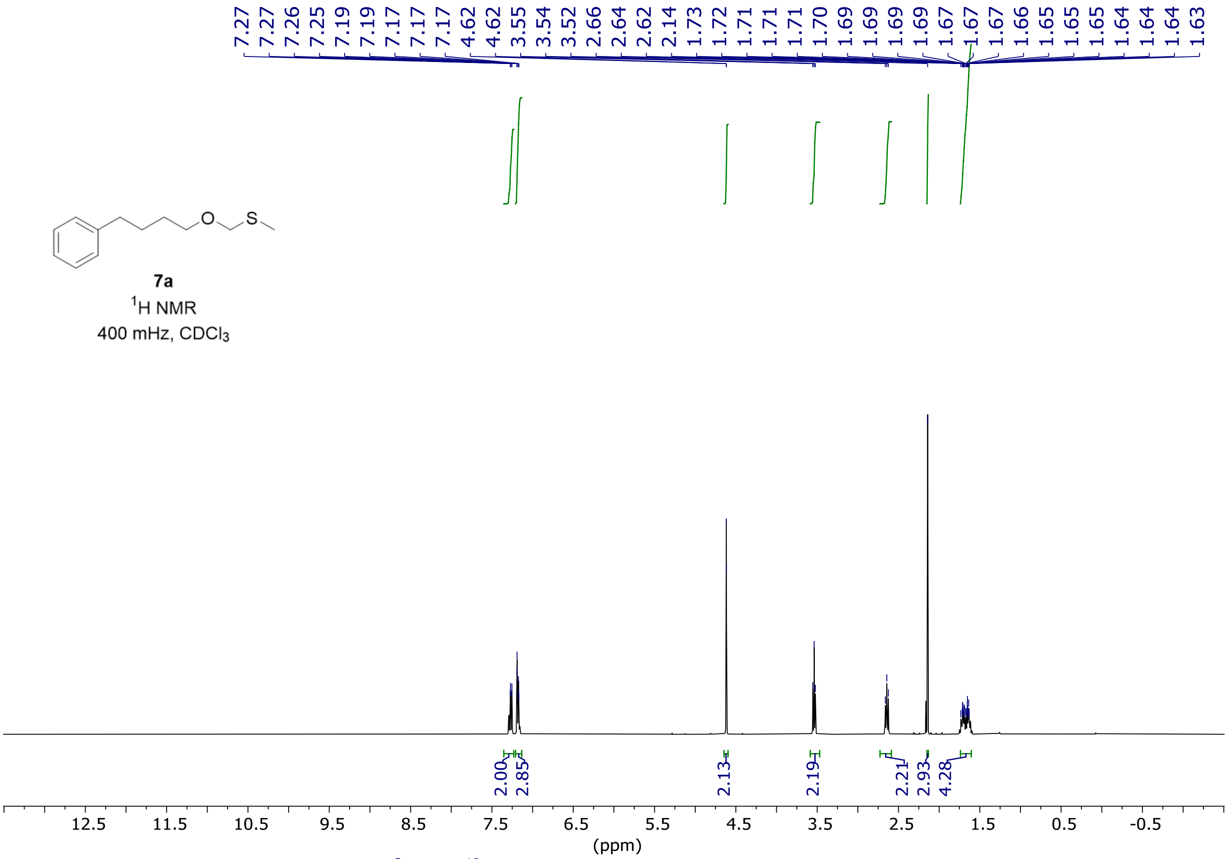
7.27 7.27 7.26 7.25 7.19 7.19 7.17 7.17 7.17 4.62 4.62 3.55 3.54 3.52 2.66 2.64 2.62 2.14 1.73 1.72 1.71 1.71 1.71 1.70 1.69 1.69 1.69 1.69 1.67 1.67 1.67 1.66 1.65 1.65 1.65 1.64 1.64 1.64 1.63
O
S
7a
1H NMR
400 mHz, CDCl3
2.00 2.85 2.13 2.19 2.21 2.93 4.28
12.5 11.5 10.5 9.5 8.5 7.5 6.5 5.5 4.5 3.5 2.5 1.5 0.5 -0.5
(ppm)

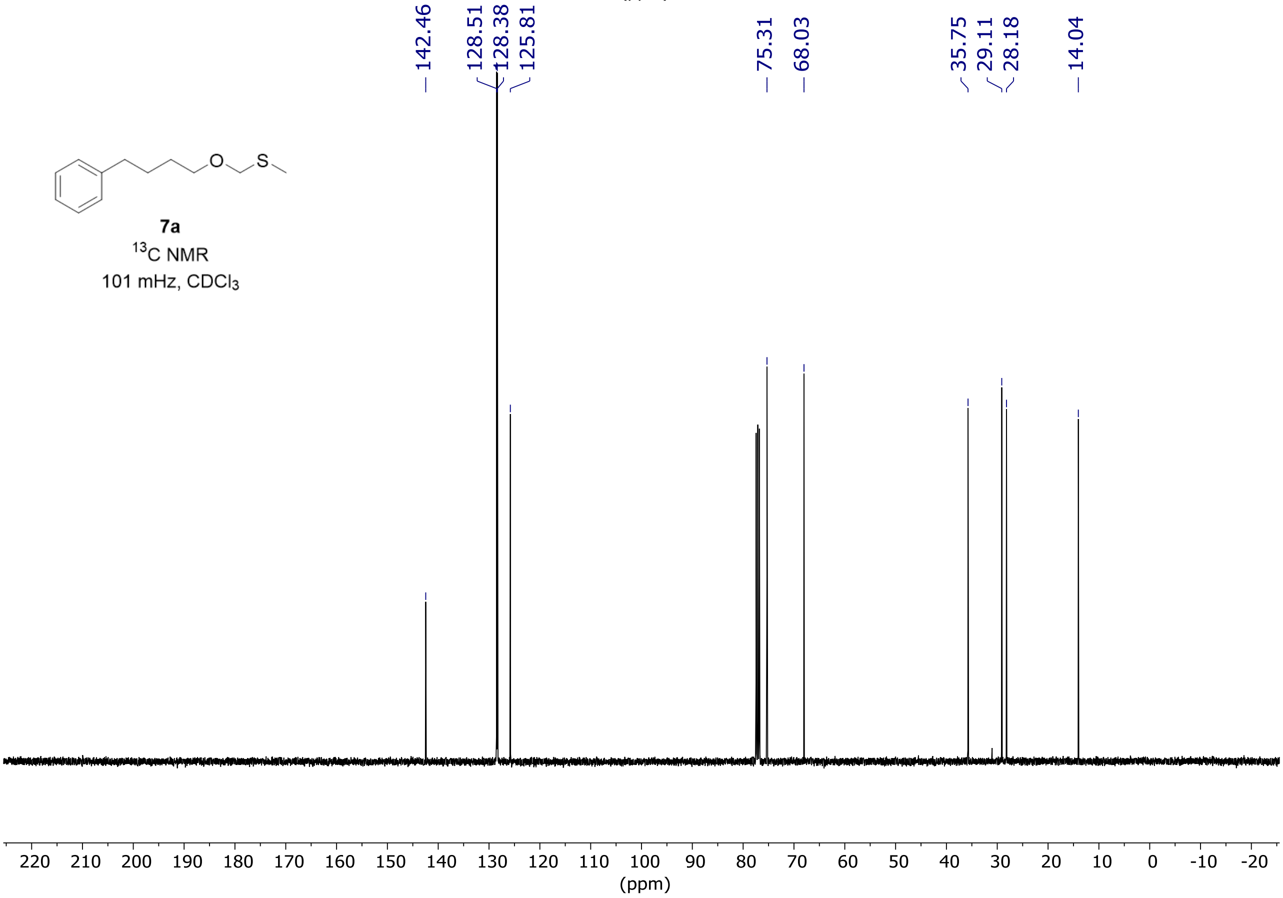
142.46 128.51 128.38 125.81 75.31 68.03 35.75 29.11 28.18 14.04
O
S
7a
13C NMR
101 mHz, CDCl3
220 210 200 190 180 170 160 150 140 130 120 110 100 90 80 70 60 50 40 30 20 10 0 -10 -20
(ppm)

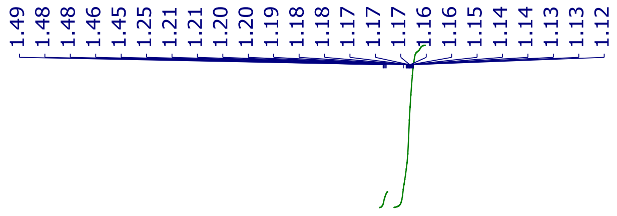

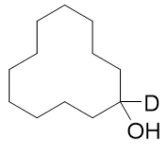

6a

^{1}H NMR

500 mHz, $CDCl_3$

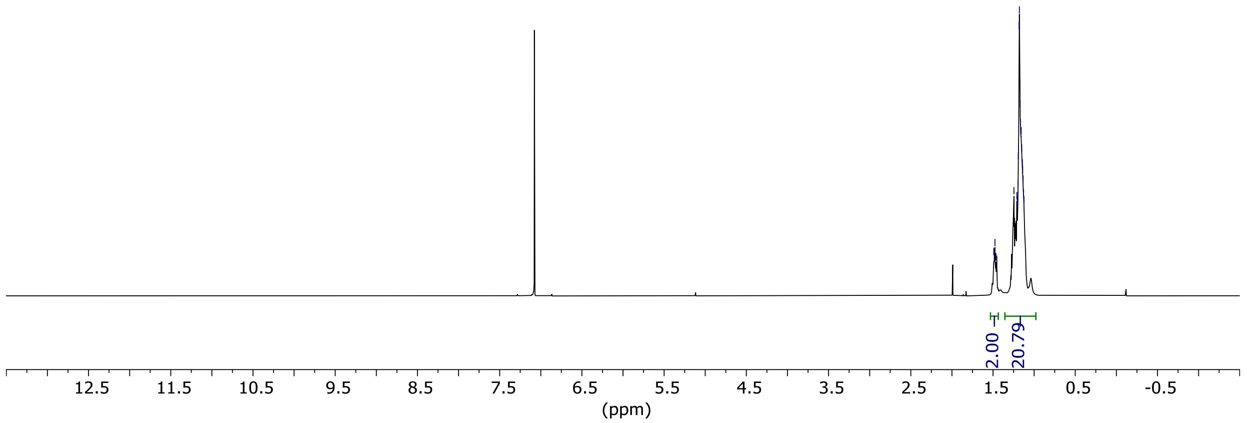

Supplement: SC-016-D5SC06546A-s001 [file SC-016-D5SC06546A-s001.pdf]
